# Genetic identification of medullary neurons underlying congenital hypoventilation

**DOI:** 10.1126/sciadv.adj0720

**Published:** 2024-06-19

**Authors:** Ke Cui, Yiling Xia, Abhisarika Patnaik, Aikaterini Salivara, Elijah D. Lowenstein, Eser G. Isik, Adrian L. Knorz, Laura Airaghi, Michela Crotti, Alistair N. Garratt, Fanqi Meng, Dietmar Schmitz, Michèle Studer, Filippo M. Rijli, Hans G. Nothwang, Benjamin R. Rost, Ulf Strauß, Luis R. Hernandez-Miranda

**Affiliations:** ^1^ The Brainstem Group, Institute for Cell Biology and Neurobiology, Charité Universitätsmedizin Berlin, corporate member of Freie Universität Berlin and Humboldt-Universität zu Berlin, Berlin, Germany.; ^2^ German Center for Neurodegenerative Diseases (DZNE), 10117 Berlin, Germany.; ^3^ Charité Universitätsmedizin Berlin, corporate member of Freie Universität Berlin and Humboldt-Universität zu Berlin, Berlin, Germany.; ^4^ Max-Delbrück-Centrum in the Helmholtz Association, Berlin, Germany.; ^5^ Université Côte d'Azur (UCA), CNRS, Inserm, Institute of Biology Valrose (iBV), Nice, France.; ^6^ Laboratory of Developmental Neuroepigenetics, Friedrich Miescher Institute for Biomedical Research, Basel, Switzerland.; ^7^ University of Basel, Basel, Switzerland.; ^8^ Division of Neurogenetics, Cluster of Excellence Hearing4all, Carl von Ossietzky University, Oldenburg, Germany.; ^9^ Institute for Cell Biology and Neurobiology, Charité Universitätsmedizin Berlin, corporate member of Freie Universität Berlin and Humboldt-Universität zu Berlin, Berlin, Germany.

## Abstract

Mutations in the transcription factors encoded by *PHOX2B* or *LBX1* correlate with congenital central hypoventilation disorders. These conditions are typically characterized by pronounced hypoventilation, central apnea, and diminished chemoreflexes, particularly to abnormally high levels of arterial PCO_2_. The dysfunctional neurons causing these respiratory disorders are largely unknown. Here, we show that distinct, and previously undescribed, sets of medullary neurons coexpressing both transcription factors (dB2 neurons) account for specific respiratory functions and phenotypes seen in congenital hypoventilation. By combining intersectional chemogenetics, intersectional labeling, lineage tracing, and conditional mutagenesis, we uncovered subgroups of dB2 neurons with key functions in (i) respiratory tidal volumes, (ii) the hypercarbic reflex, (iii) neonatal respiratory stability, and (iv) neonatal survival. These data provide functional evidence for the critical role of distinct medullary dB2 neurons in neonatal respiratory physiology. In summary, our work identifies distinct subgroups of dB2 neurons regulating breathing homeostasis, dysfunction of which causes respiratory phenotypes associated with congenital hypoventilation.

## Teaser

Medullary dB2 neurons are key for neonatal breathing.

## INTRODUCTION

Breathing homeostasis originates from complex networks of medullary neurons that generate the respiratory rhythm, provide modulatory input, and monitor tissue gas levels. Genetic and environmental factors contribute to the inception of hypoventilation disorders, but the affected neural circuits are largely unknown. One of these disorders, congenital central hypoventilation syndrome (CCHS; OMIM 209880) is a life-threatening condition with a severe presentation of respiratory and autonomous nervous system dysregulation (*1*–*4*). This condition is classically diagnosed in newborns and is characterized by primary alveolar hypoventilation and central apnea during sleep (*5*, *6*). Cessation of breathing occurs less frequently than hypoventilation in patients with CCHS. However, severely affected individuals suffer from alveolar hypoventilation or spontaneous respiratory arrest regardless of their arousal state (*7*, *8*). Affected patients also display attenuated or absent central and peripheral chemoreceptor responses to changes in tissue gas levels. In this context, patients with CCHS are unable to adjust automatically their breathing in response to abnormally high levels of arterial PCO_2_ and/or low levels of PO_2_ (*9*, *10*).

CCHS is unique in the sense that a clear genetic alteration has been identified to be causative in this disorder (*8*, *11*–*14*). In this context, patients with CCHS present with dominant de novo mutations in *PHOX2B* (*8*, *11*–*13*, *15*, *16*), a gene that encodes for a homeodomain transcription factor essential for the development and function of central and peripheral visceral neurons (*17*–*22*). As expected, patients with CCHS also have variable manifestations of peripheral autonomic nervous system dysregulation, including Hirschsprung’s disease (a rare disorder that produces aganglionosis of the distal hindgut) or neural-crest tumors (*1*, *4*, *5*, *15*, *23*–*25*). Mouse models carrying human *PHOX2B* mutations die during early embryonic life or soon after birth (*26*, *27*). To date, the dysfunctional neural circuit responsible for the respiratory deficits observed in CCHS remains unknown. Previously, we identified a recessive frameshift mutation (termed *LBX1^FS^*) in the gene encoding the homeodomain transcription factor LBX1 that causes severe congenital hypoventilation and other respiratory phenotypes that resemble CCHS, without producing autonomic dysregulation (*28*). Homozygous *Lbx1^FS^* mutant mice die immediately at birth from an apparent failure to breathe and seem to display a unique anatomical deficit in the development of at least two medullary neuron groups that coexpress both Lbx1 and Phox2b, which locate to the retrotrapezoid nucleus and to the dorsal medulla (*28*).

In mice, *Lbx1* is essential for the specification of four distinct medullary neuron types known as dB1, dB2, dB3, and dB4 (*29*–*33*). Notably, dB2 neurons are the only neuron type in the entire nervous system that coexpress Lbx1 and Phox2b during development (*28*, *31*, *32*). These neurons originate from a discrete pool of Phox2b^+^ progenitor cells called the dB2 progenitor domain, which resides transiently between rhombomeres 2 and 6 [reviewed in (*30*, *33*)]. As these progenitors become postmitotic, they switch on the expression of *Lbx1* and predictably migrate to distinct locations in the brainstem. In our previous work, we showed that the *Lbx1^FS^* mutation spares most functions known for the Lbx1 wild-type protein, but it selectively precludes a productive cooperativity with Phox2b to specify dB2 neurons, such as those that populate the retrotrapezoid nucleus or the dorsal medulla (*28*). Although most neurons emanating from the dB2 progenitor domain have not been systematically characterized, the conditional restriction of the *Lbx1^FS^* mutation to Phox2b-expressing cells (in *Phox2b^Cre/+^;Lbx1^FS/lox^* mice) recapitulates the severe respiratory phenotype seen in homozygous *Lbx1^FS^* animals (*28*). This includes pronounced gasping behavior and lethality immediately after birth, although it should be noted that a limited number of mutant mice survive for up to 2 hours while displaying robust hypoventilation and marked apneic behavior. This suggests that deficits in dB2 neuron function might cause hypoventilation and some respiratory phenotypes associated with CCHS. However, the immediate death of homozygous *Lbx1^FS^* and conditional *Phox2b^Cre/+^;Lbx1^FS/lox^* mice precluded the definitive association of dB2 neuron dysfunction with respiratory control. Similarly, it is presently unknown whether all or specific subgroups of dB2 neurons (i.e., dB2 neurons originating from distinct rhombomeres) participate in breathing.

In this study, we set out to investigate the potential role of dB2 neurons in neonatal respiration. Using murine intersectional chemogenetics, we show that these neurons are essential for the generation of adequate respiratory tidal volumes (amount of air inhaled per breath). Furthermore, our experiments illustrate that dB2 neurons have prominent functions in respiratory frequency patterns, respiratory stability, and the hypercarbic reflex in neonates. By restricting the disease-causing *Lbx1^FS^* variant to specific rhombomeres, we show that a single subgroup of dB2 neurons (from rhombomere 5) is crucial for deficits in respiratory frequency and the response to hypercarbia in neonates, while agenesis of distinct subgroups of dB2 neurons (generated in rhombomere 6) causes (i) reduced respiratory tidal volumes, (ii) neonatal respiratory instability, and (iii) neonatal mortality. Thus, our work uncovers previously undescribed medullary neurons with key functions in the central control of breathing.

## RESULTS

### dB2 neurons are distributed in the pons and medulla

**Fig. 1.**
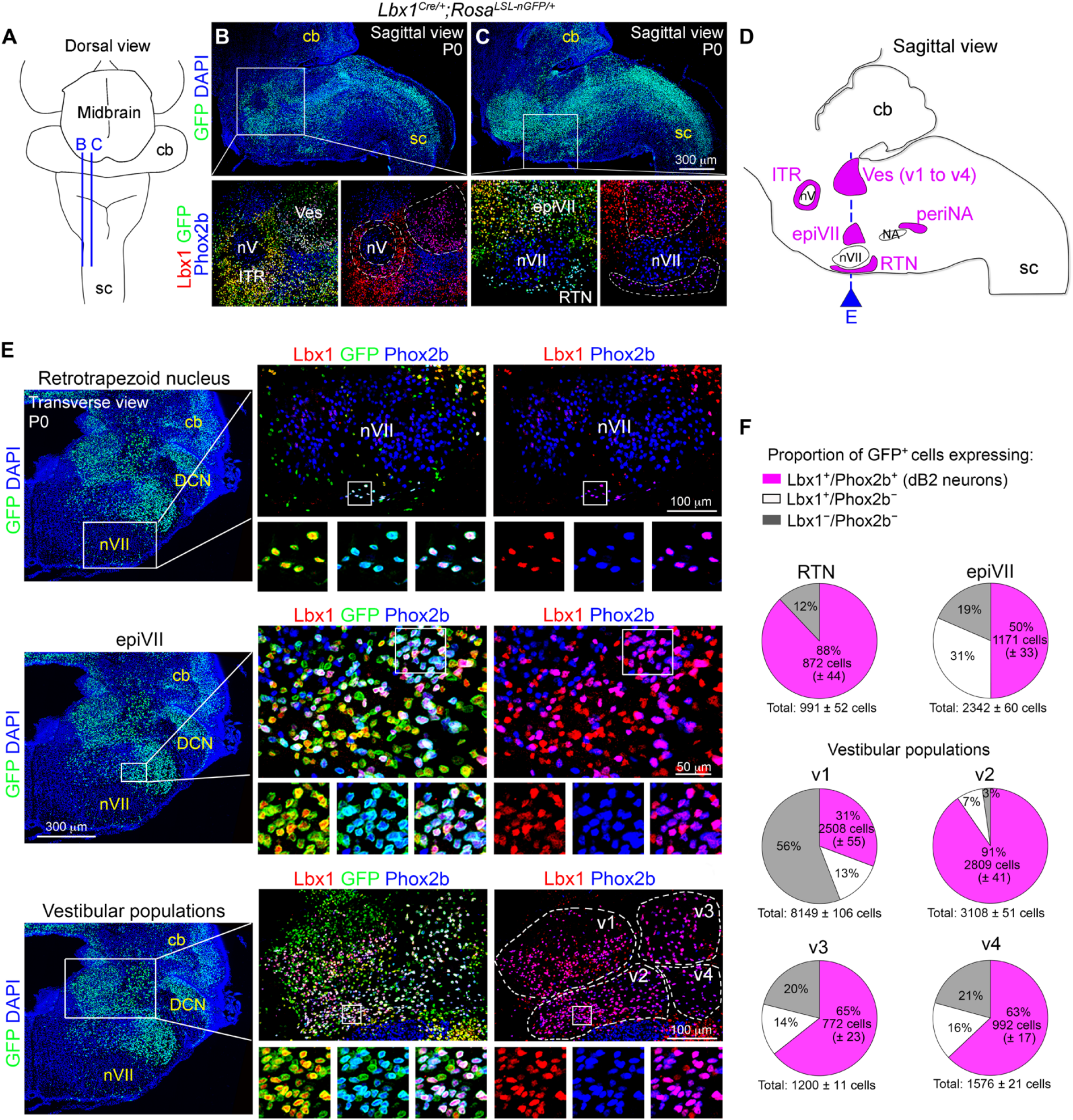
Identification of Lbx1^+^/Phox2b^+^brainstem neurons. (**A**) Schematic view of a mouse brainstem at birth. Blue lines denote the sagittal planes illustrated in (B) and (C). The spinal cord (sc) and cerebellum (cb) are labeled. (**B** and **C**) Sections taken from *Lbx1^Cre/+^;Rosa^LSL-nGFP/+^* mice at birth (P0). Top displays GFP (green) and 4′,6-diamidino-2-phenylindole (DAPI) (blue) signals. Boxed areas, magnified at the bottom, show Lbx1 (red, false color), GFP and Phox2b (blue, false color) merged signals (left), or Lbx1 and Phox2b signals (right) for better visualization of Lbx1^+^/Phox2b^+^ (magenta) neurons. (**D**) Schematic view of a P0 mouse brainstem illustrating the location of Lbx1^+^/Phox2b^+^ neuron subgroups (magenta) identified here: (i) intertrigeminal (ITR), vestibular (Ves), epifacial (epiVII), retrotrapezoid nucleus (RTN), and peri nucleus ambiguus (periNA) neurons. The trigeminal (nV), facial (nVII), and ambiguus (NA) motor nuclei are labeled for orientation. The blue line denotes the transverse plane illustrated in (E). For analysis of ITR and periNA cells, see fig. S2. (**E**) Left: A brainstem section stained with GFP (green) and DAPI (blue) at birth. Here, six subgroups of Lbx1^+^/Phox2b^+^ neurons can be identified: retrotrapezoid nucleus, epiVII, and vestibular neurons. Boxed areas, magnified on the right, show Lbx1 (red, false color), GFP and Phox2b (blue, false color) merged signals (middle), or Lbx1 and Phox2b signals (right) for a better visualization of Lbx1^+^/Phox2b^+^ (magenta) cells. The small boxed areas are magnified at the bottom of the main photographs. Note that four vestibular Lbx1^+^/Phox2b^+^ neuron subgroups can be distinguished (v1 to v4), see also figs. S2E and S3C. (**F**) Pie charts illustrating the proportion of neurons with a history of Lbx1 expression (GFP^+^) and active expression of Lbx1 and Phox2b. The actual quantification of these cells (*n* = 4 mice) can be found in fig. S2D. Tabulated data can be found in data S1.

Except for the retrotrapezoid (also known as parafacial) nucleus and the intertrigeminal (or peritrigeminal) region, the location of most dB2 neurons has not been conclusively determined (*26*, *28*, *34*–*37*). To define their spatial distribution, we first mapped the brainstem of mice with Lbx1 and Phox2b antibodies. Specifically, we used *Lbx1^Cre^* newborn mice carrying the *Rosa^LSL-nGFP^* (*Lbx1^Cre/+^;Rosa^LSL-nGFP/+^* mice) allele, a reporter that expresses green fluorescent protein (GFP) in the nuclear membrane after Cre-mediated recombination. We used this genetic fate mapping strategy as many Lbx1-derived neurons down-regulate this factor in perinatal life (fig. S1). This analysis uncovered eight major subgroups of Lbx1^+^/Phox2b^+^ neurons that locate from cranial to caudal to (i) the intertrigeminal region, (ii) the vestibular nuclei [v1 to v4 neurons; previously defined by us as dorsal dB2 neurons; c.f. (*28*)], (iii) the retrotrapezoid nucleus, (iv) the dorsal part of the facial motor nucleus (here called epiVII), and (v) the lateral part of the nucleus ambiguus (here called periNA) (Fig. 1 and fig. S2). We conclude that most Lbx1^+^/Phox2b^+^ (dB2) neurons locate to the rostral medulla (vestibular, retrotrapezoid nucleus, and epiVII) and pons (intertrigeminal), but at least one subgroup of these cells migrates into the caudal medulla (periNA) (schematically illustrated in Fig. 1D and fig. S2E).

**Fig. 2.**
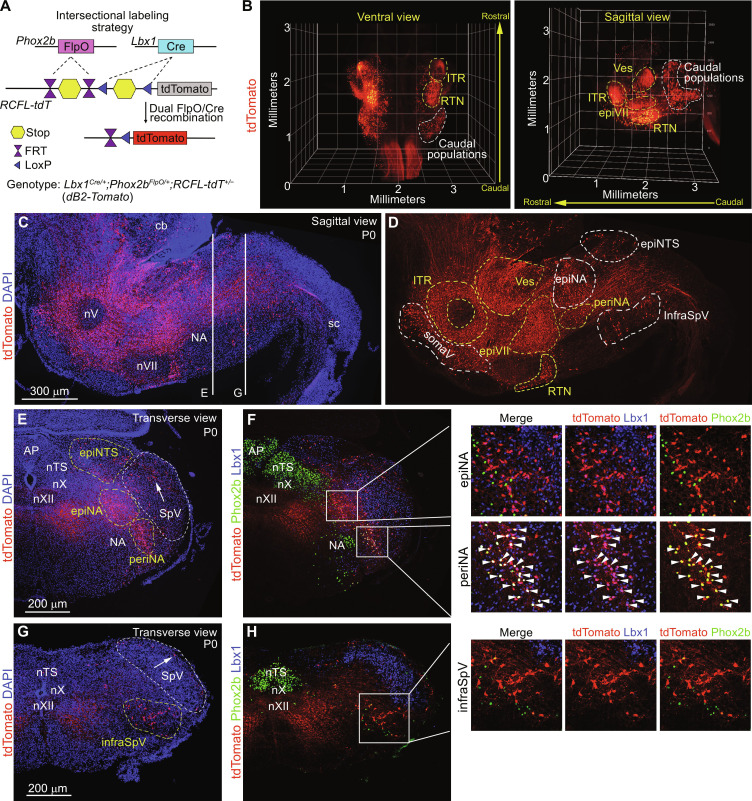
Intersectional labeling of dB2 neurons. (**A**) Strategy to mark neurons with a history of Lbx1 and Phox2b expression using tdTomato fluorescence. The resulting *dB2-Tomato* genotype is indicated. (**B**) Ventral and sagittal 3D reconstructions of a *dB2-Tomato* brainstem at birth (P0). tdTomato^+^ cells were densely packed in the intertrigeminal (ITR), vestibular (Ves), retrotrapezoid (RTN), and epifacial (epiVII) subgroups (yellow dashed lines). See also movie S1. In addition, four subgroups of tdTomato^+^ cells were seen in the caudal medulla (white dashed lines, see below) and scattered cells (labeled as somaV) in the somatosensory trigeminal nuclei. Because of space limitations, some tdTomato^+^ cells might not be included within the dashed lines. (**C** and **D**) A sagittal *dB2-Tomato* brainstem section stained against the red fluorescent protein (RFP; to detect tdTomato, red) and DAPI (blue) at P0 [in (C)]. The cerebellum (cb), spinal cord (sc), trigeminal (nV), facial (nVII), and ambiguus (NA) motor nuclei are indicated. DAPI signals are removed in (D) for a better visualization of dB2 neuron subgroups. Yellow dashed lines indicate dB2 neuron subgroups that actively coexpress Lbx1 and Phox2b (see text and fig. S3). (**E** to **H**) Transverse *dB2-Tomato* brainstem sections stained against RFP (to detect tdTomato, red)], Lbx1 [blue, false color, (F) and (H)], and Phox2b [green, (F) and (H)] at P0, as indicated in (C). DAPI (blue) and tdTomato only signals are displayed in (E) and (G). The caudal epiNTS, periNA, epiNA, and infraSpV dB2 neuron subgroups are marked in (E) and (G) (see also fig. S4). Arrowheads in (F) (insets) indicate triple-positive (Lbx1^+^/Phox2b^+^/tdTomato^+^) cells. Arrows in (E) and (G) denote scattered somaV cells in the spinal somatosensory trigeminal nucleus (SpV). The area postrema (AP) and nucleus tractus solitarius (nTS), as well as the vagal (nX), hypoglossal (nXII), and ambiguus (NA) motor nuclei are indicated.

To reveal whether additional subgroups of dB2 neurons might exist in the brainstem, whose expression of Lbx1 and/or Phox2b becomes extinguished during their maturation, we next used an intersectional genetic labeling strategy to mark selectively all neurons with a history of Lbx1 and Phox2b expression with a fluorescent reporter. To this end, we used the *RCFL-tdT* allele that expresses cytoplasmic tdTomato after the excision of two stop cassettes flanked by *LoxP* and *FRT* sites. These stop cassettes were excised by Cre and FlpO recombinases driven by *Lbx1* (*Lbx1^Cre^*) and *Phox2b* (*Phox2b^FlpO^*), respectively (Fig. 2A). Brains prepared from *Lbx1^Cre/+^;Phox2b^FlpO/+^;RCFL-tdT^+/−^* (for simplicity *dB2-Tomato*) newborn mice were then processed for light-sheet microscopy. Three-dimensional (3D) reconstructions and immunofluorescence of *dB2-Tomato* brains confirmed the specific location of dB2 neurons to the pons and the medulla oblongata in the eight identified subgroups of Lbx1^+^/Phox2b^+^ neurons (Fig. 2, B to D; figs. S3 and S4; and movie S1). This strategy also uncovered three additional subgroups of dB2 (tdTomato^+^) neurons in the caudal medulla which lose coexpression of Lbx1 or Phox2b by birth (Fig. 2, D to H, and fig. S4). These locate to (i) dorsal to the nucleus tractus solitarius (called here epiNTS), (ii) dorsal to the nucleus ambiguus (called here epiNA), and (iii) underneath the spinal trigeminal nucleus (called here infraSpV) (Fig. 2, D to H, and fig. S4). Of note, our intersectional labeling strategy also identified a number of tdTomato^+^ cells within the somatosensory trigeminal nuclei [called here somaV; Fig. 2, indicated with arrows in (E and G); quantified in fig. S4F]. Since somatosensory trigeminal neurons derive from late dB1 (Phox2b^−^) and dB3 (Phox2b^−^) progenitor cells (*28*, *29*, *31*, *32*), these data indicate that a small proportion of dB2 (Phox2b^+^) progenitor cells is recruited to the pool of progenitor cells that generate somatosensory trigeminal neurons in late development (see Discussion). We conclude that while most dB2 neuron subgroups retain coexpression of Lbx1 and Phox2b in the pons and rostral medulla at birth, most caudally located subgroups of dB2 neurons lose coexpression of either of these factors during their maturation (summarized in fig. S4D).

### dB2 neurons from rhombomeres 5 and 6 are critical for ventilation and the hypercarbic reflex

Intertrigeminal dB2 neurons originate in rhombomere 2 and are developmentally intact in homozygous *Lbx1^FS^* and conditional *Phox2b^Cre/+^;Lbx1^FS/lox^* mice (*28*, *37*). We therefore hypothesized that deficits in dB2 neurons generated from rhombomeres 3 to 6 might account for the respiratory phenotypes observed in the *Lbx1^FS^* animals (*28*). To test this, we used three distinct Cre driver mouse lines, first to lineage trace the origin of distinct subgroups of dB2 neurons: *Egr2^Cre^* (expressed in rhombomeres 3 and 5), *TgHoxb1^Cre^* (expressed in rhombomere 4), and *TgHoxa3^Cre^* (expressed in rhombomeres 5 and 6) (fig. S5). The combination of these Cre lines with the reporter *Rosa^LSL-nGFP^* line revealed the origin of most dB2 neuron subgroups that actively coexpress Lbx1 and Phox2b (fig. S6). However, caudal dB2 neurons of the periNA subgroup, and possibly the other caudal dB2 populations that lose Lbx1 and Phox2b coexpression, were not marked by any of the three Cre driver lines, attributable either to an origin of these neurons outside of rhombomeres 3 to 6 or perhaps to an incomplete expression of the Cre recombinase in rhombomere 6 by the *TgHoxa3^Cre^* mouse line (fig. S6 and below).

**Fig. 3.**
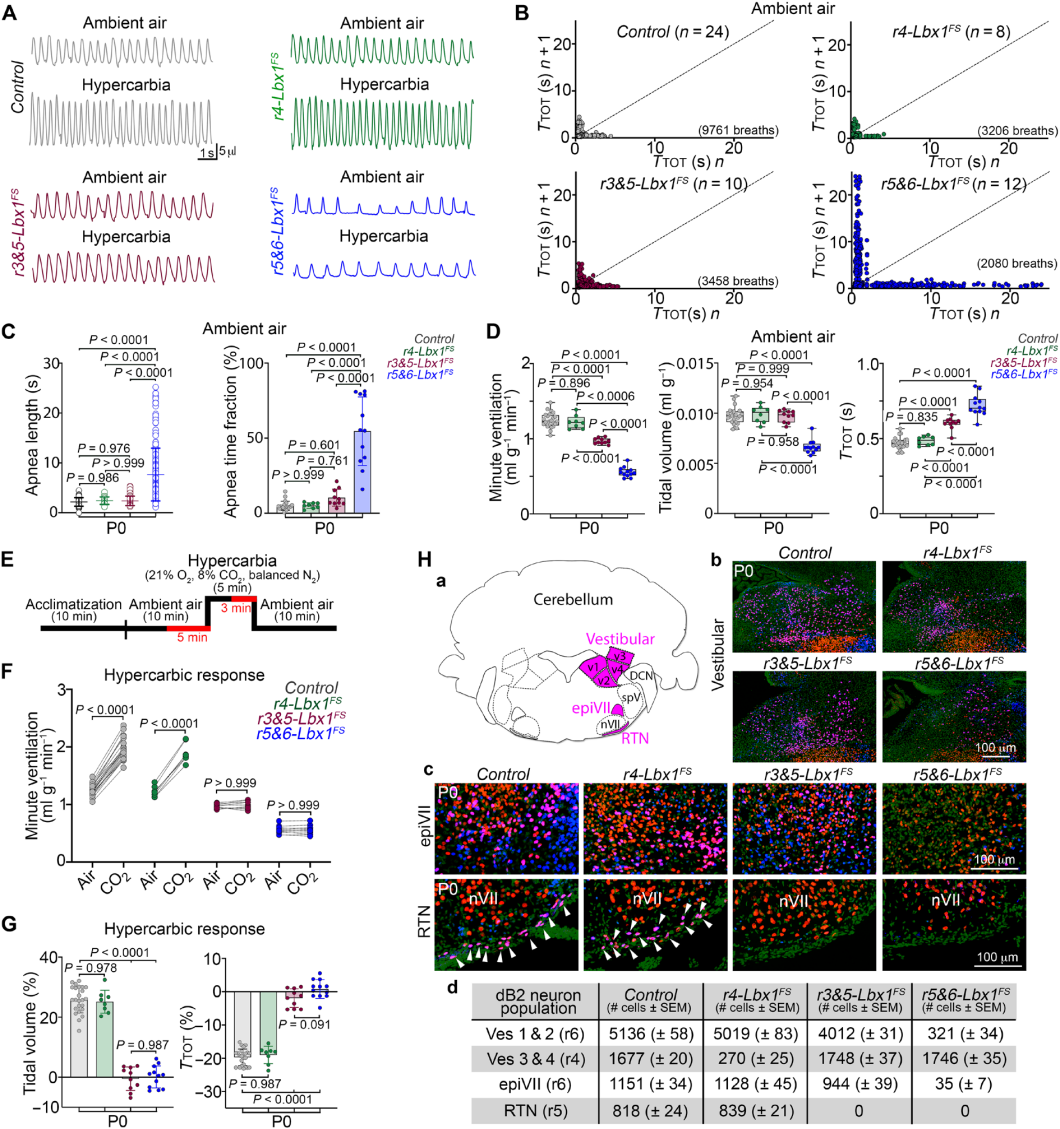
dB2 neurons from rhombomeres 5 and 6 are essential for ventilatory control and the hypercarbic reflex at birth. Plethysmography and anatomical analyses of *Control*, *(Tg)Hoxb1^Cre/+^;Lbx1^FS/lox^* (*r4-Lbx1^FS^*), *Egr2^Cre/+^;Lbx1^FS/lox^* (*r3&5-Lbx1^FS^*), and *(Tg)Hoxa3^Cre/+^;Lbx1^FS/lox^* (*r5&6-Lbx1^FS^*) newborn (P0) mice. (**A**) Plethysmography traces for the indicated genotypes and conditions. (**B**) Poincaré plots of breathing instability for the indicated genotypes in ambient air. For SDs 1 and 2, see fig. S7. Every dot represents individual breaths. Number of breaths and mice (*n*) analyzed are indicated in parentheses. (**C**) Quantification of apnea lengths (left) and the fraction of time in apnea (right) for the indicated genotypes while breathing ambient air. Each circle represents individual apneas (left), while each dot the mean of individual mice (right). (**D**) Quantification of minute ventilation, tidal volumes, and respiratory cycle lengths (*T*_TOT_) for the indicated genotypes while breathing ambient air. (**E**) Diagram illustrating the protocol used to induce a hypercarbic response in newborns. Newborns were analyzed in ambient air and in hypercarbia as indicated (in red). (**F**) Quantification of minute ventilation for the indicated genotypes while breathing ambient air or hypercarbic air. (**G**) Respiratory responses to hypercarbia expressed as percentage of change relative to the baseline (ambient air) for the indicated genotypes. (**H**) Histological characterization of *Control*, *r4-Lbx1^FS^*, *r3&5-Lbx1^FS^*, and *r5&6-Lbx1^FS^* newborns. (a) Schema illustrating the location of vestibular (v1 to v4 subgroups), epifacial (epiVII), and retrotrapezoid (RTN) dB2 neurons. (b and c) Immunofluorescence using antibodies against Lbx1 (blue) and Phox2b (red). DAPI (green, false color) was used to counterstain. (d) Quantification summary of the indicated dB2 neuron subgroups and genotypes, see also fig. S8 [(A) to (D)]. Each dot represents the mean of individual mice in (D), (F), and (G). Significance was determined using one-way analysis of variance (ANOVA) followed by post hoc Tukey’s analysis. Tabulated data can be found in data S2.

We took advantage of these Cre driver lines to restrict differentially the *Lbx1^FS^* mutation to specific rhombomeres by generating *TgHoxb1^Cre/+^;Lbx1^FS/lox^* (for simplicity *r4-Lbx1^FS^*), *Egr2^Cre/+^;Lbx1^FS/lox^* (*r3&5-Lbx1^FS^*), and *TgHoxa3^Cre/+^;Lbx1^FS/lox^* (*r5&6-Lbx1^FS^*) mice. These conditional animals were then analyzed by head-out plethysmography immediately at birth to determine their minute ventilation (amount of air breathed per minute), tidal volumes (amount of air taken per breath), and breathing cycle lengths (abbreviated in the figures as *T*_TOT_). As compared to littermate controls, *r4-Lbx1^FS^* pups displayed no significant differences in their minute ventilation, tidal volumes, nor in their respiratory cycle lengths while breathing ambient air (Fig. 3, A to D). In contrast, *r3&5-Lbx1^FS^* newborns showed a mild hypoventilation phenotype characterized by longer respiratory cycle lengths but no significant change in their tidal volumes (Fig. 3, A to D). Notably, *r5&6-Lbx1^FS^* newborns displayed a severe hypoventilation phenotype that resulted from shallow tidal volumes, aberrantly long respiratory cycle lengths, and frequent apneic events that lasted from 3 to 30 s (Fig. 3, A to D, and fig. S7). One should note that an increase in respiratory cycle length is concomitant with a reduction in breathing frequency. Notably, the respiratory phenotype observed in *r5&6-Lbx1^FS^* newborns closely resembled that reported for homozygous *Lbx1^FS^* and conditional *Phox2b^Cre/+^;Lbx1^FS/lox^* animals (*28*).

In humans and mice, pulmonary ventilation rapidly increases in response to elevated levels of atmospheric CO_2_ (*38*–*40*). We next evaluated whether *r4-Lbx1^FS^*, *r3&5-Lbx1^FS^*, and *r5&6-Lbx1^FS^* newborns fail to respond to a hypercarbic challenge (high CO_2_ in the air: 21% O_2_, 8% CO_2_, balanced N_2_), a respiratory deficit observed in patients presenting with hypoventilation, such as CCHS, and in the homozygous *Lbx1^FS^* and conditional *Phox2b^Cre/+^;Lbx1^FS/lox^* mice (*9*, *10*, *28*, *41*, *42*). Of note, healthy humans and mice respond to hypercarbic challenges by increasing their minute ventilation and tidal volumes while decreasing respiratory cycle length. This demonstrated that *r4-Lbx1^FS^* pups efficiently respond to hypercarbia (Fig. 3, E to G). In contrast, neither *r3&5-Lbx1^FS^* nor *r5&6-Lbx1^FS^* newborns showed a response to the gas exposure (Fig. 3, E to G). Last, we used immunofluorescence to examine the brainstems of *r4-Lbx1^FS^*, *r3&5-Lbx1^FS^*, and *r5&6-Lbx1^FS^* newborns to determine any potential change in Lbx1^+^/Phox2b^+^ dB2 neuron subgroups. This illustrated the unique impairment of lateral vestibular (v3 and v4 subgroups) dB2 neurons in *r4-Lbx1^FS^* pups; the single agenesis of dB2 retrotrapezoid nucleus neurons in *r3&5-Lbx1^FS^* newborns; and the absence of medial vestibular (v1 and v2 subgroups) dB2 neurons, epiVII cells, and dB2 retrotrapezoid nucleus neurons in *r5&6-Lbx1^FS^* animals [Fig. 3H, quantified in fig. S8 (A to D), see also figs. S9 and S10]. One should note that the Lbx1^+^/Phox2b^+^ periNA population was unchanged in each of the three analyzed genotypes (fig. S8E and below), suggesting that other caudal dB2 neuron subgroups might be unaffected in *r4-Lbx1^FS^*, *r3&5-Lbx1^FS^*, and *r5&6-Lbx1^FS^* animals. Taking these data together, we conclude that (i) lateral vestibular dB2 neurons are dispensable for breathing, (ii) agenesis of dB2 retrotrapezoid nucleus neurons in *r3&5-Lbx1^FS^* and in *r5&6-Lbx1^FS^* newborns correlates with prolonged respiratory cycle lengths and the inability to respond to hypercarbia, and (iii) the absence of medial vestibular dB2 neurons and epiVII cells in *r5&6-Lbx1^FS^* newborns correlates with shallow tidal volumes and the appearance of apneic behavior.

**Fig. 4.**
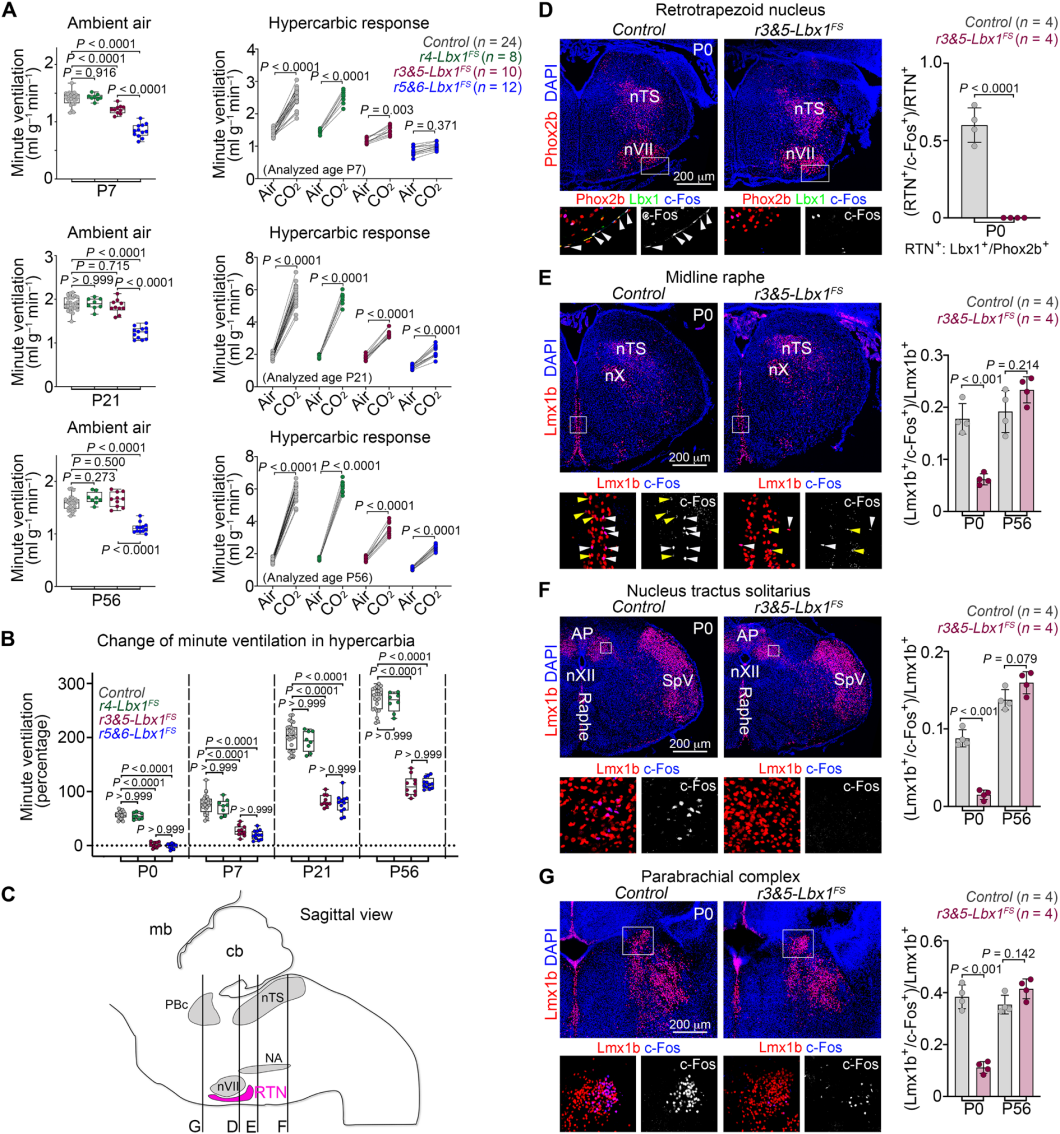
Partial recovery of the hypercarbic reflex in mature *r3&5-Lbx1^FS^* and *r5&6-Lbx1^FS^* mice. (**A**) Quantification of minute ventilation displayed by *Control*, *r4-Lbx1^FS^*, *r3&5-Lbx1^FS^*, and *r5&6-Lbx1^FS^* mice at the indicated stages, in ambient air (left) or high levels of CO_2_ (hypercarbia) (right), see also fig. S11. The number of mice (*n*) analyzed is in parentheses. (**B**) Respiratory response to hypercarbia expressed as percentage of change relative to the baseline (ambient air). Change of minute ventilation displayed by the indicated genotypes at different postnatal ages: P0, P7, P21, and P56. See also fig. S11B. (**C**) Schematic view of a mouse brainstem displaying the location of the transverse planes shown in (D) to (G). (**D** to **G**) Histological detection of c-Fos^+^ cells after hypercarbia exposure in *Control* and *r3&5-Lbx1^FS^* mice at P0. DAPI (blue) was used to counterstain (main). Retrotrapezoid nucleus neurons were detected with Lbx1 (green) and Phox2b (red) antibodies [in (D)]. Raphe [in (E)], nucleus tractus solitarius [in (F)], and parabrachial complex [in (G)] neurons were detected with Lmx1b (red) antibodies. Boxed areas in the main photographs are magnified at the bottom with merged or c-Fos only signals. White and yellow arrowheads in (E) denote neurons with strong and weak c-Fos immunoreactivity, respectively. See fig. S11C for the histological analysis of *r3&5-Lbx1^FS^* mice at P56*.* The nucleus tractus solitarius (nTS) and area postrema (AP), as well as the facial (nVII), vagal (nX), and hypoglossal (nXII) motor nuclei are indicated for orientation. Right: Quantification of the proportion of retrotrapezoid nucleus, nucleus tractus solitarius, middle raphe, and parabrachial complex neurons coexpressing c-Fos at the indicated stages. Each dot represents the mean of individual mice. Significance was determined using one-way ANOVA followed by post hoc Tukey’s analysis. Tabulated data can be found in data S3.

Unlike homozygous *Lbx1^FS^* and conditional *Phox2b^Cre/+^;Lbx1^FS/lox^* animals (*28*), all conditional *r4-Lbx1^FS^*, *r3&5-Lbx1^FS^*, and *r5&6-Lbx1^FS^* pups survive the perinatal period. This allowed us to analyze their breathing behavior at later postnatal stages: P7 (neonate), P21 (juvenile), and P56 (adult). As in the newborn period, *r4-Lbx1^FS^* mice did not display any obvious impairment either in ventilation or in response to hypercarbia at any of the analyzed postnatal stages (Fig. 4A and fig. S11, A and B). This confirmed that dB2 neuron subgroups derived from rhombomere 4 are dispensable for breathing. Plethysmographic recordings of *r3&5-Lbx1^FS^* animals still showed a mild hypoventilation at P7 but no ventilatory deficit at more mature (P21 or P56) stages while breathing ambient air (Fig. 4A and fig. S11A). In contrast, *r5&6-Lbx1^FS^* mice significantly hypoventilate throughout postnatal life in ambient air (Fig. 4A). This deficit was mainly due to shallow tidal volumes in these animals (fig. S11A). Respiratory cycle lengths were only partially increased in both *r3&5-Lbx1^FS^* and *r5&6-Lbx1^FS^* neonates but did not differ from control animals at more mature stages (fig. S11A). Furthermore, the incidence of apneic events and pronounced respiratory instability observed in *r5&6-Lbx1^FS^* newborn mice was still seen in the neonatal period but not in later stages (figs. S12 and S13). Together, these data show that the developmental elimination of medial vestibular (v1 and v2) dB2 neurons and epiVII cells (in *r5&6-Lbx1^FS^* mice) correlates with shallow tidal volumes throughout postnatal life and respiratory instability in neonates, while the absence of dB2 retrotrapezoid nucleus neurons (in *r3&5-Lbx1^FS^* and *r5&6-Lbx1^FS^* mice) affects respiratory cycle lengths specifically in neonates.

Next, we analyzed the ventilatory responses of *r3&5-Lbx1^FS^* and *r5&6-Lbx1^FS^* animals to hypercarbia at P7, P21, and P56. In contrast to the lack of response to the gas challenge seen in both genotypes at birth, we detected a progressive increase in their ventilatory responses to hypercarbia from the neonatal stage (P7) to the adulthood (P56) (Fig. 4, A and B, and fig. S11B). One should note, however, that the maximal response to hypercarbia seen in adult *r3&5-Lbx1^FS^* and *r5&6-Lbx1^FS^* animals represented only one third of that observed in control mice of the same age (Fig. 4B). Thus, dB2 retrotrapezoid nucleus neurons are crucial for the hypercarbic response during the neonatal life, but the response to hypercarbia appears to result from the cooperation of dB2 retrotrapezoid nucleus neurons with additional chemoreceptor neurons as the animal matures.

To understand how the absence of dB2 retrotrapezoid nucleus neurons affects the central circuit mediating the hypercarbic reflex, we stained brainstem sections taken from newborn and adult *r3&5-Lbx1^FS^* mice, after an hour-long exposure to hypercarbia, with antibodies against c-Fos (Fig. 4, C to G). This protocol allows for the labeling of neurons activated by the hypercarbic challenge. We analyzed *r3&5-Lbx1^FS^* animals for this experiment as their unique recognizable deficit is the lack of dB2 retrotrapezoid nucleus neurons. As expected, c-Fos^+^ cells were undetected in the “retrotrapezoid area” of *r3&5-Lbx1^FS^* newborn mice (Fig. 4D). Unexpectedly, we hardly detected c-Fos^+^ cells in other regions known to participate in the hypercarbic reflex (*40*), such as the midline medullary raphe (that is, raphe obscurus, magnus, and pallidus), the caudal part of the nucleus tractus solitarius, or the parabrachial complex in *r3&5-Lbx1^FS^* newborns (Fig. 4, E to G). In contrast, no difference in the number of c-Fos^+^ cells was observed in the midline raphe, nucleus tractus solitarius, and parabrachial complex in mature *r3&5-Lbx1^FS^* animals when compared to controls of the same age [fig. S11C, quantified in Fig. 4 (E to G)]. We conclude that dB2 retrotrapezoid nucleus neurons are a prerequisite for the activation of the central circuit mediating hypercarbia at birth, but some elements of this circuit can be activated in the absence of dB2 retrotrapezoid nucleus neurons in adult life.

### Agenesis of caudal dB2 neurons is associated with perinatal lethality

Despite the close phenotypic resemblance of *r5&6-Lbx1^FS^* newborns to homozygous *Lbx1^FS^* and conditional *Phox2b^Cre/+^;Lbx1^FS/lox^* pups in terms of hypoventilation patterns, shallow tidal volumes, and frequent apneic behavior, no *r5&6-Lbx1^FS^* animal died at birth. We then asked whether periNA cells, as well as the other previously unidentified caudal dB2 subgroups (epiNA, epiNTS, and infraSpV), were affected in *Lbx1^FS^* mutant mice. To this end, we extended the use of our intersectional *dB2-Tomato* line to carry the *Lbx1^FS^* mutation and determined changes in these caudal dB2 neurons by immunofluorescence. This showed that epiNA, epiNTS, and infraSpV dB2 caudal subgroups were misspecified and underwent marked fate shifts in *Lbx1^Cre/FS^;Phox2b^Flpo/+^;RCFL-tdT^+/−^* (for simplicity, *dB2-Tomato-Lbx1^FS^*) mice (fig. S14). For instance, cells of the epiNTS subgroup were converted into Phox2b^+^ neurons of the nucleus tractus solitarius, whereas the periNA, epiNA, and infraSpV subgroups appeared to be mislocated to the spinal somatosensory trigeminal nucleus (fig. S14). Notably, while dB2 neurons, such as retrotrapezoid nucleus, periNA, epiNA, epiNTS, and infraSpV, were absent in *dB2-Tomato-Lbx1^FS^* mice, somaV neurons appeared unchanged in these animals (arrowheads in fig. S14A), supporting the notion that these neurons might not belong to the dB2 lineage but instead are dB1 or dB3 derivatives (see Discussion). We conclude that agenesis of caudal dB2 neurons in homozygous *Lbx1^FS^* newborns might cause their lethality.

**Fig. 5.**
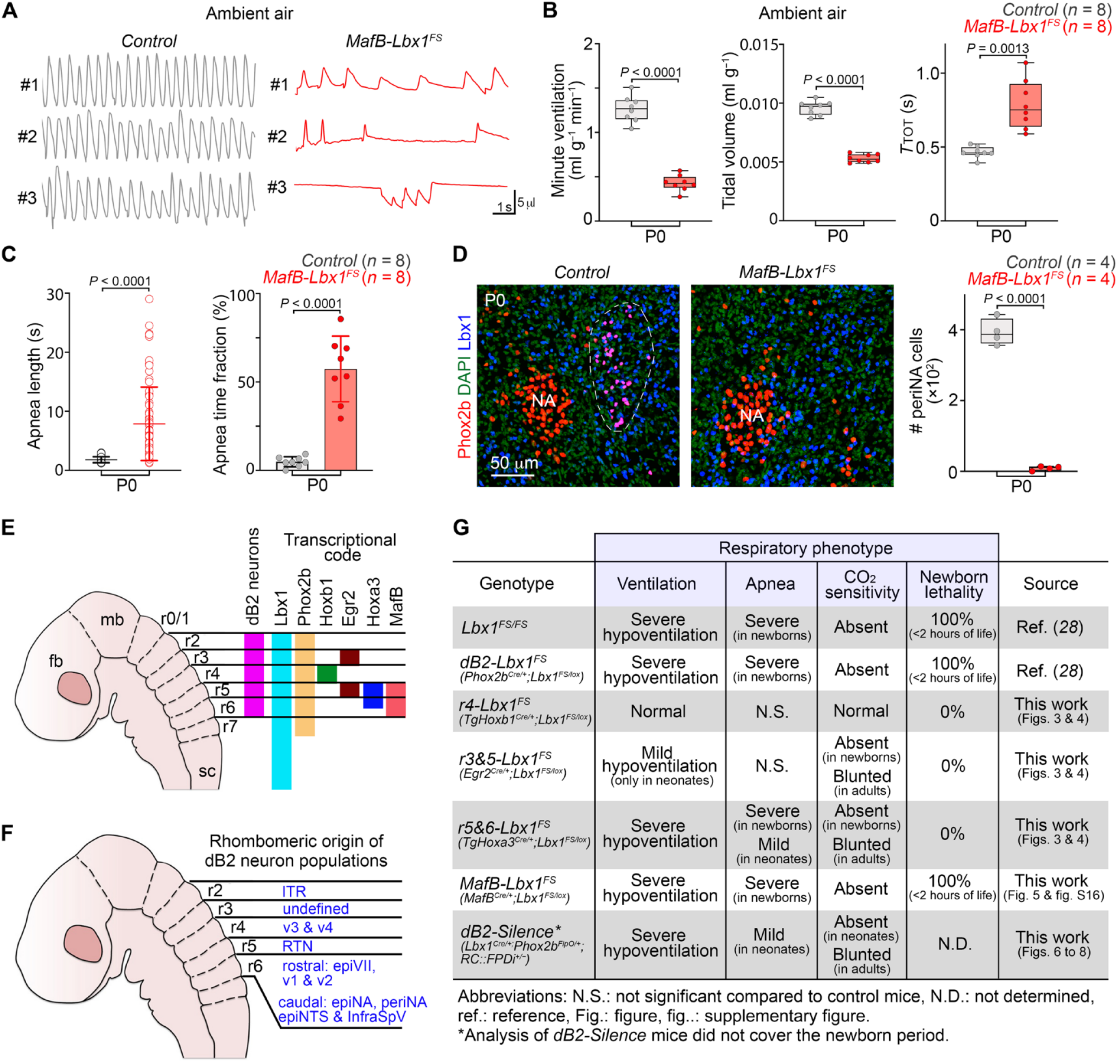
Agenesis of caudal dB2 neurons causes respiratory arrest at birth. (**A**) Plethysmography traces of three individual *Control* and three *MafB^Cre/+^;Lbx1^FS/lox^* (*MafB-Lbx1^FS^*) newborn (P0) mice while breathing ambient air. (**B**) Quantification of minute ventilation, tidal volume, and respiratory cycle lengths (*T*_TOT_) in *Control* and *MafB-Lbx1^FS^* newborns in ambient air. Each dot represents the mean of individual mice. (**C**) Quantification of apnea lengths (left) and the fraction of time in apnea (right) of *Control* and *MafB-Lbx1^FS^* newborn mice while breathing ambient air. Each circle represents individual apneas (left), while each dot the mean of individual mice (right). (**D**) Histological analysis and quantification of peri nucleus ambiguus (periNA) neurons in *Control* and *MafB-Lbx1^FS^* newborns at birth (P0). The transverse brainstem sections were stained with Lbx1 (blue) and Phox2b (red) antibodies. DAPI (green, false color) was used to counterstain. The number (*n*) of mice analyzed is indicated in parentheses. See fig. S16 for the analysis of other caudal dB2 neuron subgroups in *MafB-Lbx1^FS^* mutants. (**E**) Schema displaying the rhombomeric (r) segmentation of the developing brainstem, the origin of dB2 neurons (magenta), and the expression patterns of the genes analyzed in this study. The forebrain (fb), midbrain (mb), and spinal cord (sc) are indicated for anatomical orientation. (**F**) Schema summarizing the rhombomeric origin of the identified dB2 neuron subgroups in this study. Note that somaV neurons, which are generated from each rhombomere 2 to 6 are not marked (see Discussion). (**G**) Summary of the main findings of this study. The phenotypes of the previously reported homozygous *Lbx1^FS^* (*28*), conditional *Phox2b^Cre/+^;Lbx1^FS/lox^* (*28*) are also displayed for comparison. Two-tailed *t* tests were performed to determine statistical significance in (B) to (D). Tabulated data can be found in data S4.

We next compared the recombination pattern of the *TgHoxa3^Cre^* line with a second Cre driver line that has also been reported to recombine rhombomeres 5 and 6 during early development, *MafB^Cre^* (*43*). Specifically, we compared the recombination patterns of these two Cre lines at embryonic day 11.5 (E11.5), the developmental time point when dB2 neurons are specified (*28*, *33*). To map accurately the borders of these rhombomeres, we also incorporated the *Egr2^Cre/+^* driver line into our analysis. Cre recombination was visualized with the *Rosa^LSL-nGFP^* reporter. This revealed that *MafB^Cre^*, but not *TgHoxa3^Cre^*, recombines the full extension of rhombomere 6 (fig. S15). Next, we used the *MafB^Cre^* driver line to restrict the *Lbx1^FS^* mutation to rhombomeres 5 and 6 and generated *MafB^Cre/+;^Lbx1^FS/lox^* (for simplicity, *MafB-Lbx1^FS^*) animals. Four of 12 *MafB-Lbx1^FS^* newborns immediately died at birth without any apparent breathing behavior. The remaining (8 of 12) rarely displayed a continuous breathing pattern but instead exhibited notable gasping behavior with prolonged apneic times and survived for a maximum of 2 hours after delivery (Fig. 5, A to C). Furthermore, *MafB-Lbx1^FS^* newborns did not mount a hypercarbic response (fig. S16, A and B). Histological examination of *MafB-Lbx1^FS^* pups using Lbx1 and Phox2b antibodies revealed the absence of a recognizable periNA subgroup, in addition to the lack of medial vestibular dB2 neurons, epiVII cells, and dB2 retrotrapezoid nucleus neurons (Fig. 5D and fig. S16, C and D). We conclude that dB2 neurons originating from rhombomere 6 are required for ventilatory control, correct tidal volumes, and neonatal survival [summarized in Fig. 5 (E to G)].

### dB2 neurons are cell-autonomously required for neonatal ventilation and the hypercarbic reflex

**Fig. 6.**
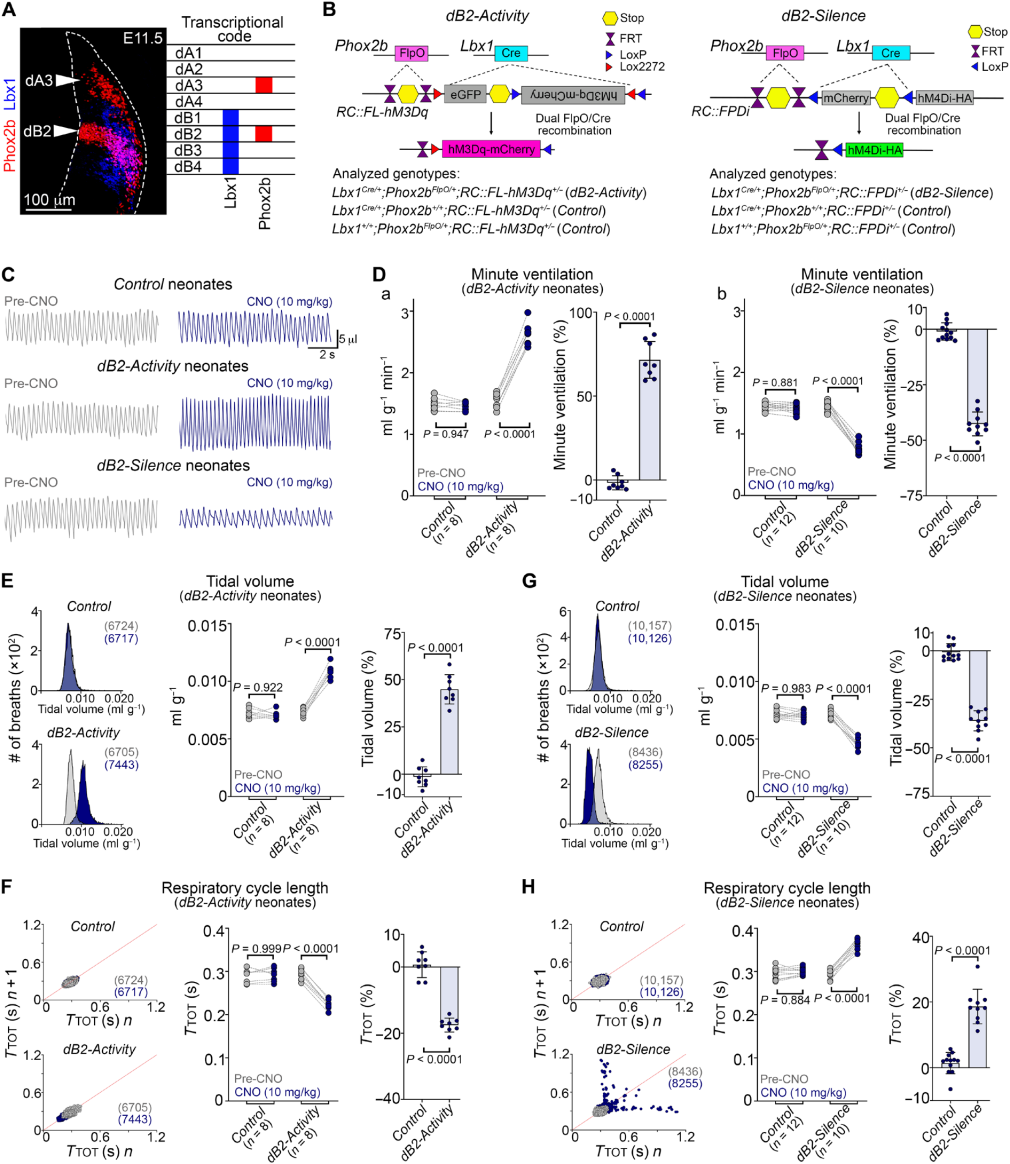
Ventilatory changes caused by the activation or inhibition of dB2 neurons in neonatal mice. (**A**) Left: A transverse brainstem section stained with Phox2b (red) and Lbx1 (blue) antibodies at E11.5. Right: Gene expression in brainstem neurons at E11.5. (**B**) Strategies to express hM3Dq-mCherry and hM4Di-HA DREADD receptors in dB2 neurons. Analyzed genotypes are indicated. See also fig. S17. (**C** to **H**) Analysis of *Control*, *dB2-Activity*, and *dB2-Silence* neonates before (pre; gray) and after CNO treatment (10 mg/kg; blue). Respiratory recordings were taken in ambient air. The number (*n*) of mice analyzed is displayed in parentheses underneath the studied genotypes. (C) Plethysmography traces of *Control*, *dB2-Activity*, and *dB2-Silence* neonates. (D) Left in (a) and (b): Quantification of minute ventilation. Right in (a) and (b): Change of minute ventilation expressed as percentage relative to the baseline (before CNO). [(E) and (G)] Left: Frequency distribution plots of tidal volumes. Total number of analyzed breaths is indicated in parentheses. Note that a displacement to the left or right indicates a decrease or increase, respectively. Middle: Quantification of tidal volumes. Right: Change of tidal volumes expressed as percentage relative to the baseline (before CNO). [(F) and (H)] Left: Poincaré plots illustrating breathing instability. Every dot represents individual breaths, and the total number of analyzed breaths is indicated in parentheses. SDs 1 and 2 are displayed in fig. S22. Middle: Quantification of respiratory cycle lengths (*T*_TOT_). Right: Change of *T*_TOT_ expressed as percentage relative to the baseline (before CNO). Except for the Poincaré plots, every dot in graphs (D) to (H) represents the mean of individual mice. Significance was determined using one-way ANOVA followed by post hoc Tukey’s analysis for group comparison or two tailed *t* test for pair comparison. Tabulated data can be found in data S5.

Although the variety of breathing deficiencies observed in *r3&5-Lbx1^FS^*, *r5&6-Lbx1^FS^*, and *MafB-Lbx1^FS^* mice correlates with the specific agenesis of distinct subgroups of dB2 neurons generated from rhombomeres 5 and 6, there exists the possibility that those dB2 subgroups are not directly implicated in respiration but important for the development of other interconnected neurons which could be the actual contributors to the described respiratory phenotypes for these conditional *Lbx1^FS^* mice. To determine whether dB2 neurons have a direct function in breathing, we used two different murine intersectional chemogenetic strategies to drive (hM3Dq) or silence (hM4Di) their neural activity in neonatal (P7), juvenile (P21) and adult (P56) mice in a transient and reversible manner. We excluded the analysis of these intersectional chemogenetic mice at birth (P0) to prevent a potential lethal phenotype that could result from the activation or inhibition of dB2 neurons. Specifically, we used the *RC::FL-hM3Dq* (*44*) and *RC::FPDi* (*45*) alleles that express an mCherry-tagged hM3Dq or an hemagglutinin (HA)–tagged hM4Di designer receptor exclusively activated by designer drugs (DREADD) receptor, respectively, upon dual Cre/*FlpO*-mediated recombination of *Lox*- and *FRT-*flanked STOP cassettes. As with the *RCFL-tdT* allele, these stop cassettes were recombined by *Lbx1^Cre^* and *Phox2b^FlpO^* (Fig. 6, A and B).

We first verified the correct expression of the fluorescent (mCherry-tagged) hM3Dq receptor in the distinct subgroups of dB2 neurons by staining brainstem sections taken from *Lbx1^Cre/+^;Phox2b^FlpO/+^;RC::FL-hM3Dq^+/−^* (for simplicity, dB2-Activity) mice with antibodies against the red fluorescent protein (RFP; to detect mCherry; figs. S17 and S18). Similarly, we also confirmed that dB2 neuron subgroups expressed the nonfluorescent HA-tagged hM4Di receptor in *Lbx1^Cre/+^;Phox2b^FlpO/+^;RC::FPDi^+/−^* (for simplicity, dB2-Silence) mice with anti-HA antibodies (figs. S17 and S18). Next, we assessed the response of DREADD-expressing dB2 neurons to the synthetic DREADD agonist clozapine N-oxide (CNO) in neonates (P5 to P7) and more mature (P21 to P56) mice with slice physiology. To this end, we used acute slices taken from the medulla of *dB2-Activity* mice, whose DREADD-expressing dB2 neurons can be visualized by mCherry. In both age groups, dB2 neurons showed robust and comparable depolarization upon CNO application, as well as an increase in spike fidelity, indicating that DREADD expression levels in neonatal dB2 neurons are already high enough to allow for an efficient chemogenetic modulation of their neuronal activity (fig. S19). Next, we compared the respiration of *dB2-Activity* and *dB2-Silence* mice with that of four distinct age-matched control littermate genotypes: (i) *Lbx1^+/+^;Phox2b^FlpO/+^;RC::FL-hM3Dq^+/−^*, (ii) *Lbx1^Cre/+^;Phox2b^+/+^;RC::FL-hM3Dq^+/−^*, (iii) *Lbx1^+/+^;Phox2b^FlpO/+^;RC::FPDi^+/−^*, and (iv) *Lbx1^Cre/+^;Phox2b^+/+^;RC::FPDi^+/−^* (described in Fig. 6B). In the absence of CNO, no differences in minute ventilation, tidal volumes, or breathing cycle lengths were observed between control, *dB2-Activity*, and *dB2-Silence* mice at any of the analyzed stages (fig. S20). However, when the same mice were treated with CNO (10 mg/kg), only *dB2-Activity* and *dB2-Silence* mice displayed pronounced ventilatory changes at all analyzed stages (see fig. S21 and below).

In the neonatal period, CNO treatment of *dB2-Activity* (CNO-*dB2-Activity*) mice greatly raised their minute ventilation by 70% (Fig. 6, C and D). This augmented minute ventilation resulted from a pronounced increase in their tidal volumes (Fig. 6E) and a reduction in their respiratory cycle lengths (Fig. 6F). In contrast, inhibition of dB2 neurons in CNO-treated *dB2-Silence* (CNO-*dB2-Silence*) mice deeply depressed their minute ventilation by 40% (Fig. 6, C and D). The reduced ventilation observed in CNO-*dB2-Silence* mice resulted from noticeable shallow tidal volumes (Fig. 6G) and an increase in their respiratory cycle lengths (Fig. 6H). CNO-*dB2-Silence* neonates also displayed an apparent respiratory instability that was accompanied by spontaneous interruptions of breathing that ranged between 0.5 and 1.2 s in length (Fig. 6H and fig. S22), a phenotype that resembled the respiratory instability observed in *r5&6-Lbx1^FS^* neonates.

**Fig. 7.**
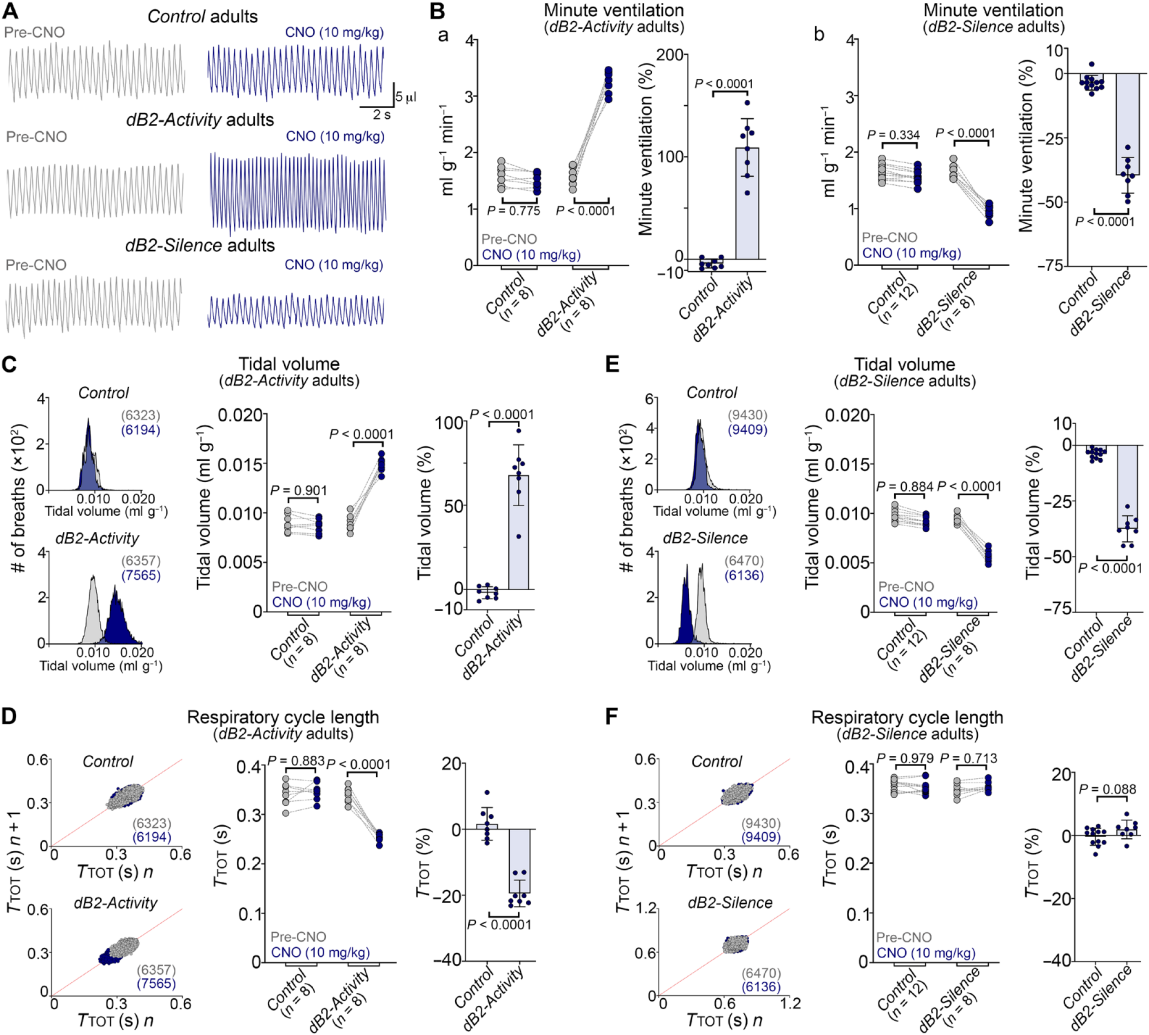
Ventilatory changes caused by the activation or inhibition of dB2 neurons in adult mice. Analysis of *Control*, *dB2-Activity*, and *dB2-Silence* adult mice before (pre; gray) and after CNO treatment (10 mg/kg; blue). Respiratory recordings were taken in ambient air. The number (*n*) of mice analyzed is displayed in parentheses underneath the studied genotypes. (**A**) Plethysmography traces of *Control*, *dB2-Activity*, and *dB2-Silence* mice. (**B**) Left in (a) and (b): Quantification of minute ventilation. Right in (a) and (b): Change of minute ventilation expressed as percentage relative to the baseline (before CNO). (**C** and **E**) Left: Frequency distribution plots of tidal volumes. Total number of analyzed breaths is indicated in parentheses. Note that a displacement to the left or right indicates a decrease or increase, respectively. Middle: Quantification of tidal volumes. Right: Change of tidal volumes expressed as percentage relative to the baseline (before CNO). (**D** and **F**) Left: Poincaré plots illustrating breathing instability. Every dot represents individual breaths, and the total number of analyzed breaths is indicated in parentheses. SDs 1 and 2 are displayed in fig. S24. Middle: Quantification of respiratory cycle lengths (*T*_TOT_). Right: Change of *T*_TOT_ expressed as percentage relative to the baseline (ambient air before CNO). Except for the Poincaré plots, every dot in graphs (B) to (F) represents the mean of individual mice. Significance was determined using one-way ANOVA followed by post hoc Tukey’s analysis for group comparison or two tailed *t* test for pair comparison. Tabulated data can be found in data S6.

In juvenile and adult mice, the activation of dB2 neurons in CNO-*dB2-Activity* mice also led to a marked increase in their minute ventilation by 90 and 110%, respectively (Fig. 7, A and B, and fig. S23A), which resulted from a pronounced increase in their tidal volumes (Fig. 7C and fig. S23A) and a reduction in their respiratory cycle lengths (Fig. 7D and fig. S23A). In contrast, inhibition of dB2 neurons in juvenile and adult CNO-*dB2-Silence* mice markedly reduced their minute ventilation by 40% at both stages (Fig. 7, A and B, and fig. S23B). This reduced minute ventilation resulted from a conspicuous depression in tidal volumes (Fig. 7E and fig. S23B), recapitulating the phenotypes seen in juvenile and adult *r5&6-Lbx1^FS^* mice. Similar to *r3&5-Lbx1^FS^* and *r5&6-Lbx1^FS^* mature mice, no change in respiratory cycle lengths was observed in mature CNO-*dB2-Silence* animals (Fig. 7F and figs. S23B and S24). Taking these data together, we conclude that the respiratory phenotypes seen in *r3&5-Lbx1^FS^* and *r5&6-Lbx1^FS^* mice are caused by the cell-autonomous dysfunction of dB2 neurons, particularly those emanating from rhombomere 5 and rostral rhombomere 6.

**Fig. 8.**
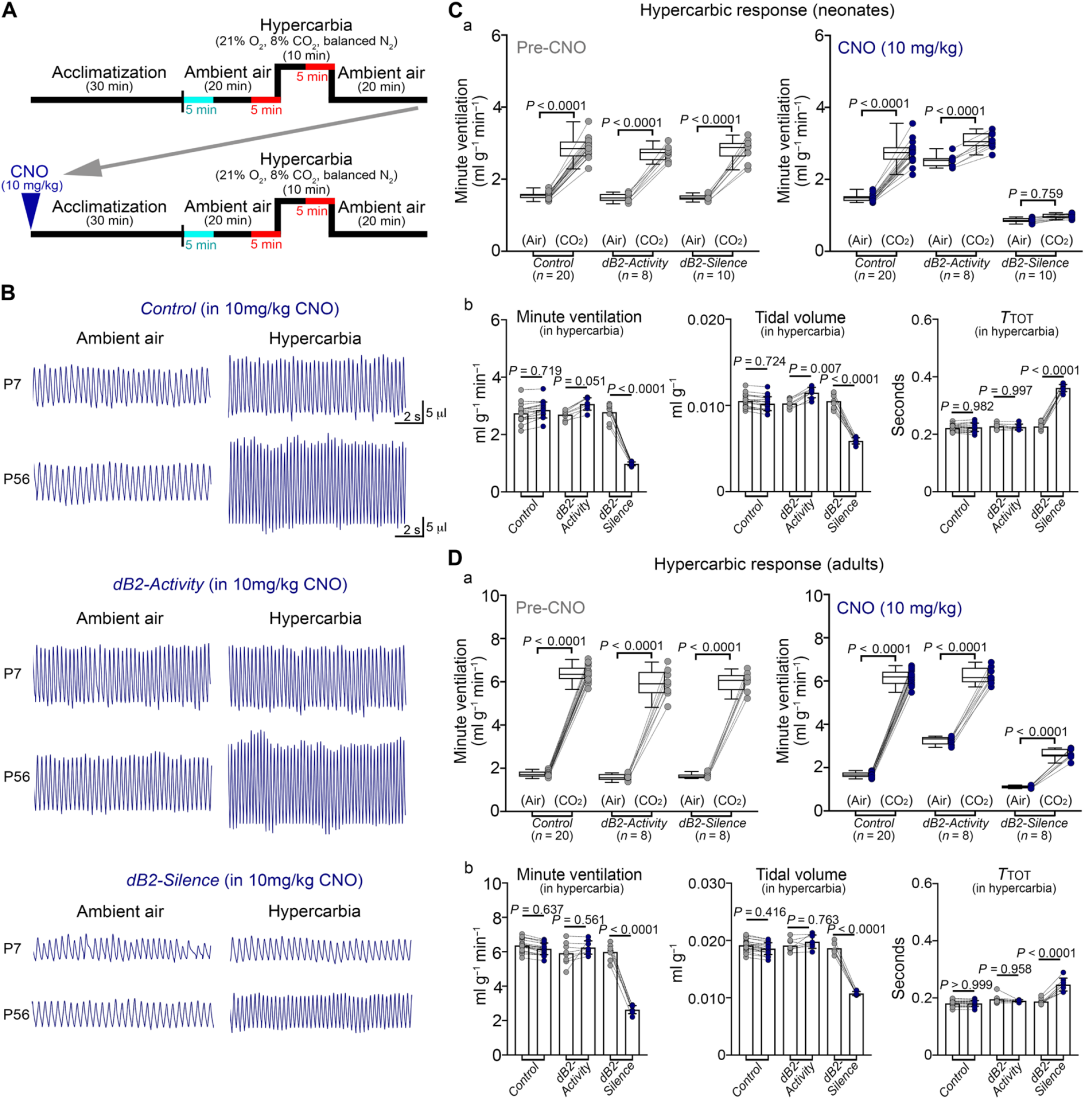
dB2 neurons are essential for the neonatal hypercarbic reflex. (**A**) Diagram illustrating the protocol used to induce a hypercarbic response in mice. Respiration was analyzed in ambient air and in hypercarbia for 5 min (indicated in red). The analysis displayed in Figs. 6 and 7 was obtained from the five minutes denoted in cyan. (**B** to **D**) Analysis of *Control*, *dB2-Activity*, and *dB2-Silence* mice before (pre; gray) and after CNO treatment (10 mg/kg; blue). The number (*n*) of mice analyzed is displayed underneath the studied genotypes. (B) Plethysmography traces of *Control*, *dB2-Activity*, and *dB2-Silence* mice at the indicated stages and conditions. [(C) and (D)] Analysis of *Control*, *dB2-Activity*, and *dB2-Silence* mice in ambient air (air) and in hypercarbia (8% CO_2_ in air, abbreviated as CO_2_). (a) Quantification of minute ventilation in ambient air and hypercarbia for the indicated genotypes, stages, and conditions. (b) Effects of dB2 neuron activation or inhibition on minute ventilation, tidal volumes, and respiratory cycle lengths (*T*_TOT_) displayed by *dB2-Activity* or *dB2-Silence* mice, respectively, while in hypercarbia (before and after CNO treatment). For quantification of tidal volumes and respiratory cycle lengths, see fig. S25. Every dot represents the mean of individual mice. Significance was determined using one-way ANOVA followed by post hoc Tukey’s analysis for group comparison or two tailed *t* test for pair comparison. Tabulated data can be found in data S7.

We next evaluated the respiratory response of control, *dB2-Activity* and *dB2-Silence* neonates and adult mice to hypercarbia. In the absence of CNO, all mice responded equally to the hypercarbic challenge at both analyzed stages (Fig. 8 and fig. S25). In contrast, CNO-*dB2-Silence* neonates failed to respond to the hypercarbic challenge, while CNO-*dB2-Activity* neonates, which display an increased baseline minute ventilation, mildly increased their response to hypercarbia (Fig. 8C and fig. S25). In keeping with the findings observed in *r3&5-Lbx1^FS^* and *r5&6-Lbx1^FS^* juvenile and adult mice, CNO-*dB2-Silence* adult mice exhibited a severely blunted response to hypercarbia (Fig. 8D and fig. S25). In contrast, the activation of dB2 neurons did not preclude the capacity of CNO-*dB2-Activity* adult mice to respond to the hypercarbic challenge (Fig. 8D and fig. S25). Thus, inhibition of dB2 neuron activity precludes the onset of the hypercarbic response in the neonatal period and severely blunts it in more mature mice. We conclude that dB2 neurons have a strong effect on ventilatory control in mice, and their silencing recapitulates the hypoventilation and hypercarbic phenotypes observed in patients with *LBX1^FS^* and *Lbx1^FS^* mice.

Last, we asked whether dB2 neurons might participate in the chemoreflex to hypoxia (low O_2_ in the air; 10% O_2_, balanced N_2_). To this end, we used CNO-*dB2-Silence* mice, as they both recapitulate the phenotypes observed in *r5&6-Lbx1^FS^* mice and include every other dB2 neuron subgroup. Despite the evident hypoventilation displayed by CNO-*dB2-Silence* mice in ambient air, no differences were observed in their ventilatory response to hypoxia when compared to CNO-treated control animals neither in neonatal nor in adult life (fig. S26). Hence, dB2 neurons are dispensable for the hypoxic reflex. Together, we conclude that dB2 neurons are key in the control of ventilation and specific for the regulation of the hypercarbic chemoreflex.

## DISCUSSION

In this study, we investigated the function of medullary dB2 neurons in neonatal respiratory physiology. Through our intersectional studies, lineage tracing analyses, and use of conditional mutant models, we identify a single subgroup of dB2 neurons (generated from rhombomere 5), as an obligatory element for the central circuit regulating the neonatal hypercarbic reflex and neonatal respiratory frequencies. These experiments also uncovered other components of the neural circuit serving in ventilatory control (generated from rhombomere 6), whose nullification results in various respiratory phenotypes that range from (i) hypoventilation due to shallow tidal volumes, (ii) neonatal respiratory instability, and iii) neonatal respiratory arrest.

In previous work, we identified a recessive mutation in *LBX1* (*LBX1^FS^*) that causes a severe hypoventilation phenotype that resembles CCHS (*28*). One should note that unlike *PHOX2B* mutations, the *LBX1^FS^* variant does not lead to autonomous nervous system anomalies frequently seen in classic CCHS. Mice bearing an analogous mutation in *Lbx1* (*Lbx1^FS^*) die immediately at birth from an apparent failure to breathe and present with a unique deficit in the development of dB2 neurons [(*28*) and this work]. The identification of the *LBX1^FS^/Lbx1^FS^* mutation has thus been key to start dissecting a dysfunctional circuit associated with congenital hypoventilation, as it shows specificity to a unique group of neurons, that is dB2, thereby limiting functional and anatomical explorations to the medulla. The immediate death of homozygous *Lbx1^FS^* newborn mice had however hampered the physiological association of dB2 neurons with the breathing phenotype. In this study, we now produce new murine models for the specific study of dB2 neuron activity and report that their selective impairment results in severe hypoventilation, diminished tidal volumes, slow respiratory frequencies, and an anomalous hypercarbic reflex, which collectively produce a respiratory phenotype that resembles CCHS in neonatal mice. Our findings are of both clinical and biological relevance. From a clinical perspective, this work establishes dB2 neuron dysfunction to be causative of congenital hypoventilation. The specificity of respiratory dysfunctions seen in our new murine models provides an invaluable resource for future investigation and development of therapeutical strategies for the management of congenital hypoventilation diseases, without the confounding factor of autonomous nervous system dysregulation seen in other models, such as mice carrying *PHOX2B* mutations. From the biological standpoint, our work uncovers previously undescribed components of the respiratory circuit regulating homeostatic breathing.

Here, we used intersectional chemogenetics to determine whether the identified respiratory phenotypes seen in our conditional *r3&5-Lbx1^FS^* and *r5&6-Lbx1^FS^* mice result from the specific agenesis of dB2 neurons emanating from rhombomeres 5 and 6. Our data show that inhibition of dB2 neuron activity in CNO-*dB2-Silence* mice abrogates the hypercarbic chemoreflex in neonates and severely blunts it in juvenile and adult mice, a phenocopy of the changes seen in *r3&5-Lbx1^FS^* and *r5&6-Lbx1^FS^* mice in postnatal life. The dB2 neuron subgroup responsible for this is the retrotrapezoid nucleus. *r3&5-Lbx1^FS^* and *r5&6-Lbx1^FS^* newborns, which lack dB2 retrotrapezoid nucleus neurons, are fully insensitive to hypercarbia at birth. The retrotrapezoid nucleus has long been considered a major node for sensing changes of PCO_2_ within the central nervous system (*38*–*40*, *46*, *47*). Here, we show that the agenesis of dB2 retrotrapezoid nucleus neurons precludes the activation of other neurons associated with the hypercarbic reflex, such as the midline raphe (raphe obscurus, magnus, and pallidus), the nucleus tractus solitarius, and the parabrachial complex, at birth. Given that retrotrapezoid nucleus neurons form reciprocal connections with these brainstem centers (*39*, *48*), our data indicate that retrotrapezoid nucleus neurons are not only a central node but are obligatory for the efficient activation of the respiratory circuit driving the response to hypercarbia in newborn mice. Although severely blunted, a significant hypercarbic response was detected in juvenile and adult *r3&5-Lbx1^FS^* and *r5&6-Lbx1^FS^* animals. In keeping with this, we observed activation of midline raphe, nucleus tractus solitarius, and parabrachial cells in mature *r3&5-Lbx1^FS^* animals following a hypercarbic challenge. These data may imply that raphe, nucleus tractus solitarius, and parabrachial cells can be activated independently of the retrotrapezoid nucleus to modulate part of the hypercarbic reflex in adult *r3&5-Lbx1^FS^* and *r5&6-Lbx1^FS^* animals, possibly via carotid body input (*49*, *50*).

We also show here that the agenesis of dB2 retrotrapezoid nucleus neurons, in *r3&5-Lbx1^FS^* and *r5&6-Lbx1^FS^* mice, results in increased baseline respiratory cycle lengths only in the neonatal period, a phenotype recapitulated in CNO-*dB2-Silence* mice (see below). This indicates that as yet unknown physiological mechanisms in *r3&5-Lbx1^FS^* mice compensate for their lack of retrotrapezoid nucleus neurons to maintain baseline respiratory frequencies after the neonatal period. In this context, neither juvenile nor adult *r3&5-Lbx1^FS^* mice, whose only recognizable deficit is the absence of dB2 retrotrapezoid nucleus neurons, display any significant differences in baseline tidal volumes nor in respiratory cycle lengths when compared to control animals of the same age. Although not discussed in detail, a similar result was obtained by Ramanantsoa *et al.* (*51*) using a mouse model that also lacks a substantial number of dB2 retrotrapezoid nucleus neurons. Thus, the developmental impairment of dB2 retrotrapezoid nucleus can be largely compensated in postnatal life for approximately normal baseline breathing.

Albeit with different degrees of technical specificity, the acute optogenetic activation or inactivation of retrotrapezoid nucleus neurons, via viral transductions, can increase or reduce baseline respiratory frequencies and tidal volumes in adult mice (*52*–*55*). One should note, however, that these viral transductions and acute stimulations also targeted a substantial number of nearby catecholamine neurons, which can also change baseline respiration following the manipulation of their neural activity (*55*–*57*). Of note, catecholamine neurons are not dB2 derivatives as they originate from the dA3 progenitor domain (*29*, *58*) and are therefore not affected in our chemogenetic experiments. Here, we show that the chemogenetic activation of retrotrapezoid neurons, and other dB2 neurons, leads to augmented baseline tidal volumes and respiratory frequencies in neonate, juvenile, and adult mice, effects that might represent the induction of a hypercarbic-like response in CNO-*dB2-Activity* mice. In support of this, CNO-*dB2-Activity* neonates display changes in minute ventilation, tidal volumes, and respiratory cycle lengths (while breathing ambient air) that seem comparable to those observed in the same mice in hypercarbia (before CNO treatment). In adults, tidal volumes in CNO-*dB2-Activity* mice are also augmented, and their respiratory cycle lengths are reduced while breathing ambient air, but these changes do not reach the range of a typical hypercarbic response seen in adult mice when expose to the hypercarbic challenge.

We also show that inhibition of dB2 retrotrapezoid nucleus neurons, and other dB2 neuron subgroups, severely diminished baseline respiratory frequencies and tidal volumes in CNO-*dB2-Silence* neonates. The silencing of these cells only causes the reduction of tidal volumes but no impairment in baseline respiratory cycle lengths in CNO-*dB2-Silence* adult mice. In this context, a previous study reports no changes in baseline respiratory frequencies nor in ventilation after the selective chemogenetic inactivation of retrotrapezoid nucleus neurons in adult mice (*Nmb^CreERT2^* mice transduced with *AAV-DIO-hM4Di-mCherry* viral constructs) (*59*). Thus, the reduced baseline tidal volumes seen in CNO-*dB2-Silence* adult mice might not be caused by the chemogenetic inhibition of retrotrapezoid nucleus neurons but due to the inactivation of other dB2 neuron subgroups. One should note, however, that a recent study illustrates that the selective inhibition of retrotrapezoid nucleus neuron activity, via optogenetics, can change both baseline respiratory frequencies and tidal volumes in adult mice (*Nmb^Cre^* mice transduced with *AAV2-EF1a-DIO-eArch3.0-eYFP* viral constructs) (*50*). An important difference between the optogenetic and chemogenetic manipulation of retrotrapezoid nucleus neurons is the temporal mechanisms of action of these techniques. Optogenetic inhibition of neurons allows for a momentary and close to instantaneous (in the order of a few milliseconds) effect on their neural activity, thereby revealing the immediate function of the examined neural circuit. Chemogenetic inhibition of neurons allows for the study of long-lasting effects caused by the silencing of the interrogated circuit, and this can help to elucidate compensatory mechanisms that can counteract the inhibition of the investigated circuit, which, in the case of the retrotrapezoid nucleus, are largely mediated by the carotid body (*49*, *50*). Nonetheless, the inhibition of retrotrapezoid nucleus neuron activity either by chemogenetics or optogenetics produces similar blunted responses to hypercarbia [this work and (*50*)], which reveals the essential function of this medullary center in mediating the hypercarbic reflex.

The shallow respiratory tidal volumes seen in CNO-*dB2-Silence* mice, while breathing ambient air, might then be attributable to dB2 neurons located either in the medial vestibular nuclei (v1 and v2 subgroups) and/or dorsal to the facial motor nucleus (epiVII subgroup). Our systematic lineage tracing studies show that these neurons emerge from rhombomere 6, and their absence in *r5&6-Lbx1^FS^* mice causes comparable deficits in tidal volumes that phenocopy CNO-*dB2-Silence* animals. Early studies showed that electrical or chemical stimulation to activate or inhibit medial vestibular nuclei, or their afferents (cerebellar fastigial cells), can induce respiratory responses that augment or diminish ventilatory patterns via prominent effects on tidal volumes and that these changes are lost or attenuated by the bilateral destruction of the medial vestibular nuclei (*60*–*64*). Therefore, it is tempting to speculate that the diminished tidal volumes observed in CNO-*dB2-Silence* and *r5&6-Lbx1^FS^* mice might be a direct result of the inactivation and absence of medial vestibular dB2 neurons, respectively. Nonetheless, further work is necessary to exclude the newly identified epiVII subgroup from tidal volume control.

The apneic phenotype previously seen in homozygous *Lbx1^FS^* pups (*28*) and now here in *r5&6-Lbx1^FS^* and *MafB-Lbx1^FS^* newborns is not attributable to the lack of dB2 retrotrapezoid nucleus neurons, as no obvious change in the incidence of apneas is seen in *r3&5-Lbx1^FS^* newborns when compared to control littermates. On the contrary, this apneic phenotype correlates with the absence of dB2 neurons in either the medial vestibular nuclei or the epiVII region seen in *r5&6-Lbx1^FS^* newborns. Thus, we hypothesize here that either or both structures have an anti-apneic function in neonates. The sudden respiratory arrest seen in homozygous *Lbx1^FS^* pups (*28*) and reproduced here in *MafB-Lbx1^FS^* newborns seems to be attributable to the compound agenesis of most caudally (rhombomere 6) generated dB2 neuron subgroups. These include the periNA, epiNA, epiNTS, and infraSpV, in addition to medial vestibular (v1 and v2) dB2 subgroups and epiVII cells. We show that these neurons undergo marked fate changes during their development that prevent their correct specification. Future work is necessary to assess the potential function of these previously unknown populations in homeostatic respiration, as well as to define whether these cells individually or synergistically support neonatal survival.

Lbx1 and Phox2b impose specific neuron fates on largely non-overlapping neuron types in the medulla, spinal cord, and peripheral nervous system during development, with the unique cellular coincidence in development of dB2 neurons (*29*, *30*, *33*). Although ablation of *Lbx1* and *Phox2b* has long been known to cause agenesis of dB2 neurons and perinatal lethality in mice, due to profound respiratory arrest at birth, the complexity and additional phenotypes exhibited by *Lbx1* and *Phox2b* null mutant mice had previously made unfeasible the association of dB2 neurons with respiration (*18*, *26*, *28*, *31*, *32*, *34*, *65*). Similarly, the location, identities, and functions of these neurons had remained poorly understood. Here, we characterize the developmental origins of dB2 neurons and their relevance to respiration. We show that eight discrete medullary subgroups of neurons actively coexpress both Lbx1 and Phox2b at birth: intertrigeminal [rhombomere 2 derived; (*37*)], vestibular v1 and v2 (rostral rhombomere 6 derived; this work), vestibular v3 and v4 (rhombomere 4 derived; this work), retrotrapezoid nucleus neurons [rhombomere 5 derived; this work and (*28*, *34*, *36*, *51*)], epiVII (rostral rhombomere 6 derived; this work), and periNA (caudal rhombomere 6 derived; this work) neurons. However, lineage tracing of medullary neurons with a history of Lbx1 and Phox2b expression identifies four additional subgroups of cells that do not actively coexpress Lbx1 and Phox2b and might belong to the dB2 lineage: epiNA, epiNTS, and infraSpV (caudal rhombomere 6 derived) neurons and a few scattered neurons across the somatosensory trigeminal nuclei that we termed here as somaV (rhombomere 2 to 6 derived) neurons. The latter subgroup is unexpected as extensive research has previously shown that somatosensory trigeminal neurons originate from dB1 (Phox2b^−^) and dB3 (Phox2b^−^) late progenitors (about E13.5 in mice) (*29*, *31*–*32*). This indicates that a small proportion of dB2 (Phox2b^+^) early progenitor cells might be recruited to the pool of cells that generate somatosensory trigeminal neurons. In support of this, ventrally located Phox2b^+^ pMNv progenitor cells in the hindbrain transit from generating first visceral and branchial motor neurons to generating later serotonergic neurons of the raphe nuclei, a process that requires the extinction of Phox2b expression in pMNv progenitor cells [reviewed in (*30*, *33*)]. Therefore, although raphe neurons do not belong to the lineage of visceral and branchial motor neurons and have no history of active Phox2b expression, they can be marked in lineage tracing experiments when using recombinases driven by *Phox2b*, as seems to be the case for the scattered somaV neurons found in our intersectional lineage tracing experiments. However, this also poses the question as to whether epiNA, epiNTS, and infraSpV (not only the somaV) populations might also indeed belong to the dB2 lineage and participate in respiration.

Regardless of the philosophical question of how dB2 neurons can be defined, either by their active expression of Lbx1 and Phox2b or the expression history of these factors, we choose here the inclusive approach to define them according to their history of Lbx1 and Phox2b expression. Considering this approach, our work identifies specific subgroups of medullary dB2 neurons that coalesce into 12 nuclei, of which those that emerge from rhombomeres 5 and 6 are responsible for the respiratory phenotypes observed in patients with *LBX1^FS^* and in our murine models of congenital hypoventilation. Of note, rhombomere 5–derived somaV neurons are not involved in respiratory control, as the overlapping deficits in neonatal respiratory cycle lengths and the response to hypercarbia seen in *r3&5-Lbx1^FS^*, *r5&6-Lbx1^FS^*, and *MafB-Lbx1^FS^* mice can only be attributable to the known functions of dB2 retrotrapezoid nucleus neurons [this work and (*26*, *28*, *34*–*36*, *38*, *51*)]. Our study also uncovers that rhombomere 6 generates various neuron subgroups of the dB2 lineage, agenesis of which causes severe hypoventilation, pronounced neonatal respiratory instability, and neonatal mortality. The few scattered rhombomere 6–derived somaV cells might not be implicated in respiration, as this unique caudal population of dB2 neurons is unaffected in *Lbx1^FS^* (*dB2-Tomato-Lbx1^FS^*) mice. Nonetheless, if one chooses the pragmatic approach of defining dB2 neurons according to their active expression of Lbx1 and Phox2b, rather than according to their expression history, the respiratory phenotypes observed in our models of congenital hypoventilation can then be assigned to specific nuclei, namely, (i) to retrotrapezoid nucleus neurons for the impaired hypercarbic reflex and slow neonatal respiratory frequencies; (ii) to v1/v2 subgroups and epiVII neurons for the hypoventilation, neonatal respiratory instability, and apneas; and (iii) to periNA neurons for the neonatal respiratory arrest. Although intertrigeminal dB2 neurons, a rhombomere 2 derivative, are developmentally unaffected in homozygous *Lbx1^FS^* mice and are not targeted in *r5&6-Lbx1^FS^* nor in *MafB-Lbx1^FS^* newborns [this work and (*28*)], available evidence indicates that these cells might also play a role in respiration and respiratory-associated behaviors (*37*, *66*). Thus, multiple dB2 neuron subgroups are hard-wired to the central respiratory circuit. Collectively, our studies establish dB2 neuron dysfunction to be causative of congenital hypoventilation.

## MATERIALS AND METHODS

### Animals

All animal experimental procedures were approved by the Landesamt für Gesundheit und Soziales (G-0002/21) and the Charité Universitätsmedizin Berlin (TCH-0008/21). Mouse lines used in this study were as follows: *Egr2^Cre^* (*67*), *Lbx1^Cre^* (*68*), *Lbx1^lox^* (*28*), *Lbx1^FS^* (*28*), *MafB^Cre^* (*69*), *Phox2b^FlpO^* (*70*), *TgHoxb1^Cre^* (*71*, *72*), *TgHoxa3^Cre^* (*73*), *RC::FPDi* (*45*), *RC::FL-hM3Dq*, (*44*), *Rosa^LSL-nGFP^* (*74*), and *RCFL-tdT^+/−^* (*75*). All mouse lines were maintained for at least six generations in a C57BL/6N genetic background before the start of experimental procedures.

### Histology

Immunofluorescence, immunohistochemistry, and tissue processing were performed as previously described (*76*). In short, embryonic (E11.5, E12.5, E14.5, and E19) and postnatal brains (P0 and P56) were fixed in 4% paraformaldehyde, made in phosphate-buffered saline (PBS), for 4 hours at 4°C. After fixation, embryonic and postnatal brains were cryoprotected in 30% sucrose in PBS, embedded, and frozen in Tissue-Tek optimal cutting temperature (Sakura Finetek). Frozen brains were sectioned at 20 μm using a cryostat (Leica Biosystems). Sections were washed in PBS and blocked in PBS containing 5% normal goat serum (Sigma-Aldrich) (v/v) and 0.1% Triton X-100 (v/v) (Sigma-Aldrich) at room temperature for 2 hours. Sections were subsequently incubated in primary antibodies at room temperature overnight, washed in PBS, and further incubated in secondary antibodies prepared in blocking solution for 3 hours at room temperature. The following antibodies were used: rabbit anti-Atoh1 (1:1000; provided by T. Jessell, Columbia University), rabbit anti-cFos (1:3000; Cell Signaling Technology, 2250S), guinea pig anti-cFos (1:2000; Synaptic Systems, 226308), rabbit anti–glial fibrillary acidic protein (1:1000; Sigma-Aldrich, SAB5700611), goat anti-GFP (1:1000; Rockland, 600-101-215), rabbit anti-GFP (1:1000; MBL International, 598), rabbit anti-HA (1:50; Chromotek, 7C9), guinea pig anti-Lbx1 (1:10000) (*65*), guinea pig anti-Lmx1b (1:20,000) (*65*), goat anti-Phox2b (1:2000; R&D Systems, AF4940), goat anti-RFP (1:2000; Rockland, 200-101-379), rabbit anti-RFP (1:2000; Rockland, 600-401-379), and rabbit anti-Tac1r (1:2000; Sigma-Aldrich, AB5060). Cyanine dye 2 (Cy2)-, Cy3-, and Cy5-conjugated donkey anti-rabbit, anti-guinea pig, anti-goat secondary antibodies were obtained from Jackson ImmunoResearch and used at a concentration of 1:500. Horse anti-rabbit immunoglobulin G biotinylated antibody (1:250; Vector laboratories, BA-1100-1.5) was used for 3,3′-diaminobenzidine (DAB) staining in combination with the Vectastain Elite ABC-HRP (horseradish peroxidase; Vector laboratories, PK-6100) and the DAB Substrate Kit, Peroxidase (HRP) (Vector laboratories, SK-4100) as per the manufacturer’s instructions. Fluorescence signals were acquired with the following: (i) a Zeiss LSM 700 confocal microscope using the automatic tile scan modus (10% overlap between tiles) and assembled using ZEN2012, (ii) a Zeiss spinning disk confocal microscope using the automatic tile scan modus (10% overlap between tiles) and assembled using ZEN2012, (iii) a Nikon Widefield Ti2 microscope using the automatic tile scan modus (10% overlap between tiles) and assembled using Nikon elements viewer, and (iv) an Olympus BX51 epifluorescence microscope. The Nikon Widefield Ti2 microscope was also used to image DAB staining. All photomicrographs were acquired in a nonblind manner and processed for brightness and contrast corrections using Adobe Photoshop 2020.

### Cell quantifications

Cell quantifications were performed in a nonblind manner as previously described (*26*). Briefly, dB2 neuron and other cell quantifications were performed in nonconsecutive 20-μm-thick sections encompassing the complete anterior-posterior brainstem axis. On average, 80 to 85 sections were obtained per animal, and cells in every second section were bilaterally quantified and defined as subtotal of cells. The estimation of total number of cells was obtained by multiplying the subtotal of quantified cells by 2 (*26*).

### Brain clearing, lightsheet microscopy, and analysis

*dB2-Tomato* brains were cleared using the clear, unobstructed brain/body imaging cocktails and computational analysis protocol as previously described (*77*). Briefly, brains were dissected and fixed overnight at 4°C in 4% paraformaldehyde made in PBS. After washing overnight in PBS, lipids were removed using Reagent-1 (25% urea, 25% Quadrol, 15% Triton X-100, and 35% distilled H_2_O) at 37°C until brains were transparent (4 days). Brains were then washed overnight at 4°C in PBS to remove Reagent-1 and then placed into Reagent-2 (25% urea, 50% sucrose, 10% triethanolamine, 15% distilled H_2_O) at 37°C for refractive index matching (3 days). All reagents were acquired from Sigma-Aldrich. Once the brains were cleared, they were imaged using a Zeiss Lightsheet Z.1 microscope. 3D reconstructions, photos, and movies were created with arivis Vision4D. Movie editions were done using the commercial software Procreate and LumaFusion (LumaTouch LLC).

### Unrestrained whole-body plethysmography for juvenile and adult mice

Breathing recordings for juvenile (P21) and adult (P56) mice in ambient air, hypercarbia, or hypoxia were performed using previously reported protocols with minor modifications (*37*, *45*, *78*, *79*). Mice were placed in Data Science International (DSI) whole-body plethysmograph chambers (601-1425-001) and habituated for at least 1 hour on the experimental day. Mice were then individually recorded (one per chamber). Each breathing recording included an initial 30-min period of acclimatization (habituation) followed by a 20-min period of respiratory recordings in ambient air to determine baseline respiration. Respiratory recordings were taken in thermostable conditions (32°C) as previously recommended (*79*). Breathing recordings were acquired with a FinePointe Whole-Body Plethysmograph Unit with gas switch capability (DSI, 271-0500-290). The unit provided the plethysmograph chambers with a constant airflow (1 liter/min). Breathing waveforms were acquired with the New FinePointe Software (DSI, 271-0500-CFG). Body temperature and body weight were recorded for tidal volume estimation at the beginning of the respiratory recordings as previously described (*37*, *45*, *79*). Tidal volume estimations and respiratory cycle lengths were computed in the New FinePointe Software. Calculations for minute ventilation were obtained from the values of tidal volumes and respiratory cycle lengths using the New FinePointe Software. Protocols to induce hypercarbic and hypoxic responses in mice are published elsewhere (*37*, *45*, *79*) and schematically described in Figs. 8A and in fig. 26. Briefly, following the 20-min period to determine baseline respiration, mice were exposed to either a gas mixture of 21% O_2_, 8% CO_2_, balanced N_2_ (for hypercarbia) or 10% O_2_, balanced N_2_ (for hypoxia) for 10 min, followed by an additional 20-min period of postgas exposure. For determination of hypercarbic responses, the last 5 min of respiration in hypercarbia was compared to the last 5 min of respiratory recordings in ambient air (before gas exposure). For determination of hypoxic responses, the period encompassing 61 to 180 s (2 min) from the start of the gas exposure was compared to the last 2 min of respiratory recordings in ambient air (before gas exposure).

### Unrestrained whole-body plethysmography for neonatal mice

Breathing recordings for neonatal (P7) mice in ambient air, hypercarbia, or hypoxia were performed using the above-described protocols with the following modifications: Individual mice were placed in whole-body plethysmograph chambers suitable for pups (DSI, 601-1426-001). The plethysmographic chambers included a thermo-controlled warm bed set at 37°C. The FinePointe Whole-Body Plethysmograph Unit was adjusted to provide a constant airflow (0.5 liters/min).

### Head-out plethysmography for newborn mice

Breeding females were monitored daily for vaginal plugs. The day of vaginal plug identification was defined as E0.5. Pregnant dams were visually monitored for natural delivery from day E19 in intervals of 20 min. The onset of labor was recorded. On average, pregnant dams completed labor within 20 min. Newborns were recorded within 30 min from the onset of labor. The FinePointe Whole-Body Plethysmograph Unit was adjusted to provide a constant airflow (0.5 liters/min) and coupled to the DSI Digital Preamplifier (601-2401-001). The whole-body plethysmograph chambers suitable for pups were couple to the DSI head-out conversion kit (601-1533-001) and sealed with DSI latex collars (601-1533-002). The protocol used to induce hypercarbia in newborn mice is illustrated in Fig. 3E. Every recording included a 10-min acclimatization period followed by a 10-min period to determine baseline respiration, and mice were then exposed to 21% O_2_, 8% CO_2_, balanced N_2_ (for hypercarbia) for 5 min, followed by an additional 10-min period of postgas exposure. For determination of hypercarbic responses, the last 3 min of respiration in hypercarbia was compared to the last 5 min of respiratory recordings in ambient air (before gas exposure).

### Exclusion criteria

Plethysmograph chambers were covered with translucent red plastic covers. The experimenter visually monitored the recorded mice for potential movements during the analyzed periods. Movements were manually recorded, identified from the waveforms, and excluded from further analysis. On average, movements represented <10% of the recording time sessions. Mice actively vocalize in the neonatal period (P0 to P9). In previous work, we defined vocal breathing by concurrent plethysmography and auditory recordings using an UltraSoundGate condenser microphone capsule CM16 (sensitive to frequencies from 20 Hz to 180 kHz) and Avisoft Recorder software (sampling rate, 250 kHz; format, 16 bit) from Avisoft Bioacoustics (*58*). Vocal breathing was excluded from the respiratory analysis. It represented <5% of the recording time sessions in newborns and neonates.

### Intraperitoneal CNO injections

CNO (HelloBio, HB6149) was dissolved in sterile saline at a concentration of 10 mg/ml. Mice received a single intraperitoneal injection of CNO at a concentration of 10 mg/kg and subsequently placed in the plethysmographic chambers for 10 min before the start of acclimatization periods and downstream respiratory recordings.

### Whole-cell patch-clamp recordings

#### Slice preparation

Acute transverse 300-μm thick medullary slices were prepared from neonates (P5 to P7) and mature (P21 to P56) *dB2-Activity* (*Lbx1^Cre/+^;Phox2b^FlpO/+^;RC::FL-hM3Dq^+/−^*) and control (*Lbx1^+/+^;Phox2b^FlpO/+^;RC::FL-hM3Dq^+/−^*) mice in ice-cold (~4°C) sucrose-based artificial cerebrospinal fluid [sACSF; (pH 7.4) 85 mM NaCl, 26 mM NaHCO_3_, 50 mM sucrose, 10 mM glucose, 2.5 mM KCl, 1 mM NaH_2_PO_4_, 7 mM MgCl_2_, and 0.5 mM CaCl_2_; osmolarity, 290 to 310 milliosmole (mOsm)]. Slices were subsequently incubated in sACSF for 30 min. Slices were then transferred to modified Hepes-ACSF (92 mM NaCl, 30 mM NaHCO_3_, 25 mM glucose, 2.5 mM KCl, 1.2 mM NaH_2_PO_4_, 1.3 mM MgSO_4_, 2.5 mM CaCl_2_, 20 mM Hepes, 5 mM sodium ascorbate, 2 mM thiourea, and 3 mM sodium pyruvate; osmolarity, 290 to 310 mOsm) and stored at room temperature until subsequent usage. Individual slices were transferred to the recording chamber and perfused at a rate of ~5 ml/min with ACSF (119 mM NaCl, 26 mM NaHCO3, 10 mM glucose, 2.5 mM KCl, 1 mM NaH_2_PO_4_, 1.3 mM MgCl_2_, 2.5 mM CaCl_2_; osmolarity, 290 to 310 mOsm; pH 7.4). 6-Cyano-7-nitroquinoxaline-2,3-dione (20 μM) and D-AP5 (25 μM) were added to prevent excitatory network effects. All solutions were continuously carbonated (95% O_2_/5% CO_2_).

#### Recordings

Visually guided somatic whole-cell recordings were made from *dB2-Activity* (mCherry^+^) neurons and control (GFP^+^) neurons using borosilicate glass pipettes (3 to 6 megohm) at 34°C. Recordings were performed using a Multiclamp 700B amplifier (Molecular Devices, San Jose, CA, USA) or an EPC10 (HEKA, Reutlingen, Germany). Signals were filtered at 3 kHz, sampled at 10 kHz, and digitized using Digidata1550B and pClamp 11 (Molecular Devices) or patchmaster2.9.1 (HEKA). Neurons were characterized in current-clamp configuration by a series of increasing 1-s current steps at 0.2 Hz. The effect of CNO on membrane voltage was monitored with a continuous current clamp recording over 10 min.

#### Analysis

Recordings were analyzed using Axograph 1.8.0, fitmaster, and GraphPad Prism 10. Series resistance was measured at the beginning and end of each experiment, and recordings were discarded when the series resistance was above 30 megohm. Intersection of the linear regression between the relation of the firing rate increment versus the injected current (F-I relation; estimated in the early linear range) and abscissa approximated the rheobase.

### Statistics

Statistical analyses were performed using Prism 10 (GraphPad). Data are plotted in scatter dot plots, violin plots, or column dot plots with means and SDs. Normal distributions of the data were tested using D’Agostino and Pearson, Anderson-Darling, Shapiro-Wilk, and Kolmogorov-Smirnov tests. The statistical significance between group means was tested by one-way analysis of variance (ANOVA), followed by Tukey’s post hoc test (for multiple comparisons tests), or two-tailed *t* test (for pair comparison tests). Two-way nested ANOVA with Šidák post hoc test was used to determine changes in resting membrane potential or the action potential firing rate in whole-cell patch-clamp recordings to determine genotype-dependent effects. Poincaré plots were acquired using Prism 8. Poincaré plot analysis, including SD1 and SD2 quantification, was performed with Python (v3.10.5) using the Pandas, Numpy, and Matplotlib library via JupyterLab (v3.2.1). No statistical method was used to predetermine the sample size. No randomization or blinding was used for in vivo studies.

### Inclusion and diversity

We actively worked to maintain a 50 to 50% proportion of male and female mice throughout the in vivo and in vitro analysis presented in this study. One or more authors of this work self-identifies as an underrepresented ethnic minority in science. One or more authors of this work self-identifies as a nonbinary person.
