# Supplementary material for: Genetic identification of medullary neurons underlying congenital hypoventilation
Source: Sci Adv. 2024 Jun 19;10(25):eadj0720. doi: 10.1126/sciadv.adj0720 (PMC11186509; doi:10.1126/sciadv.adj0720)
Supplement: Supplementary file 1 — Figs. S1 to S26 Legend for movie S1 Legends for data S1 to S8 [file sciadv.adj0720_sm.pdf]

Science Advances
AAAS

Supplementary Materials for

Genetic identification of medullary neurons underlying congenital hypoventilation

Ke Cui *et al.*

Corresponding author: Yiling Xia, yiling.xia@charite.de;
Luis R. Hernandez-Miranda, luis.hernandez-miranda@charite.de

Sci. Adv. **10**, eadj0720 (2024)
DOI: 10.1126/sciadv.adj0720

The PDF file includes:

Figs. S1 to S26
Legend for movie S1
Legends for data S1 to S8

Other Supplementary Material for this manuscript includes the following:

Movie S1
Data S1 to S8

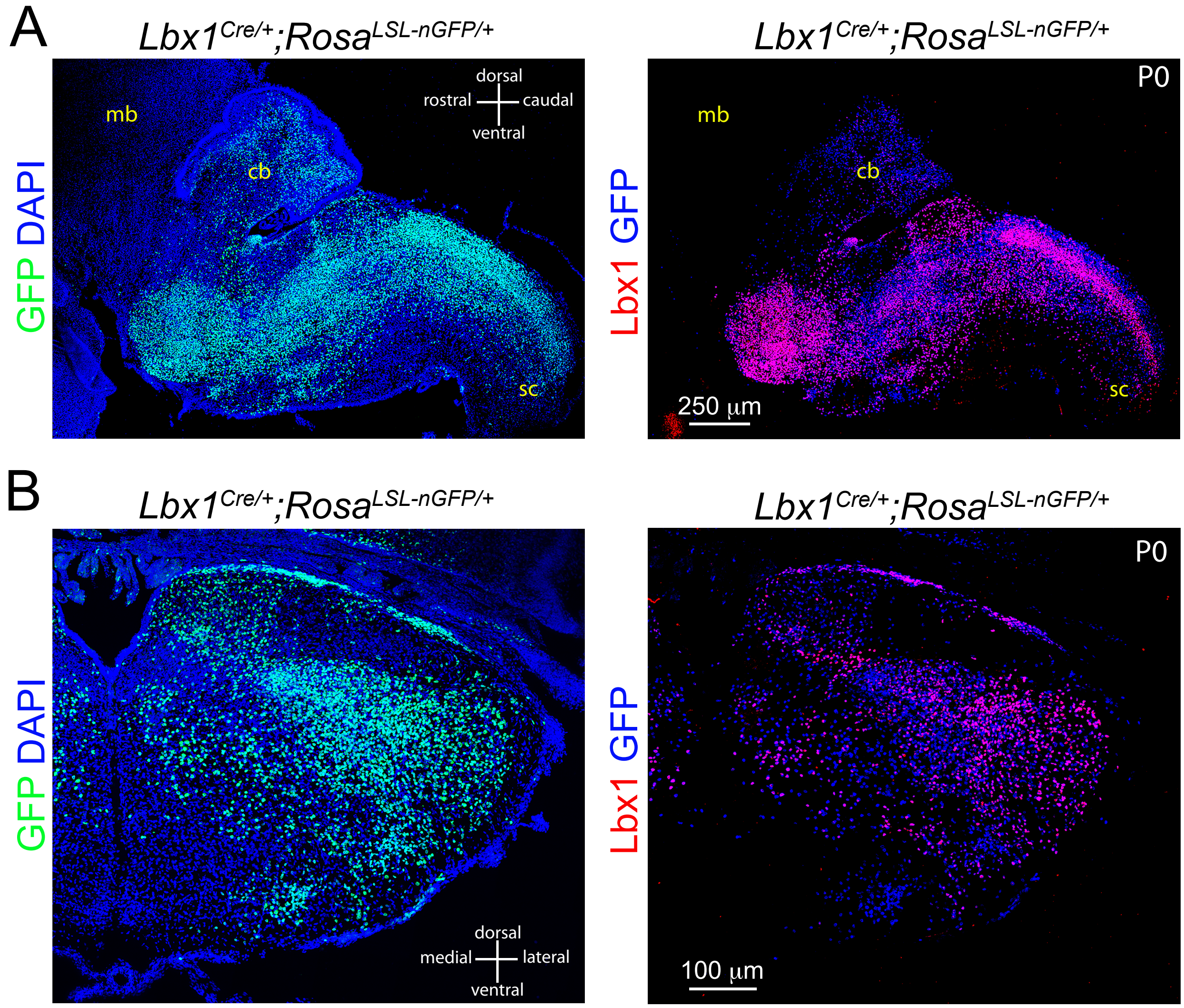

Fig. S1. Lineage-tracing of neurons with a history of *Lbx1* expression. (**A**) Left, a sagittal brainstem section taken from a *Lbx1*$^{Cre/+}$;*Rosa*$^{LSL\text{-}nGFP/+}$ mouse at birth (P0). The section was stained with green fluorescent protein (GFP, green) antibodies and DAPI (blue). Right, same section as on the left, but displaying GFP signals in blue to allow for a better visualization of Lbx1 (red) immunoreactive neurons (magenta). Please note that this photomicrograph is also displayed in Fig. 1C. (**B**) Left, a transverse medullary section taken from a *Lbx1*$^{Cre/+}$;*Rosa*$^{LSL\text{-}nGFP/+}$ mouse at birth. The section was stained with GFP (green) antibodies and DAPI (blue). Right, same section as on the left, but displaying GFP signals in blue to allow for a better visualization of Lbx1 (red) immunoreactive neurons (magenta). Note that by birth, most cells of the Lbx1 lineage (GFP+) have already downregulated the active expression of this factor.

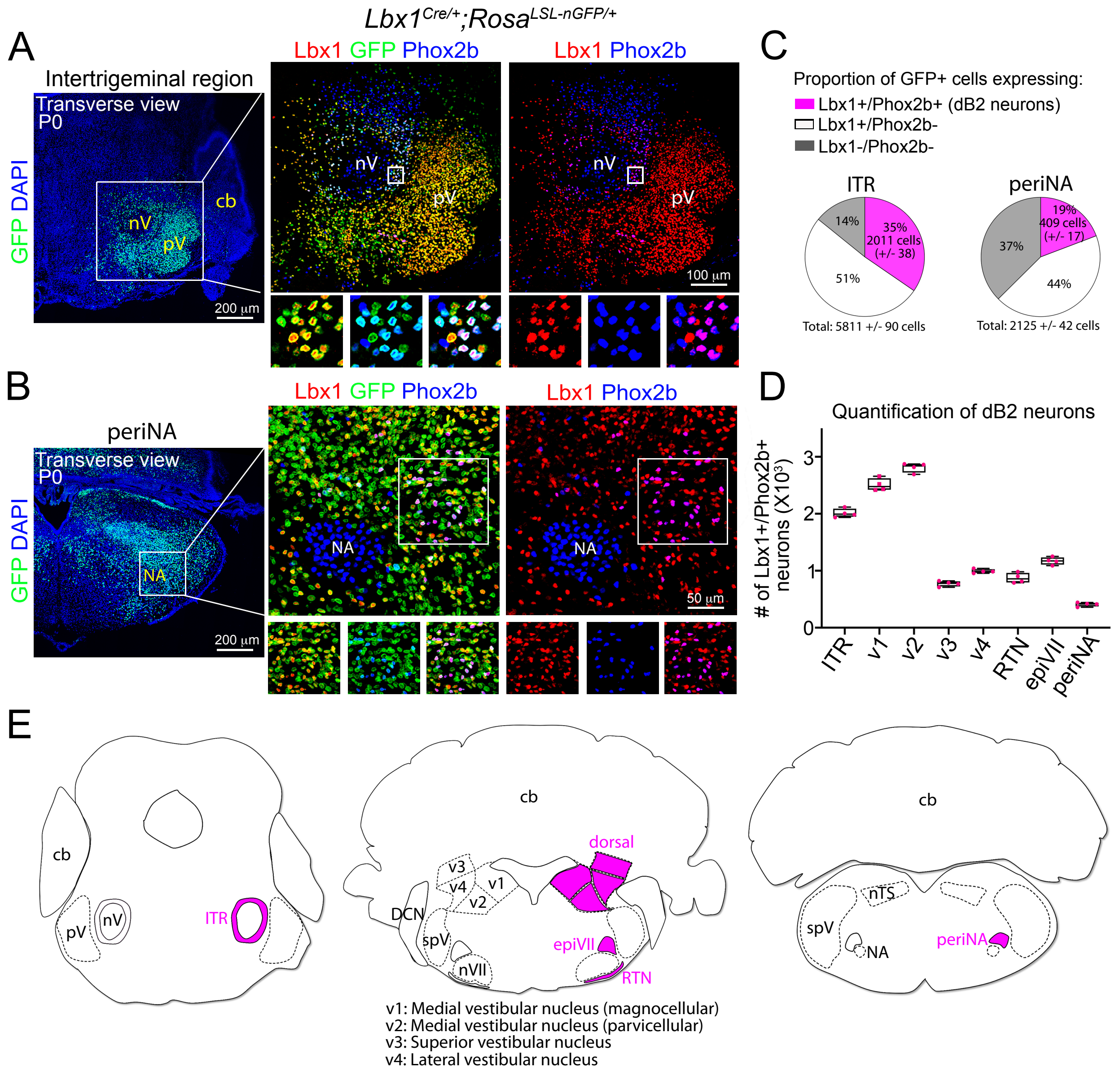

Fig. S2. Distribution of Lbx1+/Phox2b+ brainstem neurons. (**A**) Left, a transverse brainstem section at the level of the trigeminal motor (nV) nucleus in the pons stained with GFP (green) antibodies and DAPI (blue) at birth (P0). The section was taken from a *Lbx1*$^{Cre/+}$*;Rosa*$^{LSL\text{-}nGFP/+}$ mouse at birth (P0). The boxed area is shown on the right with Lbx1 (red), GFP and Phox2b (blue, false color) merged signals (in the middle), or Lbx1 and Phox2b signals (on the right) for a better visualization of the Lbx1 and Phox2b double positive (magenta) cells. The small boxed areas are shown magnified in the lower panels. (**B**) Left, a transverse medullary section at the level of nucleus ambiguus (NA) stained as above. The section was taken from a *Lbx1*$^{Cre/+}$*;Rosa*$^{LSL\text{-}nGFP/+}$ mouse at birth (P0). The boxed area is shown on the right with Lbx1, GFP and Phox2b merged signals (in the middle), or Lbx1 and Phox2b signals (on the right) for a better visualization of the Lbx1 and Phox2b double positive (magenta) cells. The small boxed areas are shown magnified in the lower panels. (**C**) Pie charts illustrating the proportion of cells in the intertrigeminal (ITR) and the peri nucleus ambiguus (periNA) subgroups of neurons with a history of Lbx1 expression (GFP+) and active expression of Lbx1 and Phox2b (dB2 neurons). (**D**) Quantification of Lbx1+/Phox2b+ (dB2) neurons in the intertrigeminal (ITR), vestibular (v1 to v4), retrotrapezoid (RTN), epifacial (epiVII), and peri nucleus ambiguus (periNA) subgroups. Every dot represents the mean of individual (n=4) animals. (**E**) Schematic transverse views of the mouse brainstem illustrating the location of Lbx1+/Phox2b+ (dB2) subgroups (magenta) at birth: i) intertrigeminal (ITR), vestibular (Ves; v1 to v4), epifacial (epiVII), retrotrapezoid nucleus (RTN), and peri nucleus ambiguus (periNA) neurons. The cerebellum (cb), principal trigeminal nucleus (pV), trigeminal motor nucleus (nV), dorsal cochlear nucleus (DCN), nucleus tractus solitarius (nTS), spinal trigeminal nucleus (SpV), as well as the facial (nVII) and ambiguus (NA) motor nuclei are illustrated for anatomical orientation. Tabulated data can be found in Data S8.

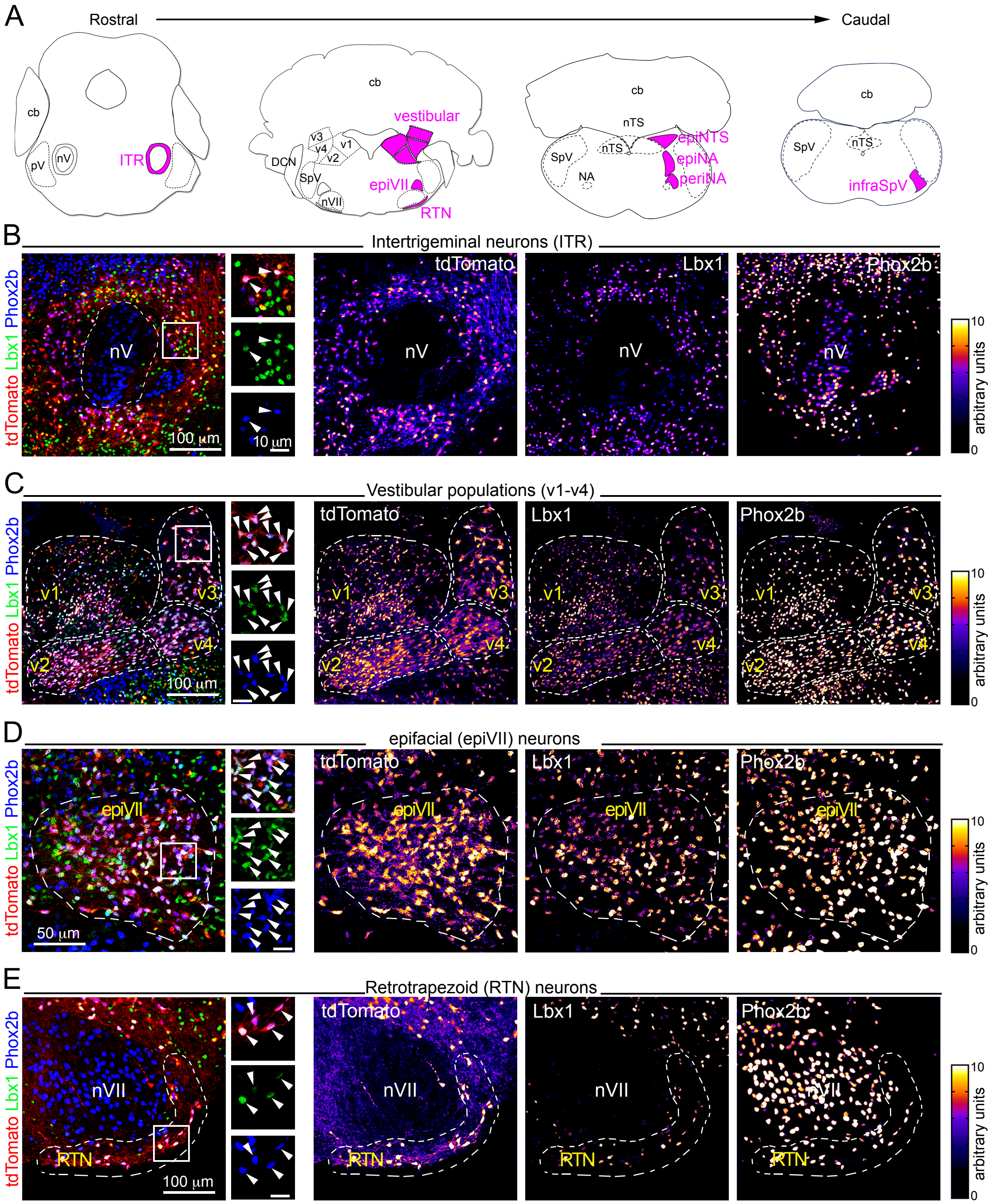

Fig. S3. Analysis of rostral dB2 neurons. (**A**) Schematic transverse views of the mouse brainstem illustrating the location of Lbx1+/Phox2b+ (dB2) neuron subgroups (magenta) identified with tdTomato (in *dB2-Tomato* mice) at birth: i) intertrigeminal (ITR), vestibular (Ves; v1 to v4), epifacial (epiVII), retrotrapezoid nucleus (RTN), peri nucleus ambiguus (periNA), epi nucleus ambiuus (epiNA), epi nucleus tractus solitarius (epiNTS) and infra spinal somatosensory trigeminus nucleus (infraSpV) neurons. Note that the scattered tdTomato+ neurons observed in the somatosensory trigeminal nuclei (called here somaV) are not illustrated in these schematic illustrations (see text). The cerebellum (cb), principal somatosensory trigeminal nucleus (pV), trigeminal motor nucleus (nV), dorsal cochlear nucleus (DCN), nucleus tractus solitarius (nTS), spinal trigeminal nucleus (SpV), as well as the facial (nVII) and ambiguus (NA) motor nuclei are illustrated for anatomical orientation. (**B-E**) Left, histological analysis of ITR, vestibular, epiVII, and retrotrapezoid nucleus (dB2) neurons in *dB2-Tomato* newborn mice stained against the red fluorescent protein (to detect tdTomato, red), Lbx1 (green) and Phox2b (blue, false color). The boxed areas are displayed on the right illustrating merged or single fluorescent signals. Arrows indicate Lbx1+/Phox2b+/tdTomato+ triple positive cells. Right, protein heatmaps for tdTomato, Lbx1 and Phox2b from the immunofluorescent photographs displayed on the left panels.

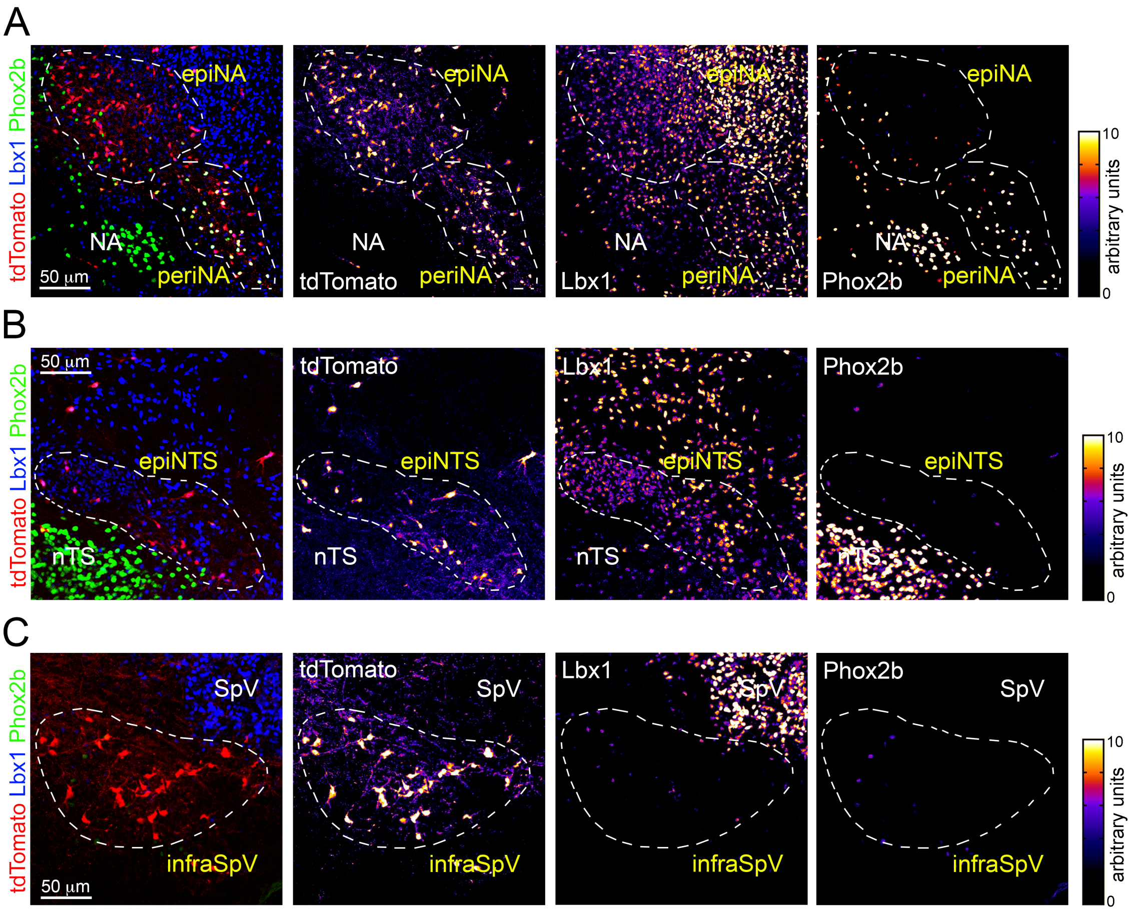

D

dB2 neuron population	Active co-expression of Lbx1 and Phox2b
ITR	Yes
Ves (v1-v4)	Yes
epiVII	Yes
RTN	Yes
periNA	Yes
epiNA	No
epiNTS	No
infraSpV	No
somaV	No

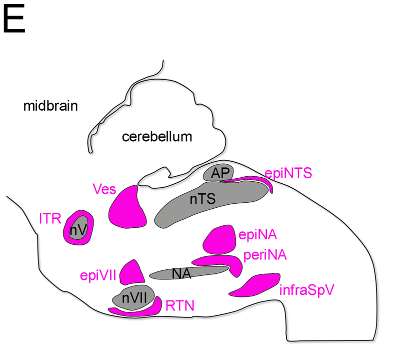

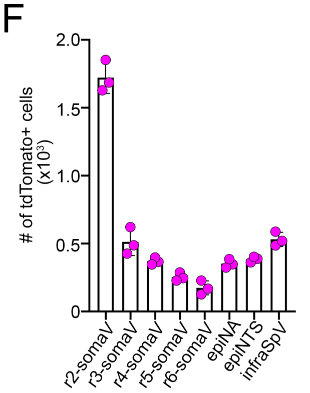

Fig. S4. Analysis of caudal dB2 neurons. (**A-C**) Left, histological analysis of epi nucleus ambiguus (epiNA), epi nucleus tractus solitarius (epiNTS) and infra spinal somatosensory trigeminal nucleus (infraSpV) neurons in *dB2-Tomato* newborn mice stained against the red fluorescent protein (to detect tdTomato, red), Lbx1 (blue, false color) and Phox2b (green). Note that these images are taken from Fig. 2, F and H, to illustrate the protein heatmaps for tdTomato, Lbx1 and Phox2b displayed on the right. (**D**) Table illustrating the expression pattern of Lbx1 and Phox2b on the identified tdTomato+ dB2 neuron subgroups in this study at birth: intertrigeminal (ITR), vestibular (v1-v4), epifacial (epiVII), retrotrapezoid (RTN), epi nucleus ambiguus (epiNA) peri nucleus ambiguus (periNA), epi nucleus tractus solitarius (epiNTS), and infra spinal trigeminal nucleus (infraSpV). This table also includes the scattered tdTomato+ neurons observed in the somatosensory trigeminal nuclei (called here somaV, see text). (**E**) Schematic view of a sagittal brainstem section illustrating the location of dB2 neurons (in magenta) identified in this study. Note that somaV neurons are not illustrated in this schematic view as their lateral location in the brainstem cannot be correctly depicted in a 2D schematic display. The area postrema (AP), nucleus tractus solitarius (nTS) as well as the facial (nVII) and ambiguus (NA) motor nuclei are illustrated for anatomical orientation. (**F**) Quantification of caudal dB2 (tdTomato+) neurons: epiNA, epiNTS, infraSpV, as well as r2- to r6-derived somaV neurons in *dB2-Tomato* newborn (n=3) mice. Quantification of the other dB2 neuron subgroups can be found in fig. S2D. Tabulated data can be found in Data S8.

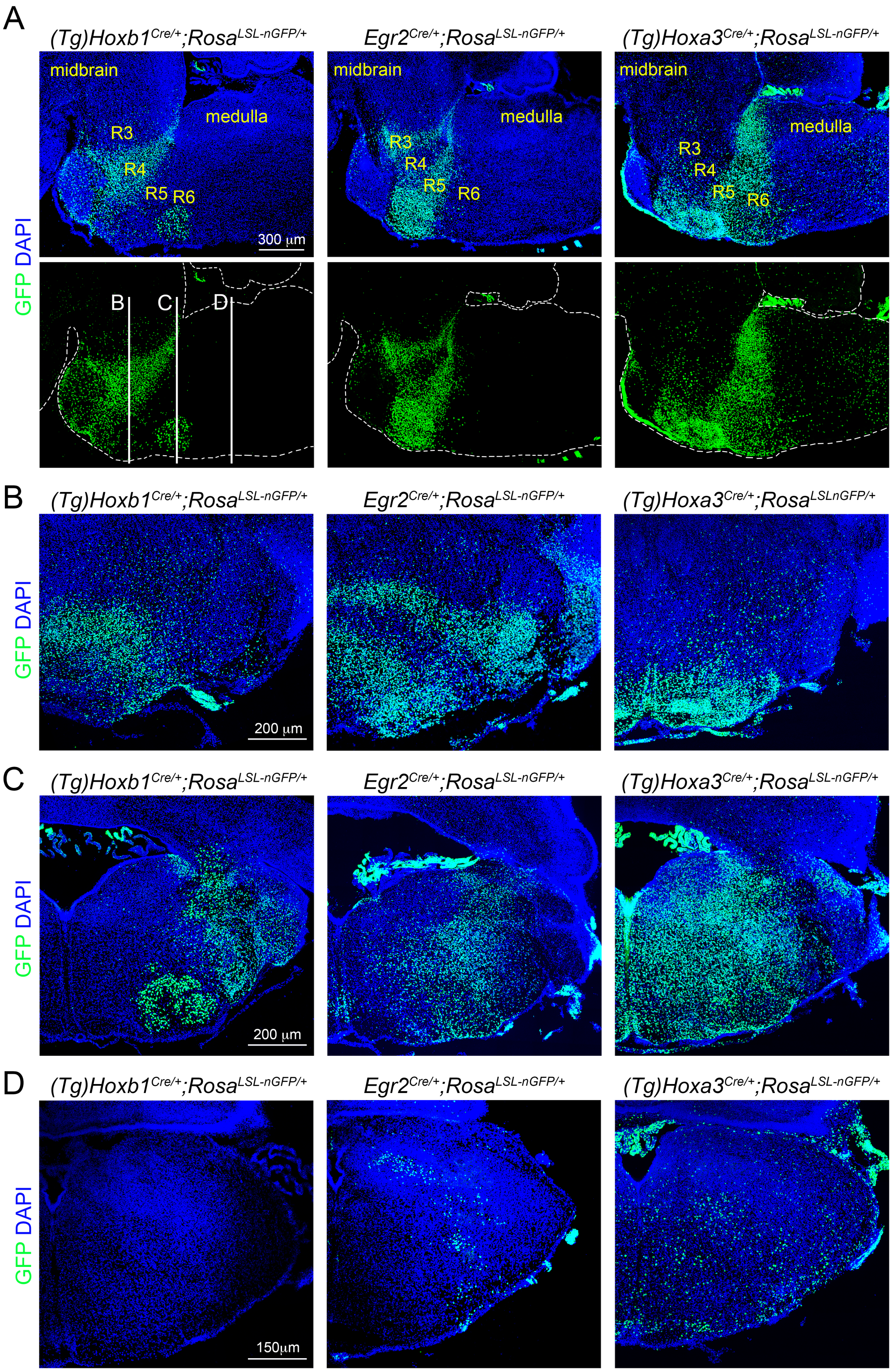

Fig. S5. Recombination patterns of *(Tg)Hoxb1*^{Cre}, *Egr2*^{Cre} and *(Tg)Hoxa3*^{Cre} driver lines. (**A**) Sagittal brainstem sections taken from *(Tg)Hoxb1*$^{Cre/+}$*;Rosa*$^{LSL\text{-}nGFP/+}$, *Egr2*$^{Cre/+}$*;Rosa*$^{LSL\text{-}nGFP/+}$ and *(Tg)Hoxa3*$^{Cre/+}$*;Rosa*$^{LSL\text{-}nGFP/+}$ newborn mice. The sections were stained with GFP (green) antibodies and DAPI (blue). Upper panels, cells derived from rhombomeres 3 to 6 are indicated. Lower panels, same sections as above displaying GFP signals only. The vertical lines in *(Tg)Hoxb1*$^{Cre/+}$*;Rosa*$^{LSL\text{-}nGFP/+}$ mice denote the transverse section planes illustrated in panels B-D. (**B-D**) Transverse brainstem sections taken from *(Tg)Hoxb1*$^{Cre/+}$*;Rosa*$^{LSL\text{-}nGFP/+}$, *Egr2*$^{Cre/+}$*;Rosa*$^{LSL\text{-}nGFP/+}$ and *(Tg)Hoxa3*$^{Cre/+}$*;Rosa*$^{LSL\text{-}nGFP/+}$ newborn mice and stained with GFP antibodies and DAPI.

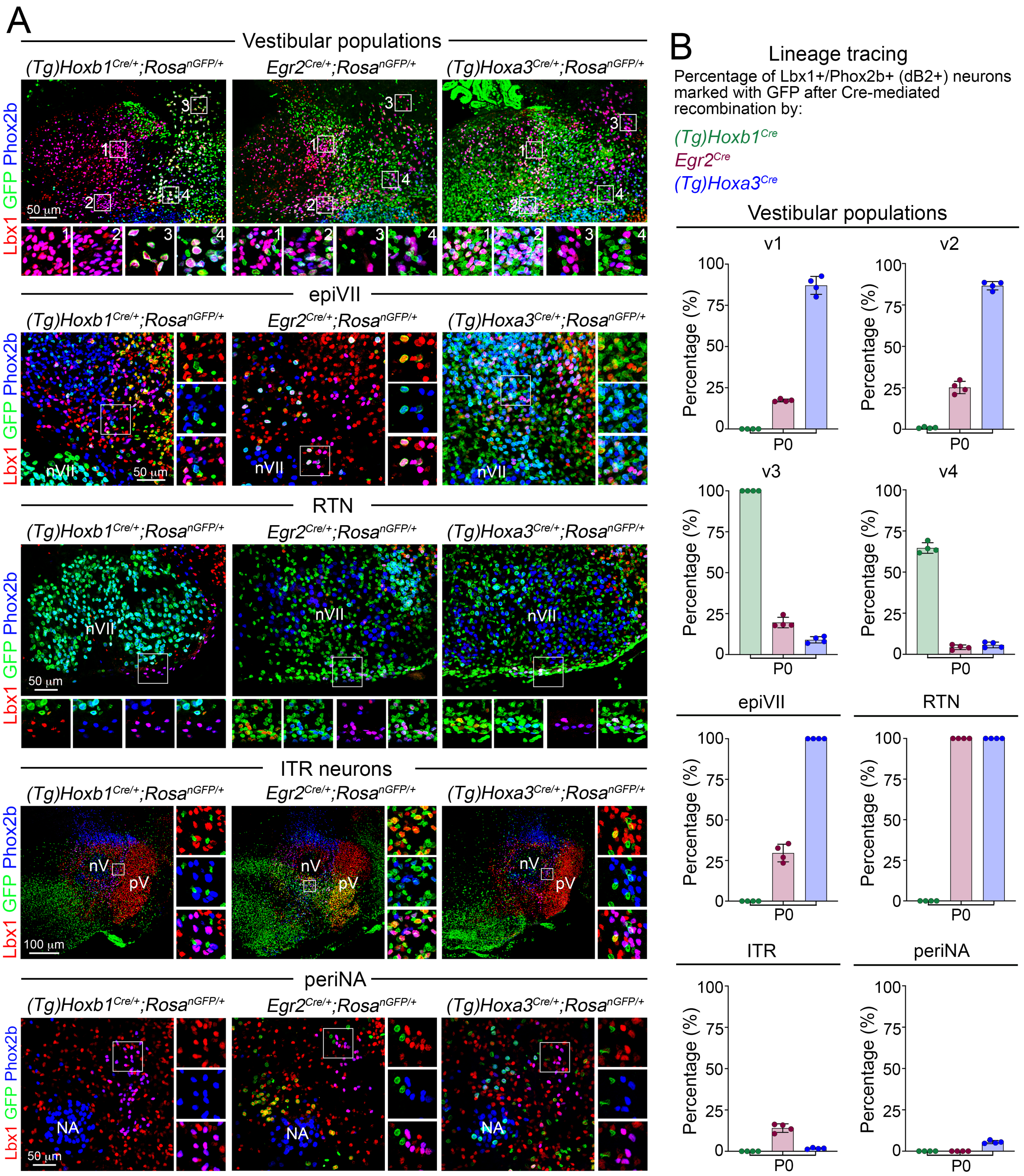

Fig. S6. Lineage-tracing of dB2 neurons using the *(Tg)Hoxb1^{Cre}*, *Egr2^{Cre}* and *(Tg)Hoxa3^{Cre}* Cre-driver lines. (**A**) Transverse brainstem sections taken from *(Tg)Hoxb1$^{Cre/+}$;Rosa$^{LSL\text{-}nGFP/+}$*, *Egr2$^{Cre/+}$;Rosa$^{LSL\text{-}nGFP/+}$* and *(Tg)Hoxa3$^{Cre/+}$;Rosa$^{LSL\text{-}nGFP/+}$* newborn mice stained with antibodies against GFP (green), Lbx1 (red) and Phox2b (blue, false color). Please note that due to space restrictions the *Rosa$^{LSL\text{-}nGFP/+}$* reporter is abbreviated only as *Rosa$^{nGFP/+}$* in this figure. The boxed areas are magnified at the bottom, or at the right, of the main panels. The principal trigeminal nucleus (pV), as well as the facial (nVII), trigeminal (nV), and ambiguus (NA) motor nuclei are indicated for anatomical orientation. (**B**) Quantification of the proportions of dB2 (Lbx1+/Phox2b+) neurons co-expressing GFP in each genotype analyzed, color code: *(Tg)Hoxb1^{Cre}* (green; n=4 mice), *Egr2^{Cre}* (maroon; n=4 mice) and *(Tg)Hoxa3^{Cre}* (blue; n=4 mice). Tabulated data can be found in Data S8.

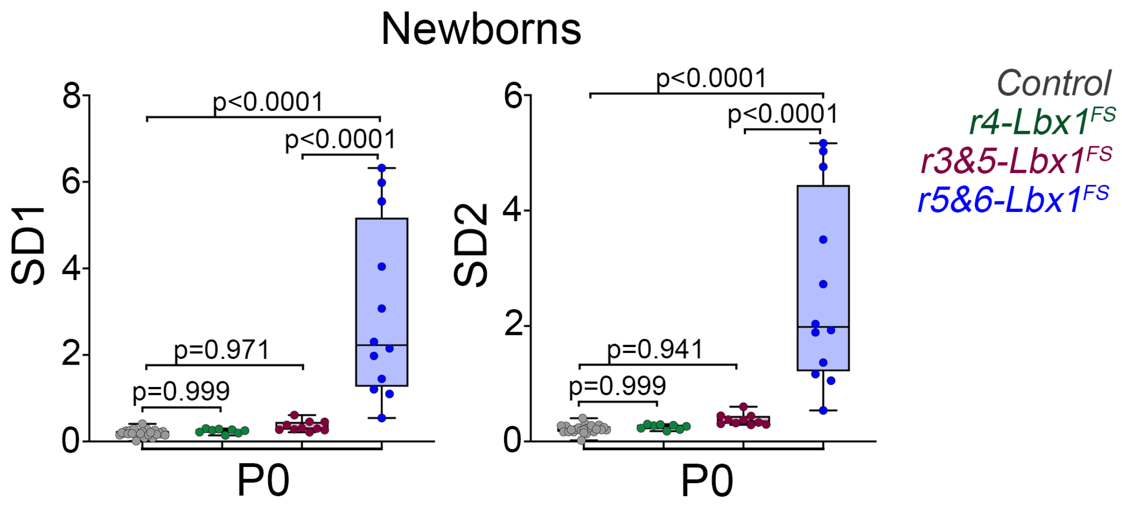

Fig. S7. Respiratory instability in *r4-Lbx1^{FS}*, *r3&5-Lbx1^{FS}*, and *r5&6-Lbx1^{FS}* newborn mice. Quantification of standard deviation (SD) 1 and SD2 in *Control* (n=24 mice*)*, *(Tg)Hoxb1$^{Cre/+}$;Lbx1$^{FS/lox}$* (*r4-Lbx1^{FS}*; n=8 mice), *Egr2$^{Cre/+}$;Lbx1$^{FS/lox}$* (*r3&5-Lbx1^{FS}*; n=10 mice), and *(Tg)Hoxa3$^{Cre/+}$;Lbx1$^{FS/lox}$* (*r5&6-Lbx1^{FS}*; n=12 mice) newborn (P0) mice. Each dot represents the mean of individual mice analyzed. Significance was determined using one-way ANOVA followed by post hoc Tukey's analysis. Tabulated data can be found in Data S8.

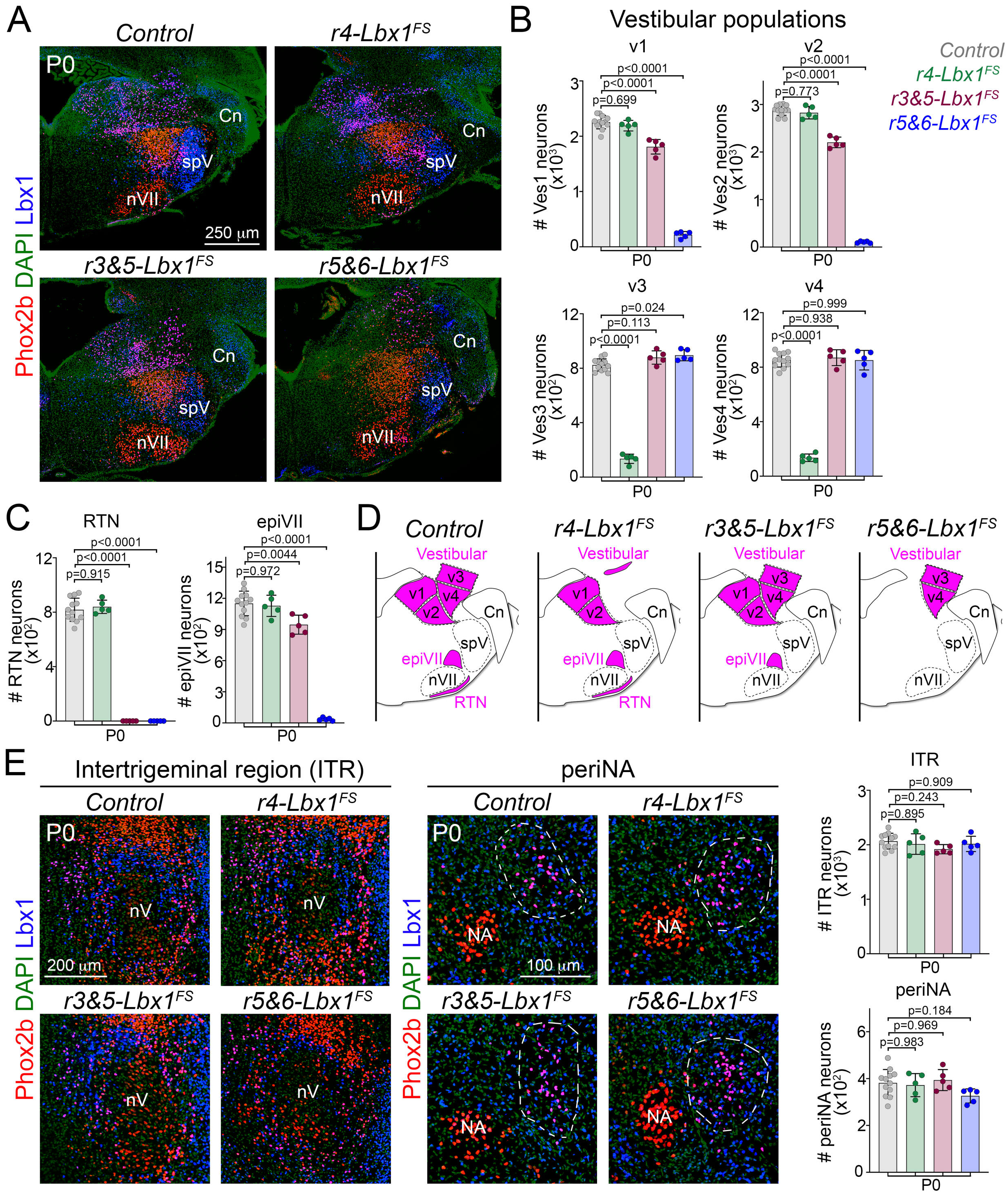

Fig. S8. Figure caption on next page.

Fig. S8. Agenesis of dB2 neurons in *r4-Lbx1^{FS}, r3&5-Lbx1^{FS}, and r5&6-Lbx1^{FS}* mice. (**A-C**) Histological analysis and quantification of vestibular (v1-v4 subgroups), retrotrapezoid nucleus (RTN) neurons and epifacial (epiVII) dB2 neurons in *Control*, *(Tg)Hoxb1$^{Cre/+}$;Lbx1$^{FS/lox}$* (*r4-Lbx1^{FS}*), *Egr2$^{Cre/+}$;Lbx1$^{FS/lox}$* (*r3&5-Lbx1^{FS}*), and *(Tg)Hoxa3$^{Cre/+}$;Lbx1$^{FS/lox}$* (*r5&6-Lbx1^{FS}*) newborn (P0) mice. Transverse brainstem sections were stained with antibodies against Lbx1 (blue, false color) and Phox2b (red), and counterstained with DAPI (green, false color). The cochlear nucleus (Cn), spinal trigeminal (SpV), and facial (nVII) motor nuclei are indicated for anatomical orientation. Please note that the magnifications shown in Fig. 3H were taken from the images presented here in panel A. (**D**) Schematic summary of the dB2 neuron distribution in the indicated genotypes. (**E**) Histological analysis and quantification of intertrigeminal region (ITR) and peri nucleus ambiguus (periNA) neurons in *Control*, *r4-Lbx1^{FS}*, *r3&5-Lbx1^{FS}*, and *r5&6-Lbx1^{FS}* newborn (P0) mice. The transverse brainstem sections were stained with antibodies against Lbx1 (blue, false color) and Phox2b (red), and counterstained with DAPI (green, false color). The trigeminal (nV) and ambiguus (NA) motor nuclei are indicated for anatomical orientation. Every dot in the quantifications represents the mean of individual animals. Significance was determined using one-way ANOVA followed by post hoc Tukey's analysis. Tabulated data can be found in Data S8.

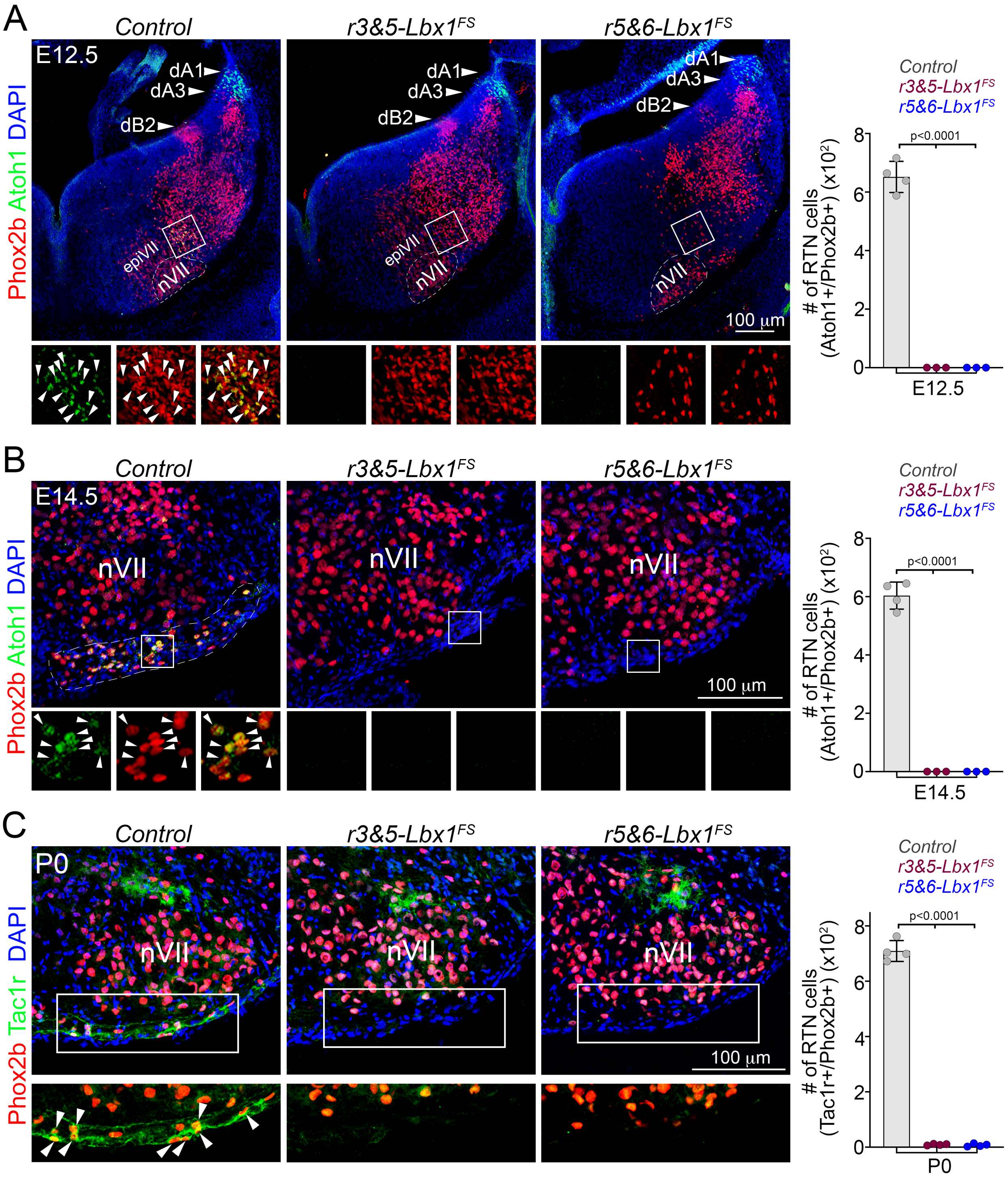

Fig. S9. Figure caption on next page.

Fig. S9. Agenesis of dB2 retrotrapezoid nucleus neurons during development of *r3&5-Lbx1^{FS}* and *r5&6-Lbx1^{FS}* mice. During early development in mice, about embryonic (E) day 11.5, dB2 progenitor cells differentiate into dB2 neurons that co-express Lbx1 and Phox2b; a subset of these neurons (from rhombomere 5) migrates to the ventral medulla and activates *en route* expression of *Atoh1* by E12.5, as they reach the facial motor (nVII) nucleus. The co-expression of Lbx1, Phox2b, and Atoh1, is the bona fide molecular signature of dB2 retrotrapezoid nucleus neuron identity (refs. *28*, *34*). Ablation of each of these genes leads to the anatomical absence (in *Phox2b* and *Lbx1* mutant mice) or aberrant location (in *Atoh1* mutants) of dB2 retrotrapezoid nucleus neurons, as well as to the loss of the hypercarbic reflex and neonatal death (reviewed in *30, 33*). By E14.5 in mice, retrotrapezoid nucleus neurons settle underneath nVII (refs. *28*, *34*). (**A, B**) Histological analysis and quantification of Atoh1+/Phox2b+ dB2 retrotrapezoid nucleus neurons in *Control*, *Egr2$^{Cre/+}$;Lbx1$^{FS/lox}$* (*r3&5-Lbx1^{FS}*), and *(Tg)Hoxa3$^{Cre/+}$;Lbx1$^{FS/lox}$* (*r5&6-Lbx1^{FS}*) embryos. The transverse sections were stained with antibodies against Atoh1 (green) and Phox2b (red). DAPI was used to counterstained (blue). Boxed areas are shown magnified below the main panels with merged and separate signals for Phox2b and Atoh1. (**A**) At E12.5, migrating dB2 retrotrapezoid nucleus neurons (arrowheads) can be distinguished by the co-expression of Atoh1 and Phox2b in *Control*, but not in *r3&5-Lbx1^{FS}* or *r5&6-Lbx1^{FS},* embryos. The progenitor domains dA1 (Atoh1+), dA3 (Phox2b-) and dB2 (Phox2b+), as well as the facial motor (nVII) nucleus and the epifacial (epiVII) dB2 subgroups are indicated. For a review of hindbrain progenitor domains see refs. *30* and *33*. (**B**) At E14.5, Atoh1+/Phox2b+ dB2 retrotrapezoid nucleus neuron neurons can be seen underneath the nVII in *Control* embryos, but not in *r3&5-Lbx1^{FS}* or *r5&6-Lbx1^{FS}* E14.5 embryos. (**C**) Histological analysis and quantification of Tac1r+/Phox2b+ dB2 retrotrapezoid nucleus neurons in *Control*, *r3&5-Lbx1^{FS} and r5&6-Lbx1^{FS}* newborn (P0) mice. Boxed areas are magnified at the bottom of the main photographs displaying signals for Tac1r and Phox2b only for easy visualization. Note the absence of dB2 retrotrapezoid nucleus neurons in *r3&5-Lbx1^{FS} and r5&6-Lbx1^{FS}* newborn mice. See also fig. S10 and fig. S14C. Every dot in the quantifications represents the mean of individual animals. Significance was determined using one-way ANOVA followed by post hoc Tukey's analysis. Tabulated data can be found in Data S8.

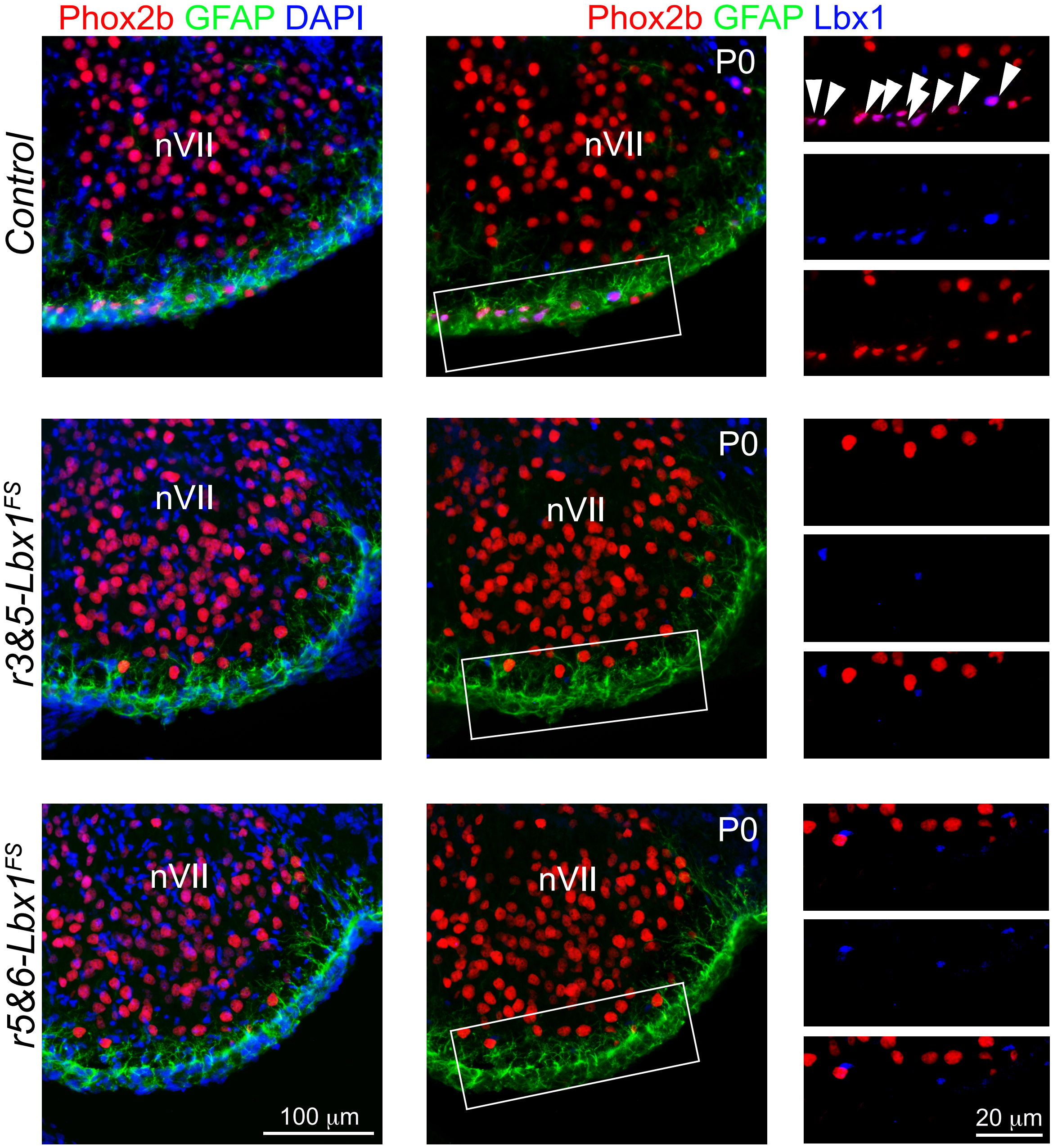

Fig. S10. Analysis of dB2 retrotrapezoid nucleus neurons in *r3&5-Lbx1^FS^* and *r5&6-Lbx1^FS^* mice at birth. Transverse sections taken from *Control*, *Egr2^Cre/+^;Lbx1^FS/lox^* (*r3&5-Lbx1^FS^*), and *(Tg)Hoxa3^Cre/+^;Lbx1^FS/lox^* (*r5&6-Lbx1^FS^*) newborn (P0) mice. The sections were stained with antibodies against glial fibrillary acidic protein (GFAP; green), Phox2b (red) and Lbx1 (blue, false color). DAPI (blue) was used to counterstain. The left panels display GFAP, Phox2b and DAPI fluorescence signals, whereas the middle panels display GFAP, Phox2b and Lbx1 fluorescence signals. The boxed areas in the middle panels are magnified at the right displaying only Lbx1 and Phox2b fluorescence signals to visualize better Lbx1+/Phox2b+ dB2 retrotrapezoid nucleus neurons (arrowheads) in control mice and their lack in *r3&5-Lbx1^FS^* and *r5&6-Lbx1^FS^* mice. Note that the astrocytic bed covering the ventral surface underneath the facial (nVII) motor nucleus remains unaltered in *r3&5-Lbx1^FS^* and *r5&6-Lbx1^FS^* newborn mice. The pia mater, a fine layer covering the brain nervous tissue, can still be observed underneath the astrocytic bed in the illustrated pictures. Quantification of Lbx1+/Phox2b+ dB2 retrotrapezoid nucleus neurons in *r3&5-Lbx1^FS^* and *r5&6-Lbx1^FS^* mice can be found in fig. S8C.

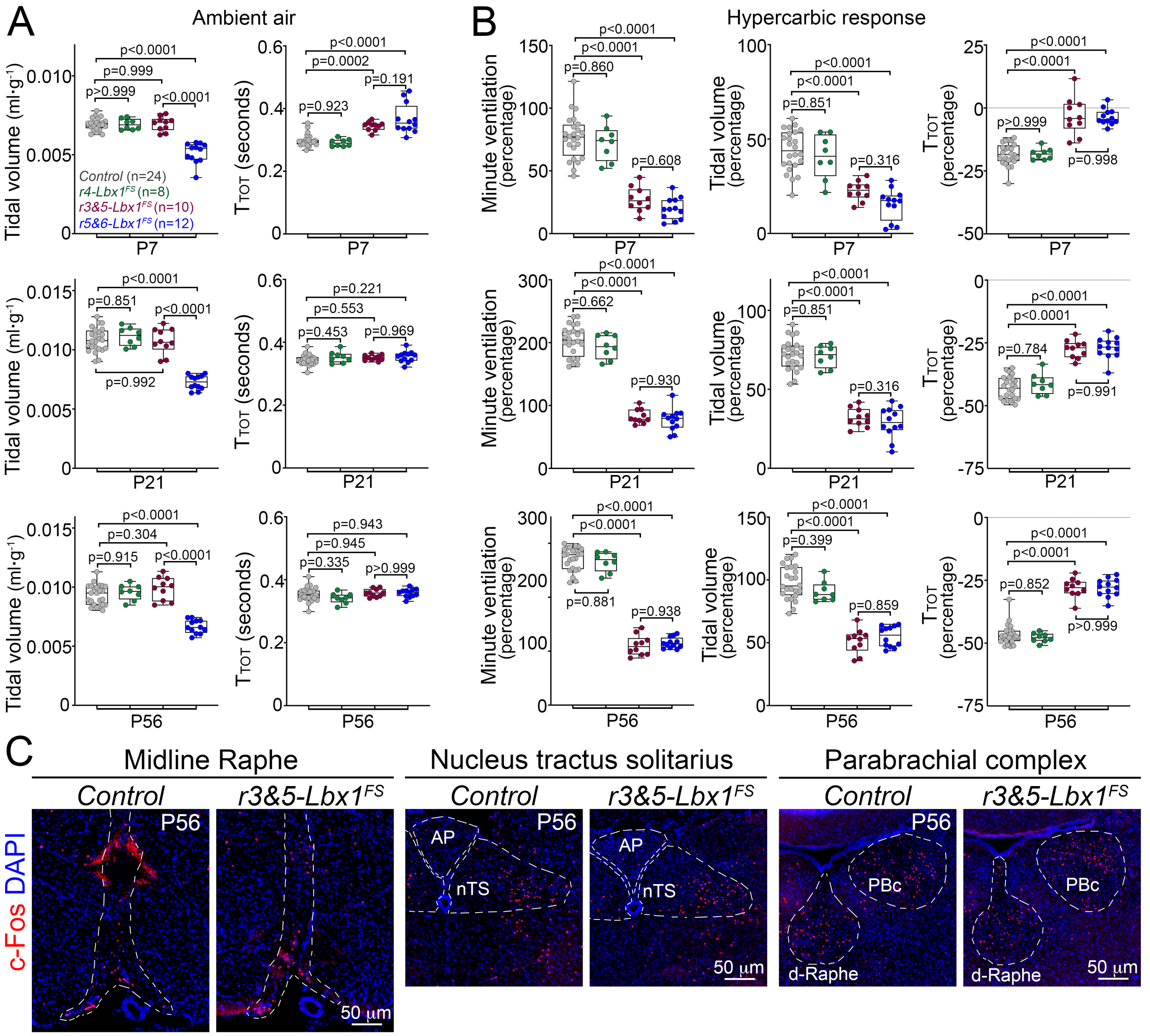

Fig. S11. Breathing of *$r4$-$Lbx1^{FS}$*, *$r3\&5$-$Lbx1^{FS}$*, *and $r5\&6$-$Lbx1^{FS}$* mice in the postnatal life. (**A**) Quantification of tidal volumes and respiratory cycle length (T_{TOT}) in *Control*, *(Tg)$Hoxb1^{Cre/+}$;$Lbx1^{FS/lox}$* (*$r4$-$Lbx1^{FS}$*), *$Egr2^{Cre/+}$;$Lbx1^{FS/lox}$* (*$r3\&5$-$Lbx1^{FS}$*), and *(Tg)$Hoxa3^{Cre/+}$;$Lbx1^{FS/lox}$* (*$r5\&6$-$Lbx1^{FS}$*) mice while breathing ambient air, at P7 (top), P21 (middle) and P56 (bottom). The number (n) of mice analyzed are displayed in brackets next to the genotypes. Tabulated data can be found in Data S3. (**B**) Respiratory response to hypercarbia expressed as percentage of change relative to the baseline (ambient air). Change of minute ventilation, tidal volumes, and respiratory cycle lengths (T_{TOT}) displayed by the indicated genotypes at P7 (Top), P21 (middle) and P56 (bottom). Tabulated data can be found in Data S3. Every dot in the quantifications represents the mean of individual animals. Significance was determined using one-way ANOVA followed by post hoc Tukey's analysis. (**C**) Histological characterization of c-Fos+ cells in the midline raphe (raphe obscurus, pallidus and magnus), the nucleus tractus solitarius (nTS) and parabrachial complex (PBc) in *Control* and *$r3\&5$-$Lbx1^{FS}$* adult mice after an hour-long exposure to hypercarbia. The area postrema (AP) is also indicated for anatomical orientation. Note that the dorsal raphe (d-Raphe) also contains c-Fos activated cells after hypercarbic exposure. Quantification of c-Fos+ cells per each of the illustrated regions in this figure can be found in Fig. 4, E-G.

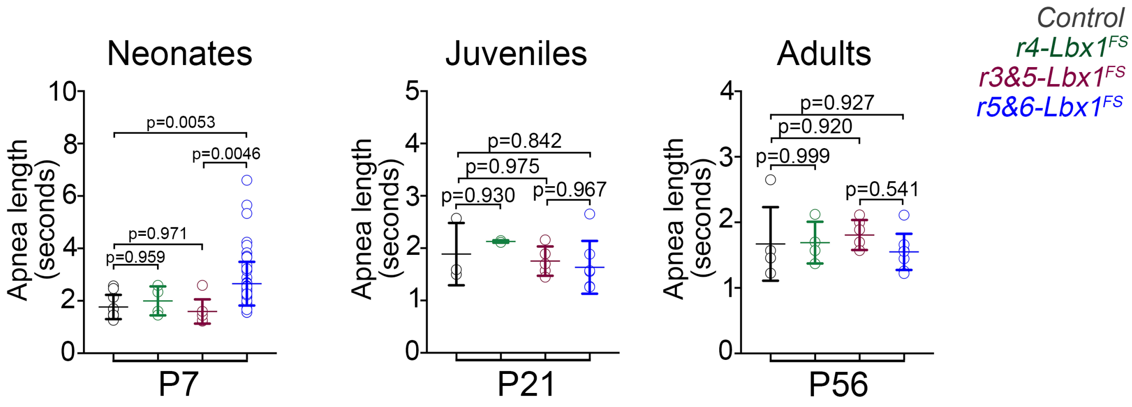

Fig. S12. Apnea incidence in *r4-Lbx1^{FS}*, *r3&5-Lbx1^{FS}*, and *r5&6-Lbx1^{FS}* mice at different ages. Quantification of apnea lengths in *Control* (n=24), *(Tg)Hoxb1$^{Cre/+}$;Lbx1$^{FS/lox}$* (*r4-Lbx1^{FS}*; n=8), *Egr2$^{Cre/+}$;Lbx1$^{FS/lox}$* (*r3&5-Lbx1^{FS}*; n=10), and *(Tg)Hoxa3$^{Cre/+}$;Lbx1$^{FS/lox}$* (*r5&6-Lbx1^{FS}*; n=12) mice while breathing ambient air at the indicated stages. Each circle represents individual apneas. Significance was determined using one-way ANOVA followed by post hoc Tukey's analysis. Tabulated data can be found in Data S8.

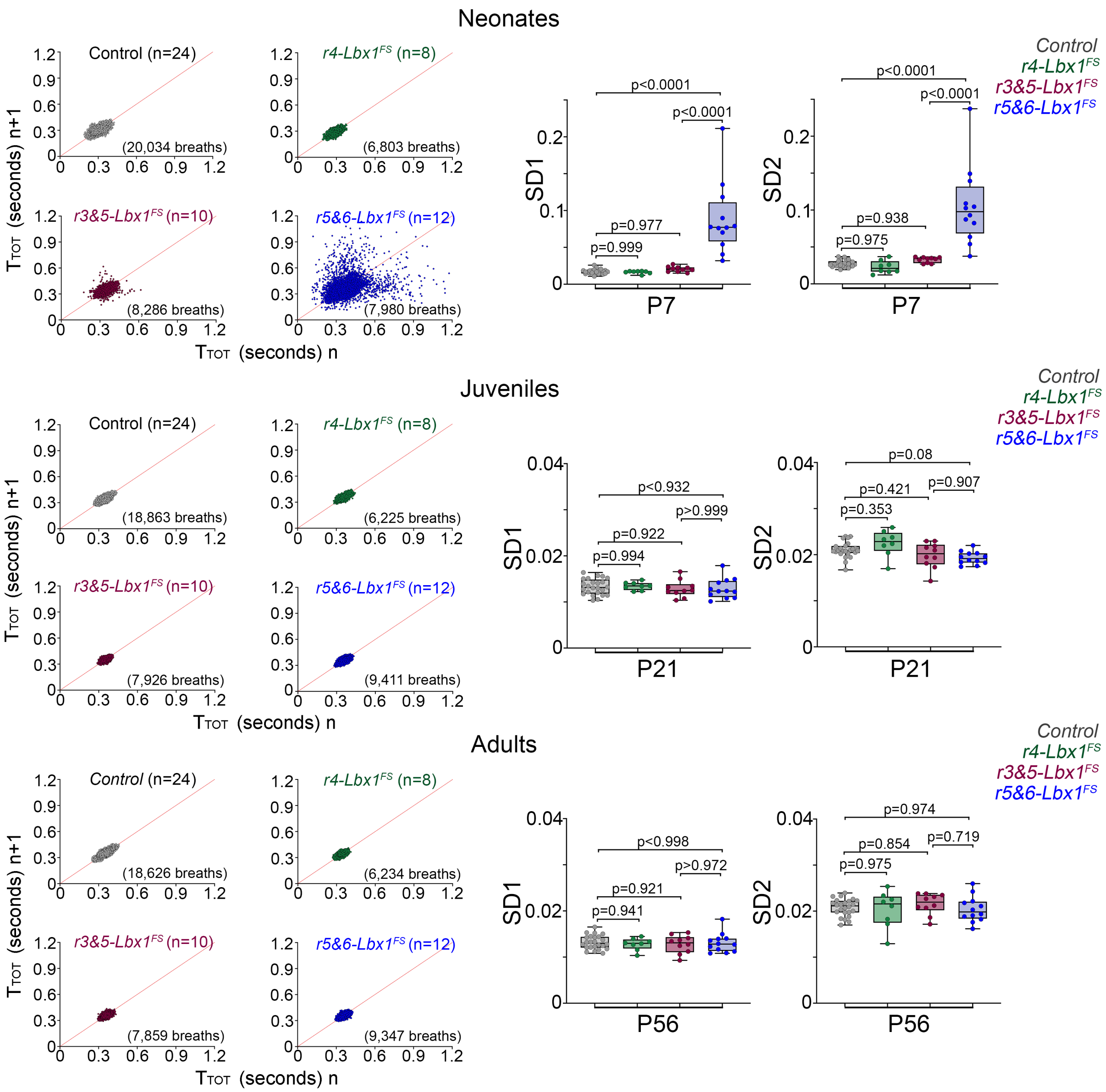

Fig. S13. Respiratory instability displayed by *r4-Lbx1^{FS}*, *r3&5-Lbx1^{FS}*, and *r5&6-Lbx1^{FS}* mice at different ages. Quantification of respiratory instability for *Control* (n=24*)*, *(Tg)Hoxb1$^{Cre/+}$;Lbx1$^{FS/lox}$* (*r4-Lbx1^{FS}*; n=8), *Egr2$^{Cre/+}$;Lbx1$^{FS/lox}$* (*r3&5-Lbx1^{FS}*; n=10), and *(Tg)Hoxa3$^{Cre/+}$;Lbx1$^{FS/lox}$* (*r5&6-Lbx1^{FS}*; n=12) mice while breathing ambient air at the indicated stages. Each dot represents individual breaths in the Poincaré plots (left panels), and each dot displayed in the standard deviation (SD) 1 and 2 plots represents the mean of individual mice analyzed. Significance was determined using one-way ANOVA followed by post hoc Tukey's analysis. Tabulated data can be found in Data S8.

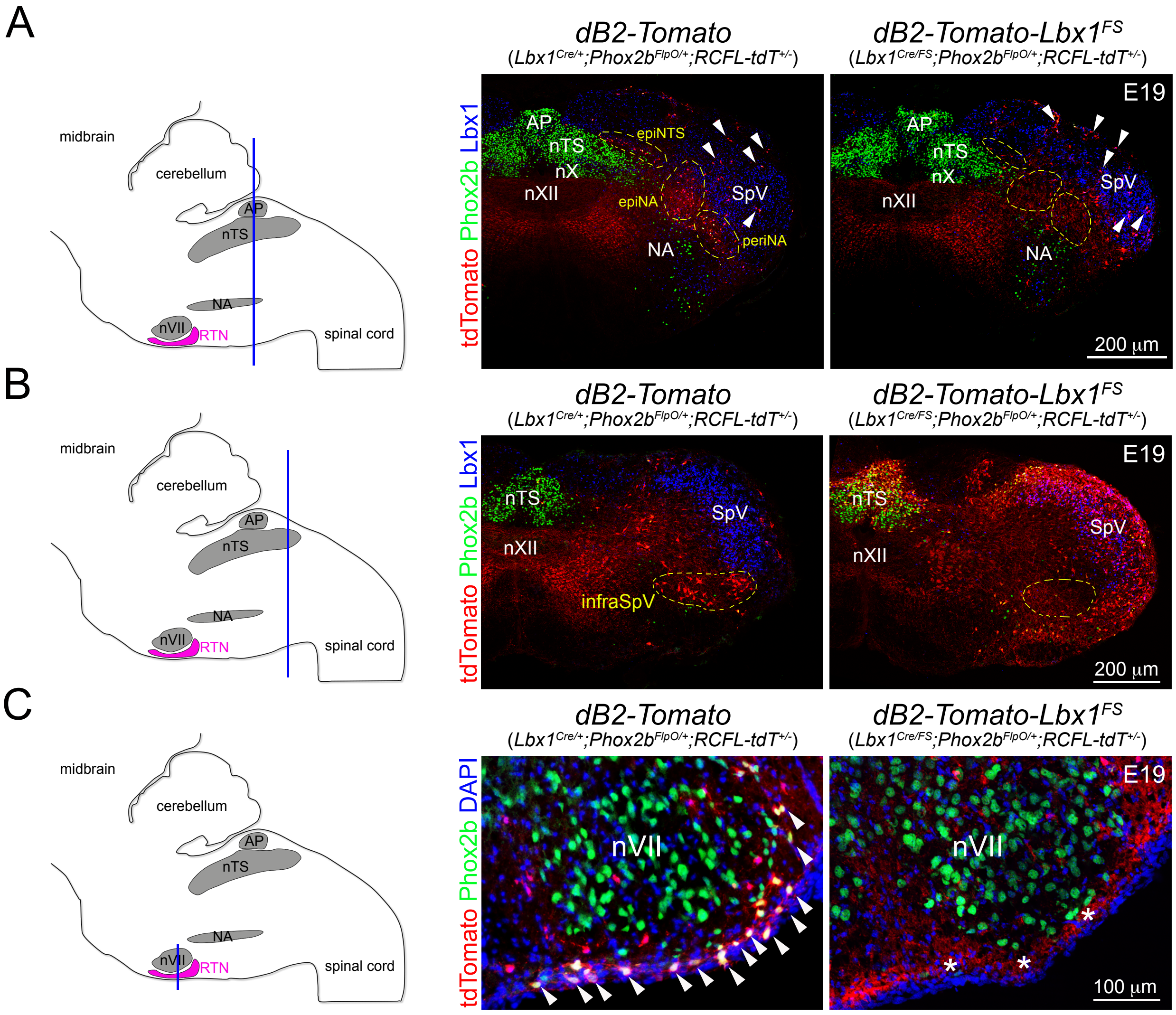

Fig. S14. Misspecification of caudal dB2 neurons in the *Lbx1^FS^* background. (**A-C**) Left, sagittal schemas showing the transverse section planes (blue lines) illustrated on the right. (**A, B**) Right, immunofluorescence characterization of caudal dB2 neuron subgroups (epiNTS, epiNA, periNA and infraSpV; marked in yellow dashed lines) in *dB2-Tomato* and *dB2-Tomato-Lbx1^FS^* mice at E19. The precise genotypes are displayed in brackets. The transverse sections were stained against red fluorescent protein (to detect tdTomato, red), Lbx1 (blue, false color) and Phox2b (green). The area postrema (AP), nucleus tractus solitarius (nTS), spinal trigeminal nucleus (SpV), as well as the vagal (nX), hypoglossal (nXII) and ambiguus (NA) motor nuclei are illustrated for anatomical orientation. Note that while epiNTS, epiNA, and periNA dB2 neurons are lacking in *dB2-Tomato-Lbx1^FS^* mice, somaV neurons appeared unchanged (arrowheads in A). Due to space restrictions, some tdTomato+ dB2 neurons might not be included within the marked dashed areas. (**C**) Right, immunofluorescence characterization of dB2 retrotrapezoid nucleus (RTN; arrowheads in C) neurons in *dB2-Tomato* and *dB2-Tomato-Lbx1^FS^* mice at E19. The transverse sections were stained against red fluorescent protein (to detect tdTomato, red) and Phox2b (green). DAPI (blue) was used to counterstain. The facial (nVII) motor nucleus is indicated for anatomical orientation. Note that dB2 RTN (tdTomato+) neurons are absent in *dB2-Tomato-Lbx1^FS^* mice. The residual tdTomato staining in the RTN* (asterisks) area of *dB2-Tomato-Lbx1^FS^* mice appears to be axonal from misspecified dB2* neurons.

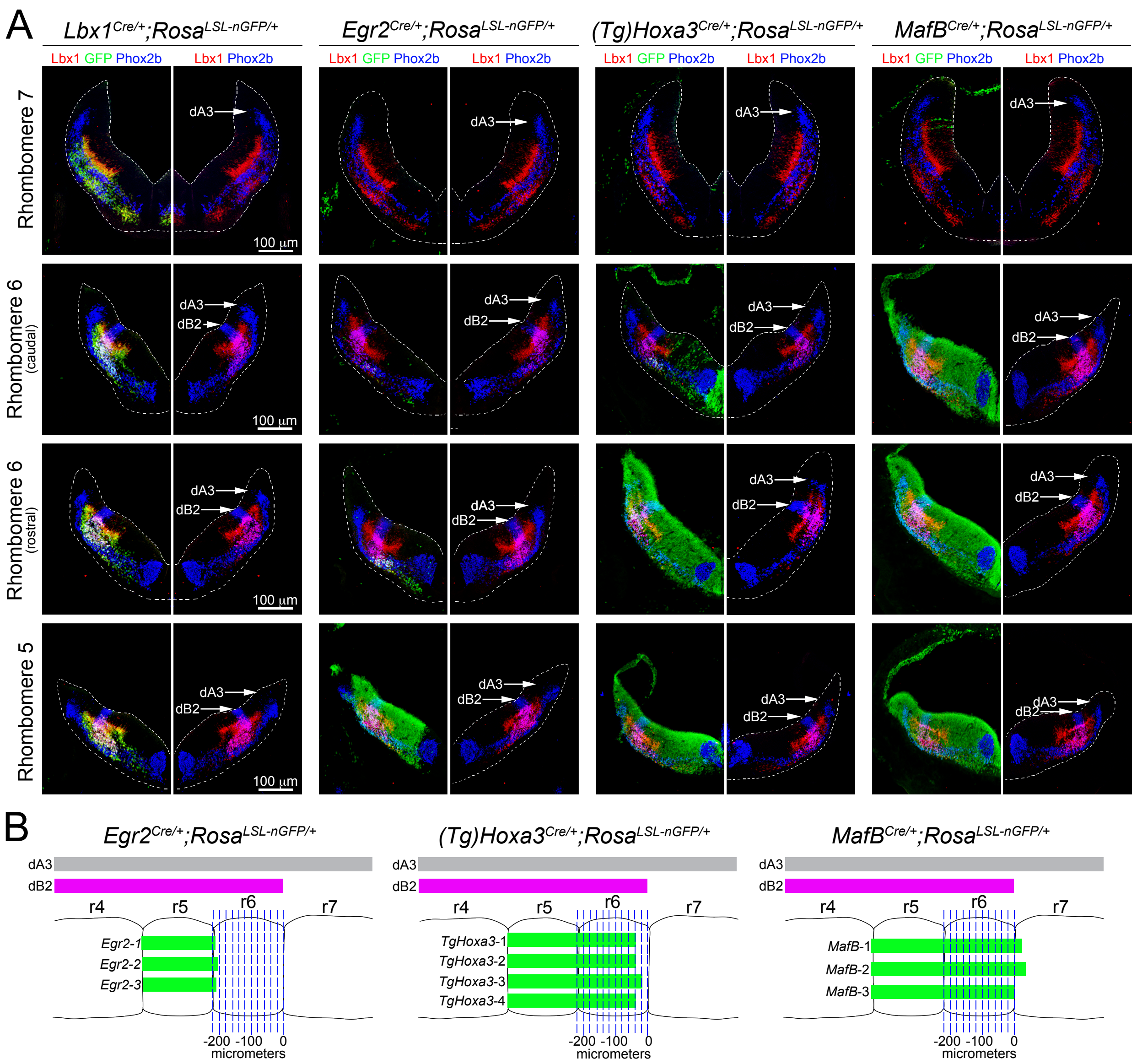

Fig. S15. Characterization of *(Tg)Hoxa3^Cre^* and *MafB^Cre^* recombination patterns in early development. (**A-B**) Characterization of the recombination patterns produced by the *Lbx1^Cre^*, *Egr2^Cre^*, *(Tg)Hoxa3^Cre^* and *MafB^Cre^* driver lines at E11.5, a time point in which dB2 neurons become specified (reviewed in *30* and *33*). Recombination was assessed using the *Rosa^LSL-nGFP^* reporter allele. This analysis included rhombomeres 5 to rhombomere 7. Please note that dA3 (Lbx1-/Phox2b+) neurons extend from rhombomere 4 to 7, while dB2 (Lbx1+/Phox2b+) neurons extend from rhombomere 2 to rhombomere 6, but not into rhombomere 7 (reviewed in *30* and *33*). (**A**) Transverse sections of the developing hindbrain for the indicated genotypes. The sections were stained with antibodies against GFP (green), Lbx1 (red) and Phox2b (blue, false color). The photographs are shown with Lbx1, GFP and Phox2b merged signals (on the left) and Lbx1 and Phox2b signals (on the right) for a better visualization of dA3 and dB2 neurons. (**B**) Schematic display of the recombination patterns observed in *Egr2^Cre/+^;Rosa^LSL-nGFP/+^* (n=3), *(Tg)Hoxa3^Cre^;Rosa^LSL-nGFP/+^* (n=4), and *MafB^Cre^;Rosa^LSL-nGFP/+^* (n=3), embryos at E11.5. The blue dashed lines indicate consecutive sections (spaced 20 μm apart) analyzed from the caudal border of rhombomere 6. In this analysis, we considered the caudal border of rhombomere 6 (denoted as 0 micrometers) to be the region in which dB2 (Phox2b+) progenitor cells were first seen. The green bars indicate the recombination patterns seen in each of the analyzed embryos.

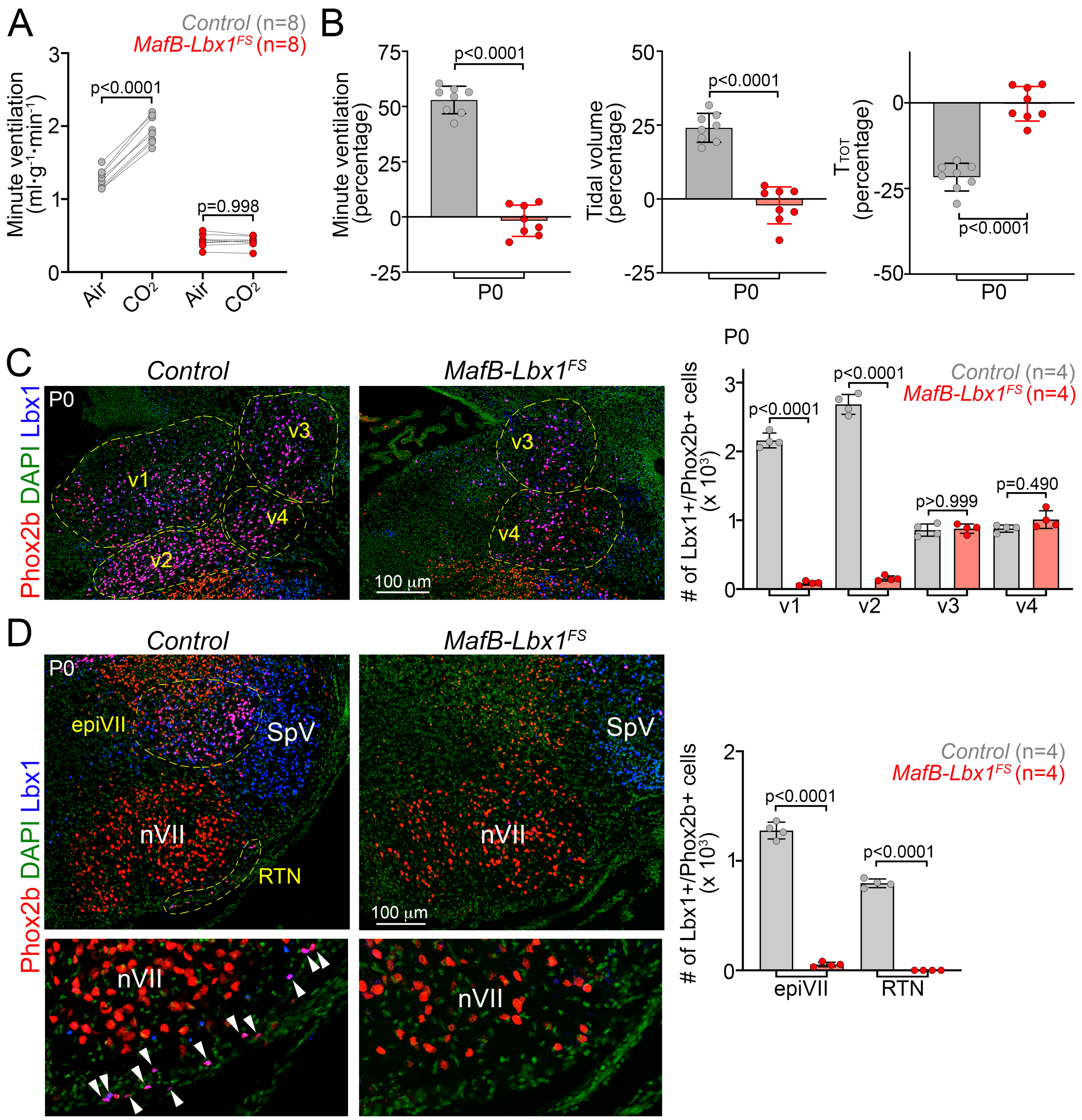

Fig. S16. Respiratory patterns and histology of *MafB-Lbx1^{FS}* newborn mice. (**A**) Quantification of minute ventilation in *Control* (n=8 mice) and *MafB-Lbx1^{FS}* (n=8 mice) newborns while breathing ambient air or high levels of CO_2 (8% in the air, hypercarbia). (**B**) Respiratory responses to hypercarbia expressed as percentage of change relative to the baseline (ambient air). Change of minute ventilation (left), tidal volume (middle) and respiratory cycle length (right) displayed by *Control* (n=8 mice) and *MafB-Lbx1^{FS}* (n=8 mice) newborns. (**C-D**) Histological characterization and quantification of vestibular (v1-v4) dB2 neurons as well as epifacial (epiVII) and retrotrapezoid nucleus (RTN) neurons in *Control* (n=4 mice) and *MafB-Lbx1^{FS}* (n=4 mice) newborns. Arrowheads denote RTN neurons. Significance was determined using one-way ANOVA followed by post hoc Tukey's analysis for group comparison or two tailed t-test for pair comparison. Tabulated data can be found in Data S8.

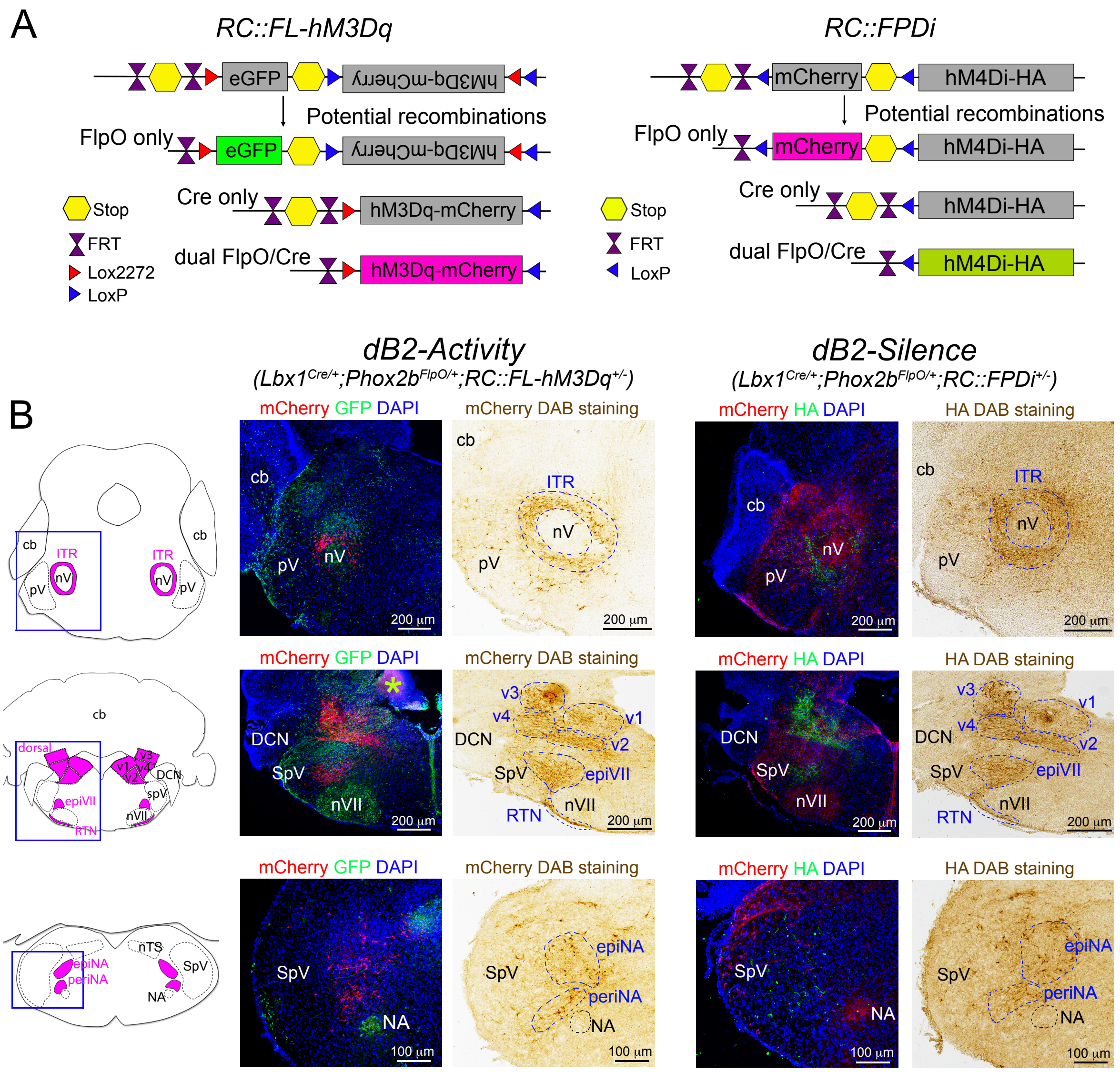

Fig. S17. Intersectional expression of hM3Dq (*dB2-Activity*) and hM4Di (*dB2-Silence*) in dB2 neurons. (**A**) Left, genetic strategy to express *hM3Dq* in dB2 neurons; the intersectional *RC::FL-hM3Dq* dual-recombination allele expresses an mCherry-fused hM3Dq receptor upon the excision of two stop cassettes flanked by *FRT and Lox* sites. These stop cassettes were excised by *Lbx1*^{Cre} and *Phox2b*^{FlpO}. Note that FlpO recombination alone allows for the activation of enhanced *GFP* (eGFP) expression but not the *hM3Dq-mCh*erry receptor, while Cre expression alone removes the *eGFP* sequence, reverts the *hM3Dq-mCherry* sequence, but does not activate the expression of the *hM3Dq-mCherry* receptor. Only dual Cre/Flpo recombination allows for the expression of the *hM3Dq-mCherry* receptor. Right, genetic strategy to express *hM4Di* in dB2 neurons, the intersectional *RC::FPDi* dual-recombination allele expresses an HA-tagged hM4Di receptor upon the excision of two stop cassettes flanked by *FRT and LoxP* sites. These stop cassettes were excised by *Lbx1*^{Cre} and *Phox2b*^{FlpO}. Note that FlpO recombination alone allows for the activation of *mCherry* expression but not the *hM4Di-HA* receptor, while Cre recombination alone removes the *mCherry* sequence but does not activate the expression of the *hM4Di-HA* receptor. Only dual Cre/FlpO recombination allows for the expression of the *hM4Di-HA* receptor. (**B**) Left panels, schematic views of the section planes displayed on the right (marked by the blue rectangles). Analyzed dB2 neuron subgroups: intertrigeminal (ITR), vestibular (v1-v4), epifacial (epiVII), retrotrapezoid (RTN), epi- (epiNA) and peri- (periNA) nucleus ambiguus are indicated in magenta. The cerebellum (cb), dorsal cochlear nucleus (DCN), nucleus ambiguus (NA) facial motor nucleus (nVII), nucleus tractus solitarius (nTS), as well as the principal (pV), spinal (SpV) and motor (nV) trigeminal nuclei are indicated for orientation. Center panels, transverse brainstem sections taken from newborn *dB2-Activity* mice stained with DAPI (blue) and antibodies against GFP (green) and RFP (to detect mCherry, red; immunofluorescence images) or only with antibodies against RFP and processed for 3, 3′-diaminobenzidine (DAB) immunohistochemistry. Right panels, transverse brainstem sections taken from newborn *dB2-Silence* mice stained with DAPI (blue) and antibodies against HA (green) and RFP (to detect mCherry, red; immunofluorescence images) or only with antibodies against HA and processed for DAB immunohistochemistry. Magnifications for dB2 RTN neurons can be found in fig. S18.

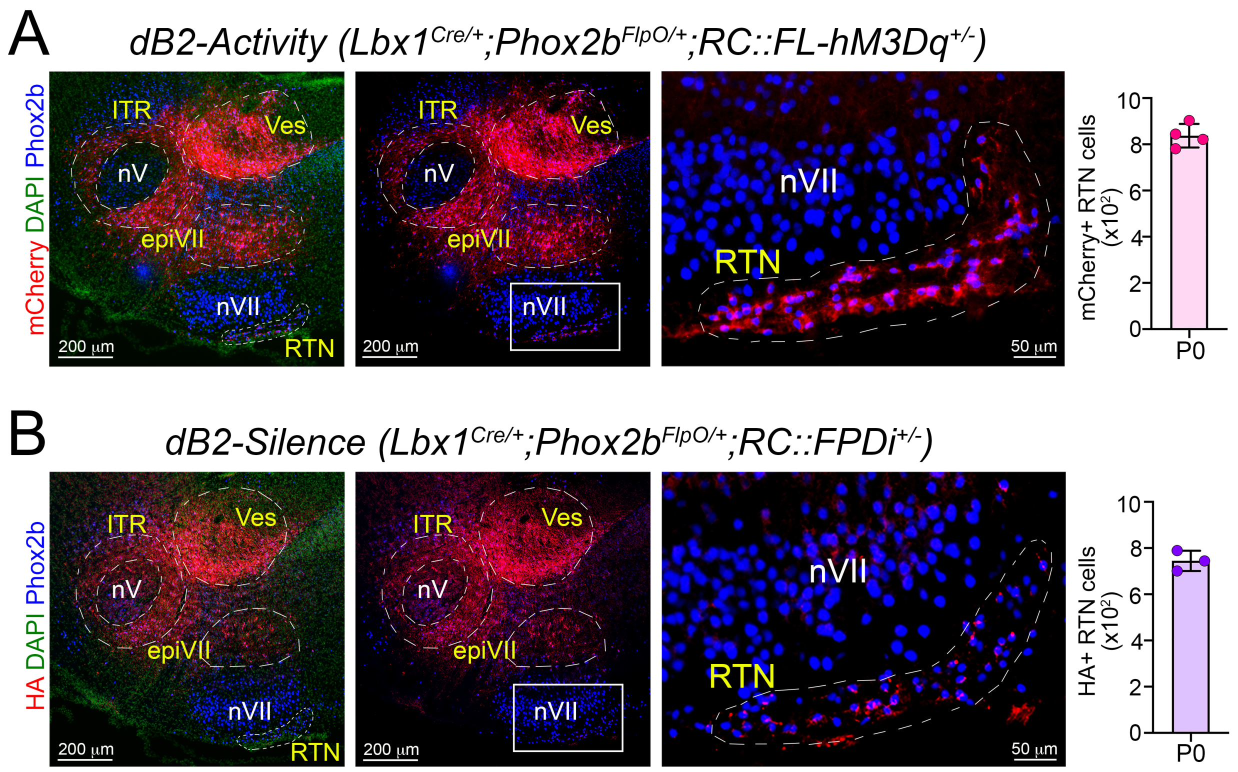

Fig. S18. Intersectional expression of hM3Dq (*dB2-Activity* mice) and hM4Di (*dB2-Silence* mice) in RTN neurons. (**A**) Histological analysis and quantification of dB2 retrotrapezoid nucleus (RTN) neurons expressing mCherry, the reporter for hM3Dq DREADD receptor expression in *dB2-Activity* newborn (n=4) mice. The sagittal section depicted was stained with antibodies against the red fluorescent protein (to detect mCherry, red) and Phox2b (blue, false color). The section was counterstained with DAPI (green, false color). (**B**) Histological analysis and quantification of dB2 RTN neurons expressing HA-tag, the reporter for hM4Di DREADD receptor expression in *dB2-Silence* newborn (n=3) mice. The illustrated sagittal section was stained with antibodies against HA (red, false color) and Phox2b (blue, false color). The section was counterstained with DAPI (green, false color). The white dashed lines delineate dB2 neuron subgroups such as the intertrigeminal region (ITR), the epifacial (epiVII) group, the vestibular populations (v1-v4) and the RTN. The facial (nVII) and trigeminal (nV) motor nuclei are indicated for anatomical orientation. The boxed areas in the middle panels are magnified on the right. Tabulated data can be found in Data S8.

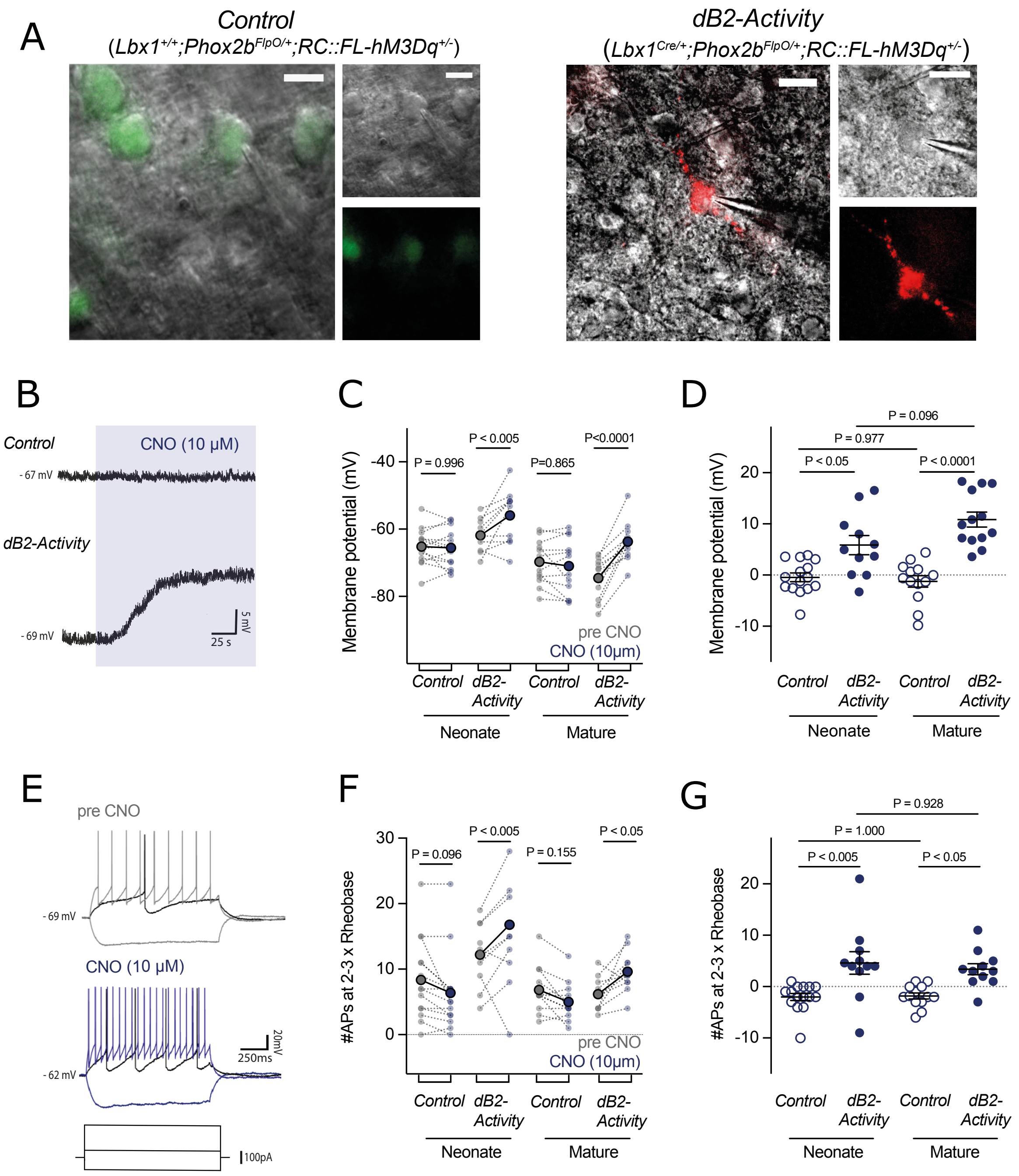

Fig. S19. Figure caption in next page.

Fig. S19. Chemogenetic activation of dB2 neurons. (**A**) A patch-clamped *control* (*Lbx1*$^{+/+}$*;Phox2b*$^{FlpO/+}$*;RC::FL-hM3Dq*$^{+/-}$; GFP+) neuron (left) and a DREADD-expressing dB2 (*Lbx1*$^{Cre/+}$*;Phox2b*$^{FlpO/+}$*;RC::FL-hM3Dq*$^{+/-}$; *dB2-Activity*; mCherry+) neuron. Scale bars, 20 µm. (**B**) Representative membrane potential traces recorded from a *control* (top) and a DREADD-expressing dB2 neuron (bottom) in the presence of CNQX (20 µM) and D-AP5 (25 µM). Bath application of CNO (10 µM) depolarized the DREADD-expressing dB2 neuron but had no effect on the membrane potential of the *control* neuron. (**C**) Individual neuron and population average data of the membrane potential before (pre) and upon CNO application in DREADD-expressing *dB2-Activity* neurons (neonate: n=10 cells from 5 mice; –61.9 ± 4.6 mV to -55.9 ± 8.0 mV; mature: n=12 cells from 4 mice; –74.5 ± 5.6 mV to –63.7 ± 5.6 mV) and *control* neurons (neonate: n=14 cells from 5 mice; –65.2 ± 5.1 mV to –65.6 ± 5.4 mV; mature: n = 12 cells from 2 mice; –69.7 ± 6.7 mV to –71.0 ± 7.7 mV). (**D**) Direct comparison of the CNO effects on membrane potential in *dB2-Activity* (neonate: 6.0 ± 1.4 mV; mature: 10.8 ± 1.3 mV) and *control* (neonate: –0.4 ± 1.2 mV; mature: –1.3 ± 1.3 mV) neurons. (**E**) Representative voltage responses to 1 second (s) rectangular current injections of –100, 50, and 225 pA, in somatic whole-cell recordings from a *dB2-Activity* neuron. Compared to the pre-CNO condition (gray traces), CNO (10 µM, blue traces) increased the number of action potentials (AP) in response to the suprathreshold current injections. (**F**) Individual neuron and population average data of the action potential number in response to current injections with amplitudes between 2 and 3 times of the calculated rheobase. In both age groups, CNO increases the action potential number of *dB2-Activity* neurons in response to the suprathreshold stimulus (neonate: 12.2 ± 4.6 to 16.8 ± 9.6; mature: 6.2 ± 2.4 to 9.6 ± 3.1), with negligible effects on *control* neurons (neonates: 8.4 ± 6.0 to 6.4 ± 5.8; mature: 6.8 ± 3.4 to 5.0 ± 2.8). (**G**) Direct comparison of the CNO-induced alterations of action potential firing in response to a suprathreshold stimulus (2-3 times the rheobase) in *dB2-Activity* neurons (neonate: 4.6 ± 1.4; mature: 3.4 ± 1.4) and *control* neurons (neonate: –2.0 ± 1.2 mV; mature: –1.8 ± 1.3). Every faint dot in the graphs represents an individual recorded neuron, while the darkened dots represent the mean of the population data. *Control* and *dB2-Activity* neurons were recorded from the medial vestibular (v1 and v2) and epifacial (epiVII) dB2 neuron subgroups. Significance was determined using two-way nested ANOVA with Šidák post hoc test. Note that CNO induces depolarization and changes in membrane potential in DREADD-expressing *dB2-Activity* neurons that do not significantly differ between neonate and mature neurons. Tabulated data can be found in Data S8.

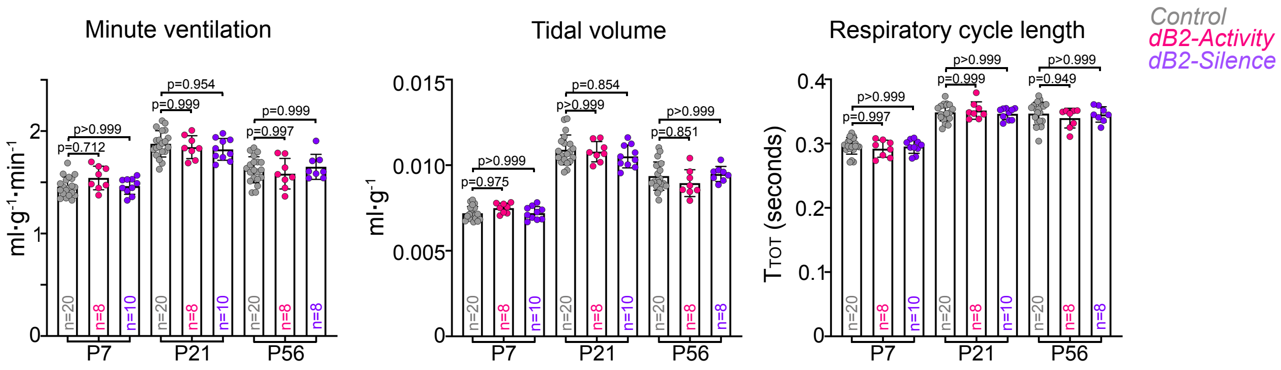

Fig. S20. Breathing analysis of *Control*, *dB2-Activity* and *dB2-Silence* mice before CNO treatment. Quantification of minute ventilation, tidal volumes, and respiratory cycle lengths (T_{TOT}), in *Control*, *dB2-Activity* and *dB2-Silence* mice before CNO treatment at the stages studied (P7, P21 and P56). Respiratory recordings were taken in ambient air. The precise numbers of mice analyzed (n) are displayed in the bars. Every dot represents the mean of individual animals. Significance was determined using one-way ANOVA followed by post hoc Tukey's analysis. Tabulated data can be found in Data S8

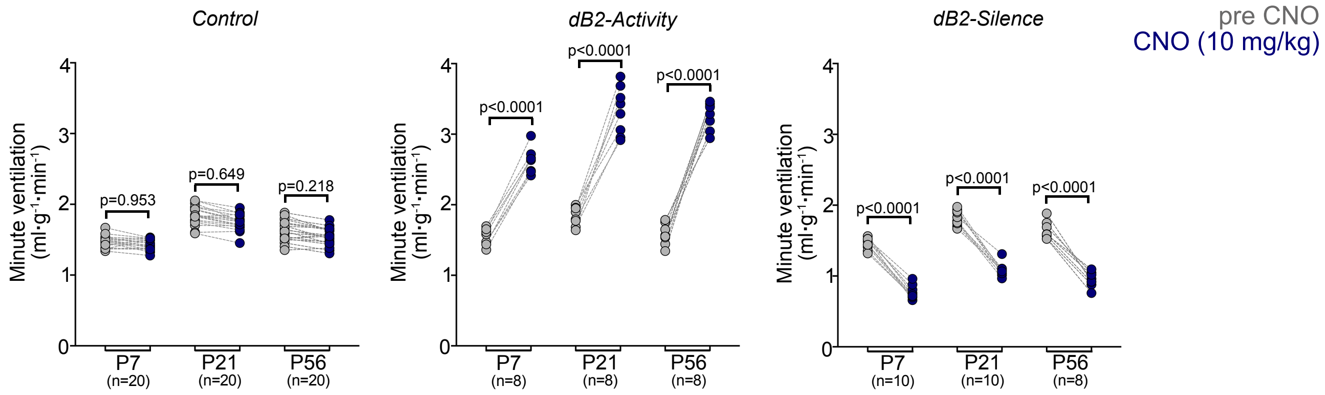

Fig. S21. Ventilatory changes caused by the activation or inhibition of dB2 neuron activity. Quantification of minute ventilation in *Control*, *dB2-Activity* and *dB2-Silence* mice before (pre; gray) and after CNO (10 mg/kg; blue) treatment at the studied stages (P7, P21 and P56). Respiratory recordings were taken in ambient air. The precise numbers of mice analyzed (n) are displayed underneath the stages analyzed. Every dot represents the mean of individual animals. Significance was determined using one-way ANOVA followed by post hoc Tukey's analysis. Tabulated data can be found in Data S8.

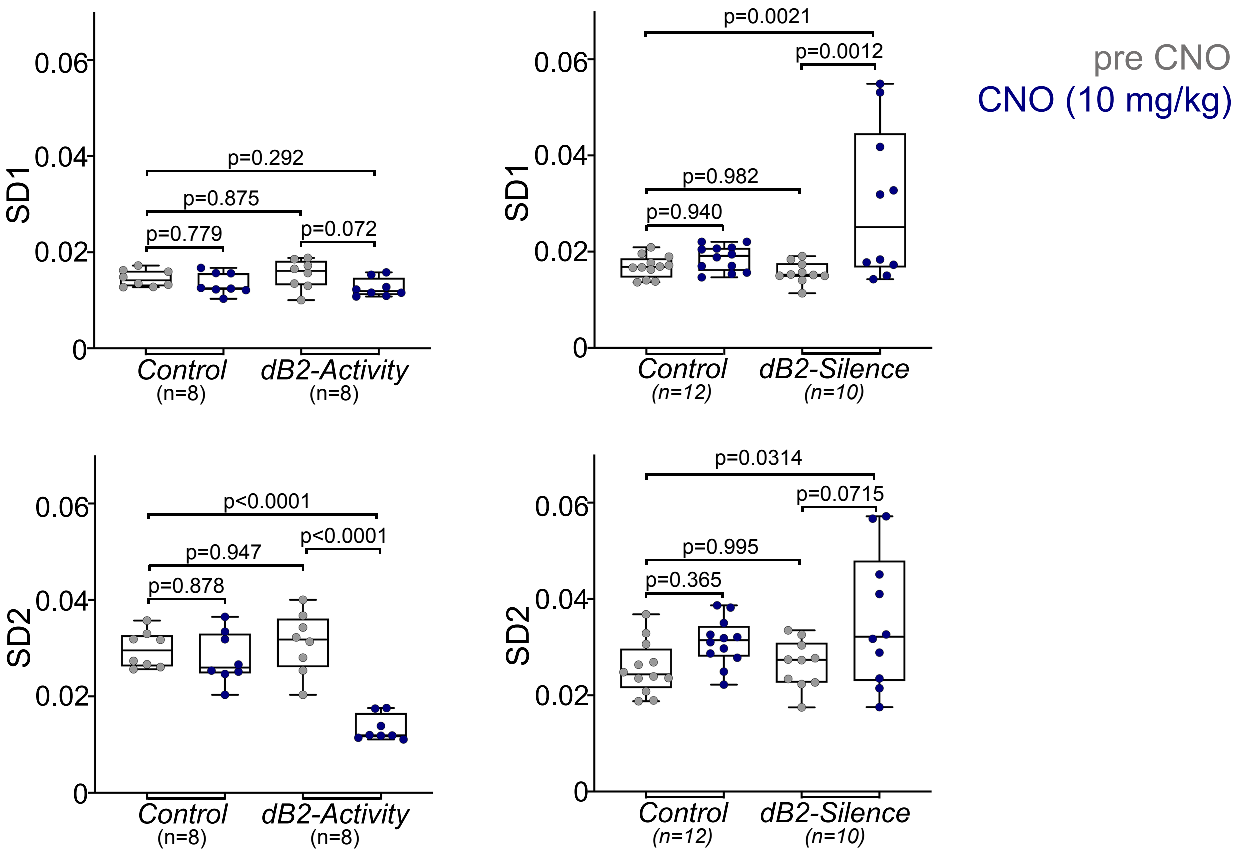

Fig. S22. Breathing instability of *Control*, *dB2-Activity*, and *dB2-Silence* neonates. Quantification of standard deviation (SD) 1 and SD2 in *Control, dB2-Activity, and dB2-Silence* neonates before (pre; gray) and after CNO (10 mg/kg; blue) treatment. Every dot represents the mean of individual animals. The precise numbers of mice analyzed (n) are displayed in brackets underneath the studied genotypes. Significance was determined using one-way ANOVA followed by post hoc Tukey's analysis. Tabulated data can be found in Data S8.

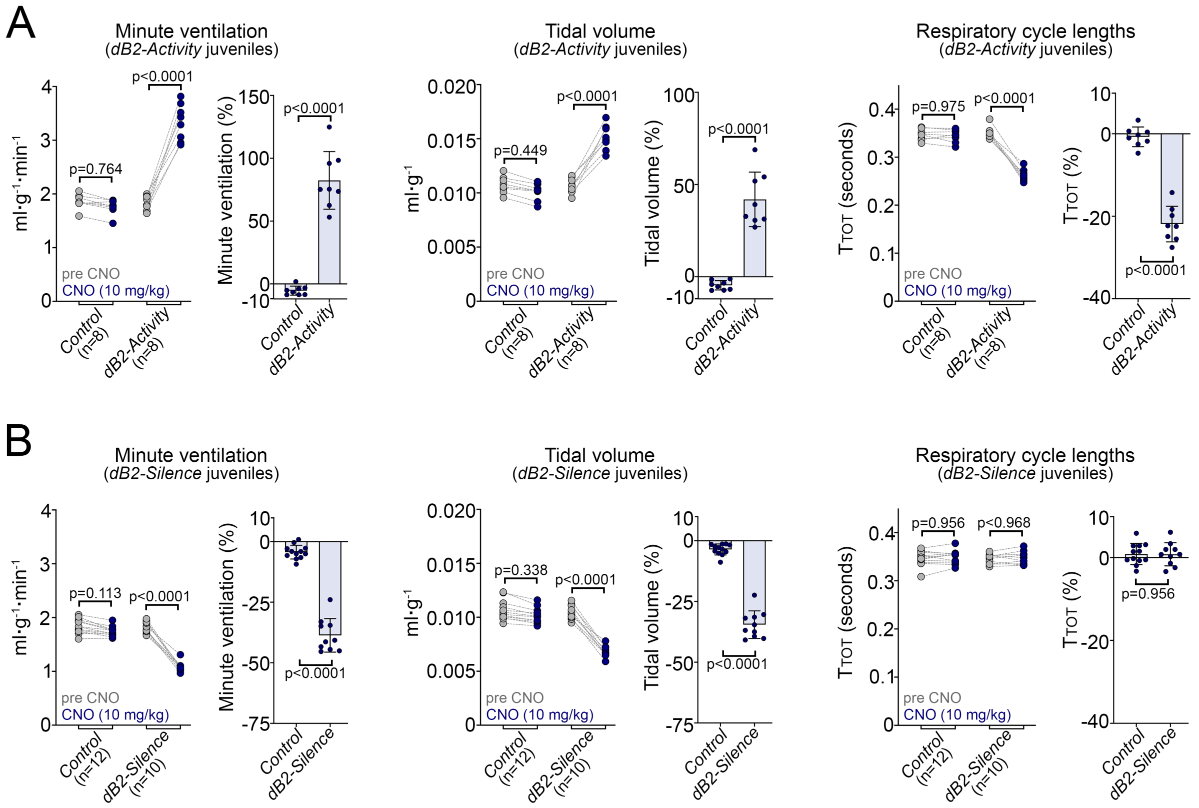

Fig. S23. Ventilatory changes caused by the activation or inhibition of dB2 neuron activity in juvenile mice. (**A**) Quantification of minute ventilation, tidal volumes, and respiratory cycle lengths (T_{TOT}) in *Control* and *dB2-Activity* juvenile (P21) mice while breathing ambient air, before (pre; gray) and after CNO (10 mg/kg; blue) treatment. Changes of minute ventilation, tidal volumes and T_{TOT}, expressed as percentage of change relative to the baseline before (pre) CNO treatment, are presented at the right of the main plots. (**B**) Quantification of minute ventilation, tidal volumes, and respiratory cycle lengths (T_{TOT}) in *Control* and *dB2-Silence* juvenile (P21) mice while breathing ambient air, before (pre; gray) and after CNO (10 mg/kg; blue) treatment. Changes of minute ventilation, tidal volumes and T_{TOT}, expressed as percentage of change relative to the baseline before CNO treatment, are presented at the right of the main plots. The precise number of mice analyzed (n) are displayed in brackets. Every dot represents the mean of individual animals. Significance was determined using one-way ANOVA followed by post hoc Tukey's analysis for group comparison or two tailed t-test for pair comparison. Tabulated data can be found in Data S8.

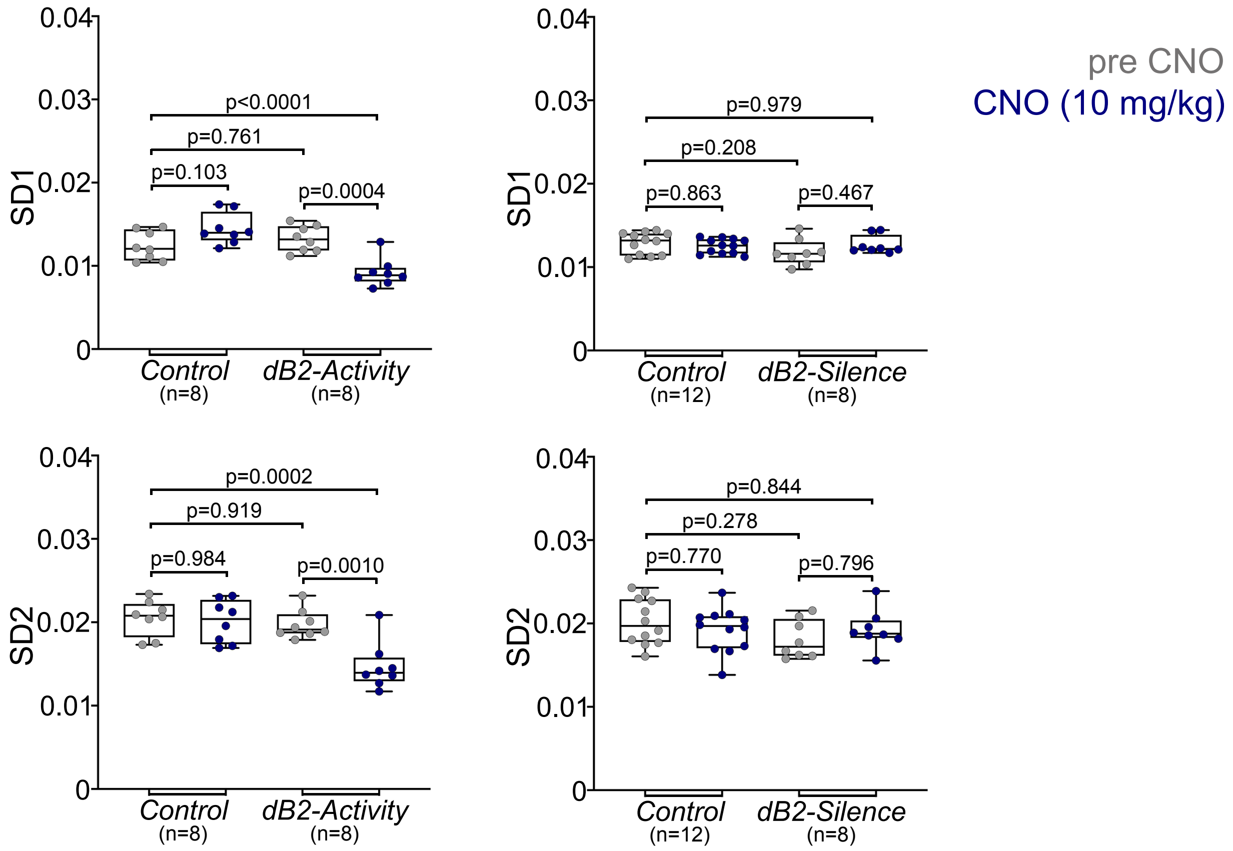

Fig. S24. Breathing instability of *Control*, *dB2-Activity*, and *dB2-Silence* adult. Quantification of standard deviation (SD) 1 and SD2 in *Control, dB2-Activity, and dB2-Silence* adult mice before (pre; gray) and after CNO (10 mg/kg; blue) treatment. Every dot represents the mean of individual animals. The precise numbers of mice analyzed (n) are displayed in brackets underneath the studied genotypes. Significance was determined using one-way ANOVA followed by post hoc Tukey's analysis. Tabulated data can be found in Data S8.

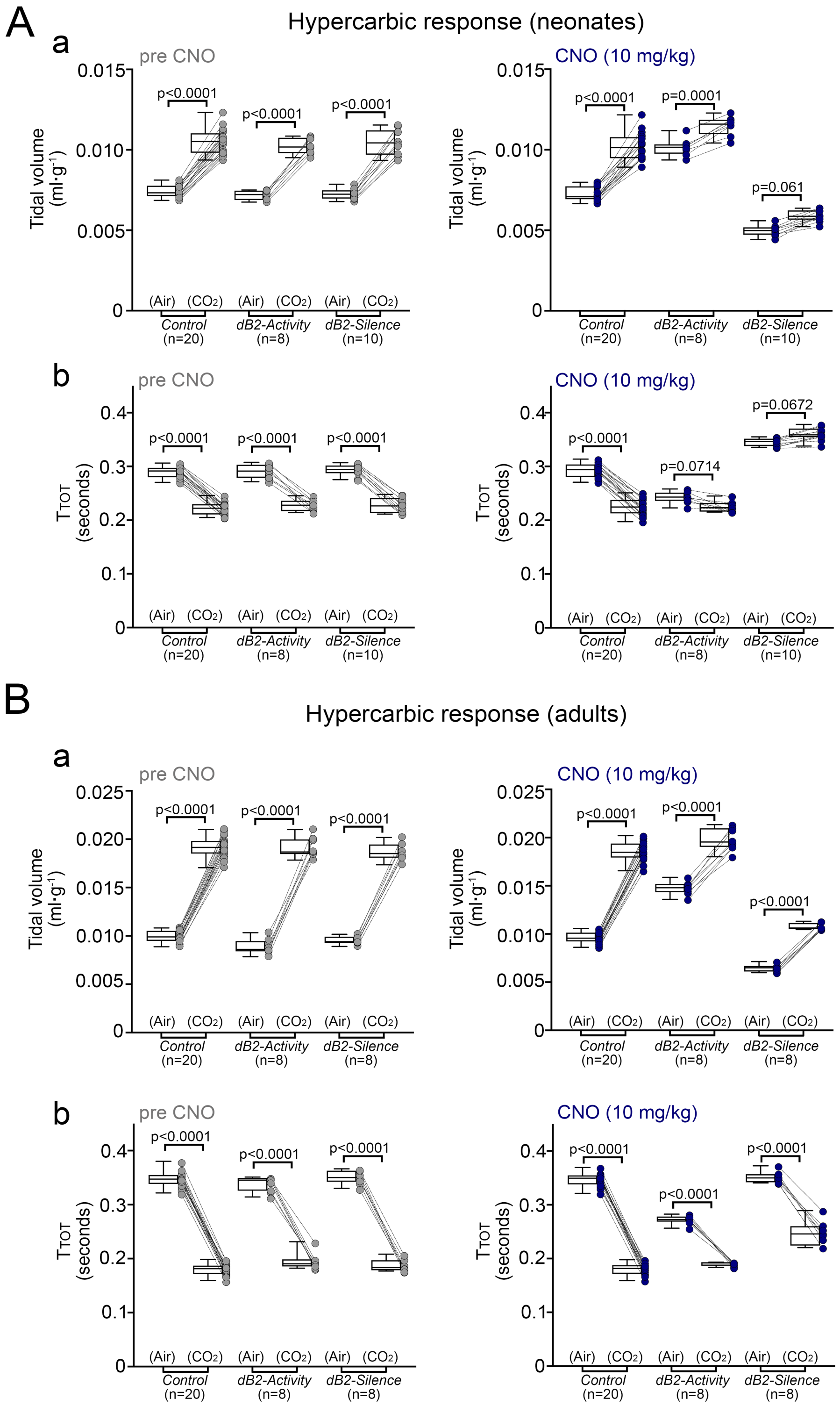

Fig. S25. dB2 neurons regulate the hypercarbic reflex. (**A, B**) Hypercarbic response seen in *Control*, *dB2-Activity*, and *dB2-Silence* neonate (in A) and adult (in B) mice before (pre; gray) and after CNO treatment (10 mg/kg; blue). Respiration was analyzed in ambient air (air) and in hypercarbia (CO_2) for five minutes (as indicated in red in Fig. 8A). The number (n) of mice analyzed is displayed underneath the studied genotypes. Quantification of tidal volumes (a in A and B) and respiratory cycle lengths (b in A and B) in ambient air (air) and hypercarbia (CO_2) for the indicated genotypes, stages, and conditions. Every dot represents the mean of individual mice. Significance was determined using one-way ANOVA followed by post hoc Tukey's analysis. Tabulated data can be found in Data S7.

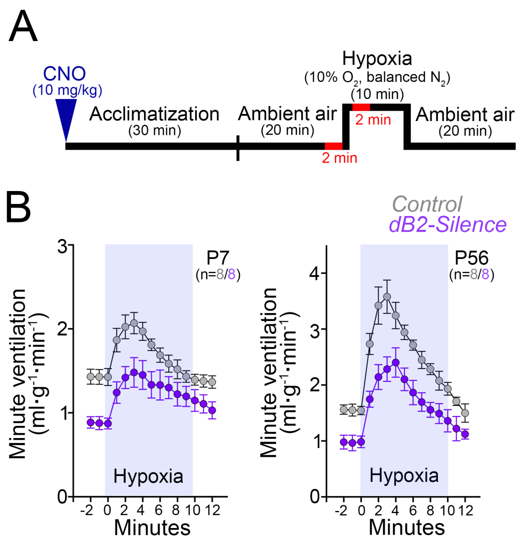

Fig. S26. dB2 neurons are dispensable for the hypoxic reflex. (**A**) Diagram illustrating the protocol used to induce a hypoxic response in mice. Respiration of mice was quantified in ambient air and in hypoxia for two minutes each (denoted in red, quantified in C). (**B**) Minute ventilation values in ambient air or hypoxia (highlighted). Each circle represents the mean ± standard deviation over a 60-second period. (**C**) Respiratory responses to hypoxia expressed as percentage of change relative to the baseline (ambient air). Changes of minute ventilation (left), tidal volume (middle), and respiratory cycle lengths (T_{TOT}, right) for the indicated stages and conditions are displayed for the indicated ages and conditions. Significance was determined using one-way ANOVA followed by post hoc Tukey's analysis. Tabulated data can be found in Data S8.

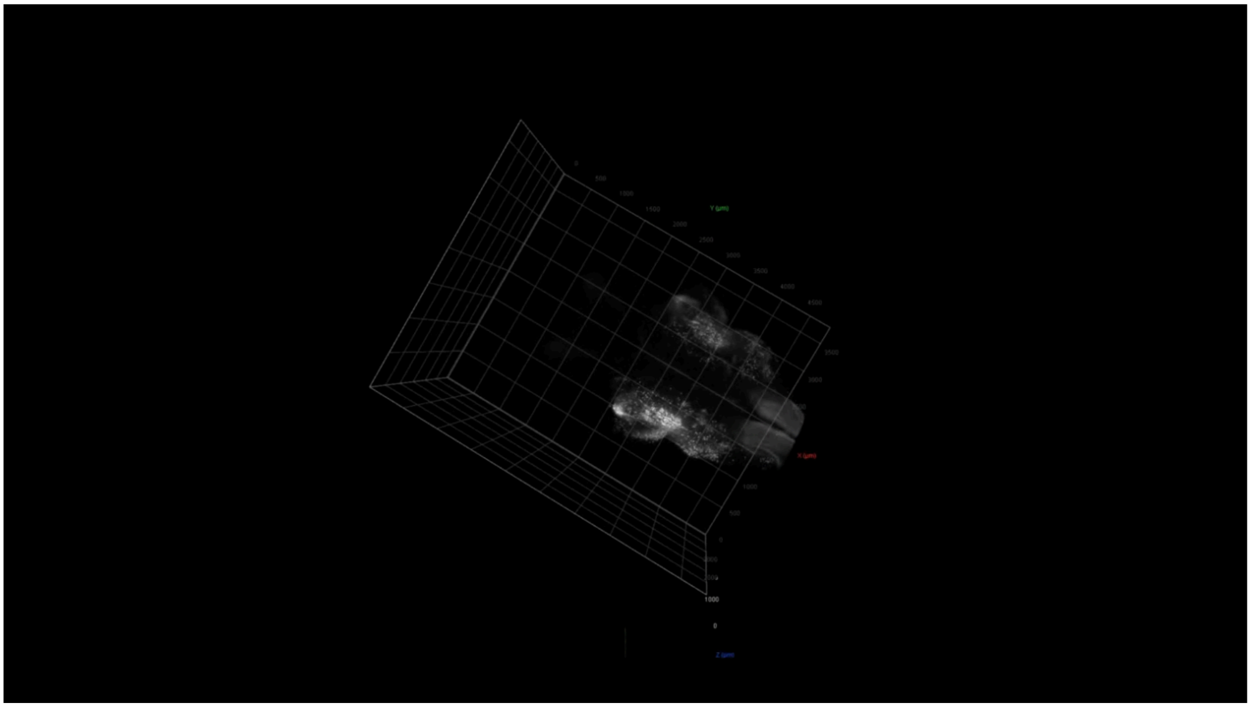

Movie S1. 3D reconstruction of a *dB2-Tomato* ($Lbx1^{Cre/+}$;$Phox2b^{FlpO/+}$;$RCFL\text{-}tdT^{+/-}$) brainstem at birth. tdTomato+ neurons are displayed in white. Subgroups of dB2 neurons with active Lbx1 and Phox2b protein expression are indicated in yellow (see text). Due to space limitations some neurons belonging to the indicated nuclei might not be included within the marked areas.

Data S1. Tabulated Data for Fig. 1. Excel spreadsheet.

Data S2. Tabulated Data for Fig. 3. Excel spreadsheet.

Data S3. Tabulated Data for Fig. 4 and fig. S11. Excel spreadsheet.

Data S4. Tabulated Data for Fig. 5. Excel spreadsheet.

Data S5. Tabulated Data for Fig. 6. Excel spreadsheet.

Data S6. Tabulated Data for Fig. 7. Excel spreadsheet.

Data S7. Tabulated Data for Fig. 8 and fig. S25. Excel spreadsheet.

Data S8. Tabulated Data for figs. S2, S4, S6-9, S12, S13, S16, S18-S24, S26. Excel spreadsheet.
